# Leucine-Rich repeat receptor kinases are sporadically distributed in eukaryotic genomes

**DOI:** 10.1186/1471-2148-11-367

**Published:** 2011-12-20

**Authors:** Anne Diévart, Nicolas Gilbert, Gaétan Droc, Agnès Attard, Matthieu Gourgues, Emmanuel Guiderdoni, Christophe Périn

**Affiliations:** 1 CIRAD, UMR AGAP, F-34398 Montpellier, France; 2 Institut de Génétique Humaine, CNRS, UPR 1142, 141 rue de la Cardonille, 34396 Montpellier cedex 5, France; 3 INRA, CNRS, Université Nice-Sophia Antipolis, UMR Interactions Biotiques et Santé Végétale, Sophia Antipolis, France

## Abstract

**Background:**

Plant leucine-rich repeat receptor-like kinases (LRR-RLKs) are receptor kinases that contain LRRs in their extracellular domain. In the last 15 years, many research groups have demonstrated major roles played by LRR-RLKs in plants during almost all developmental processes throughout the life of the plant and in defense/resistance against a large range of pathogens. Recently, a breakthrough has been made in this field that challenges the dogma of the specificity of plant LRR-RLKs.

**Results:**

We analyzed ~1000 complete genomes and show that LRR-RK genes have now been identified in 8 non-plant genomes. We performed an exhaustive phylogenetic analysis of all of these receptors, revealing that all of the LRR-containing receptor subfamilies form lineage-specific clades. Our results suggest that the association of LRRs with RKs appeared independently at least four times in eukaryotic evolutionary history. Moreover, the molecular evolutionary history of the LRR-RKs found in oomycetes is reminiscent of the pattern observed in plants: expansion with amplification/deletion and evolution of the domain organization leading to the functional diversification of members of the gene family. Finally, the expression data suggest that oomycete LRR-RKs may play a role in several stages of the oomycete life cycle.

**Conclusions:**

In view of the key roles that LRR-RLKs play throughout the entire lifetime of plants and plant-environment interactions, the emergence and expansion of this type of receptor in several phyla along the evolution of eukaryotes, and particularly in oomycete genomes, questions their intrinsic functions in mimicry and/or in the coevolution of receptors between hosts and pathogens.

## Background

**Figure 1:**
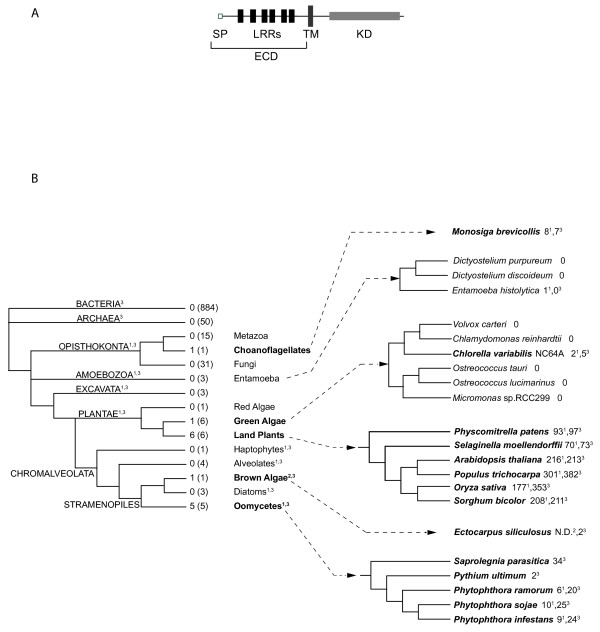
**Features and number of LRR-containing receptors in the analyzed genomes**. **(A) **Schematic representation of LRR-RLK (land plants) and LRR-RK (other genomes) receptors. Each of these proteins contains a signal peptide (SP, empty box), 1 to 30 LRRs (black boxes) in their extracellular domain (ECD), a transmembrane domain (TM, dark gray box) and an intracytoplasmic kinase domain (KD, pale gray box). **(B) **Schematic phylogenetic representation of all of the genomes analyzed. The tree is based on [34] and on [35] for the green algae lineage. The number of genomes in which LRR-RKs or LRR-RLKs (land plants) were found is followed in parentheses by the total number of genomes analyzed for each kingdom. On the right, the number of LRR-RLKs or LRR-RKs is given following the name of each species. Among the green algae, 6 genomes were studied; only one (*Chlorella variabilis *NC64A) contains LRR-RK proteins. Among the oomycetes, 1 genome of Saprolegniales (*Saprolegnia parasitica*) and 4 genomes of Peronosporales (*Pythium ultimum*, *Phytophthora infestans*, *Phytophthora ramorum *and *Phytophthora sojae*) were analyzed. Only proteins with an LRR-TM-KD organization are considered to be LRR-RKs. ^1^Soanes et al. 2010, ^2^Cock et al. 2010, ^3^this study, N.D. Not Determined.

Receptor-like kinases (RLKs) are plant-specific transmembrane (TM) receptor kinases (RKs) that are closely related to the Pelle proteins, a family of animal cytoplasmic kinases. These RLK/Pelle proteins are involved in host defense against a range of pathogens and are also key regulators of many developmental processes in both plants and animals [1-4]. In an extended phylogenetic analysis of eukaryotic receptor kinases, Shiu and Bleecker (2001) have shown that, with respect to the kinase domain (KD), the RLK/Pelle, receptor tyrosine kinase (RTK), receptor serine/threonine kinase (RSK) and Raf protein subfamilies form a monophyletic group, the receptor kinase group (RKG), that is distinct from all other eukaryotic kinases [5,6]. All plant RLKs possess a single-pass TM domain and an intracytoplasmic KD but differ in their extracellular domain (ECD) [5]. Members of the largest RLK subfamily, the leucine-rich repeat receptor-like kinases (LRR-RLKs), contain 1 to 30 leucine-rich repeats (LRRs) in their ECDs (Figure 1A). Plant LRR-RLKs are involved in many developmental processes and in host responses to biotic and abiotic stresses [7,8]. Animals also possess LRR-containing receptors (the Toll and Toll-like receptors) that play a role in development and immunity [9]. Interestingly, these animal receptors contain LRRs in their ECD but do not possess a KD, and several transduce their signal by activating kinase-containing co-receptors through the binding to adaptor proteins. Because the structural organization (LRRs exclusively in the ECD, followed by a TM and a KD with serine/threonine kinase activity) was previously found only in plants, a plant-specific dogma for LRR-RLKs was expounded for many years. However, LRR and KD domains are highly versatile with respect to their associations with other protein domains and are predicted to be present in all genomes from bacteria to humans [10].

In this report, we show that LRR-KD subfamilies have been reinvented in several eukaryotic genomes outside plants. Moreover, the evolutionary history of these LRR-RKs is comparable to the one described for the LRR-RLK plant subfamilies.

## Results and discussion

### LRR-containing receptor kinases are not plant-specific

As LRRs and KDs are present in all genomes, we searched for the presence of structurally related LRR-RLKs in non-plant lineages. We analyzed 884 bacterial, 50 archaeal and 77 eukaryotic genomes to identify LRR-containing RKs that were structurally related to plant LRR-RLKs (Figure 1B). Additional file 1 details the references and links for all of the genomes analyzed. Our study reveals that, among all of the genomes outside of land plants that were analyzed, LRR receptor kinase (LRR-RK) subfamilies are present in the genomes of *Monosiga brevicollis *(a choanoflagellate), *Chlorella variabilis *NC64A (a green alga) and several stramenopiles (*Ectocarpus siliculosus *[a brown alga] and all of the oomycetes analyzed) (Figure 1B). Some of these findings have recently been reported, albeit unobtrusively, mentioned as only a side discovery notice in two articles [11,12]. Our detailed analysis shows that the *Ectocarpus*, *Chlorella *and *Monosiga *genomes contain 2, 5 and 7 LRR-RK genes, respectively, whereas the oomycete species (*Saprolegnia parasitica*, *Pythium ultimum*, *Phytophthora infestans*, *Phytophthora ramorum *and *Phytophthora sojae*) contain up to 34 LRR-RK genes per genome. The step-by-step procedure used to detect the LRR-RK genes is described in Additional file 2. These LRR-RKs possess up to 26 LRRs in their ECDs (Additional file 3). Interestingly, although we searched for other domains (known to be present in plant ECDs, including lectins, duf26, EGF, lysM, Slocus, thaumatin and PAN) associated with RKs in oomycetes, we did not find any.

### LRR-RK subfamilies have been reinvented several times in eukaryotic genomes and evolved independently from each other

**Figure 2:**
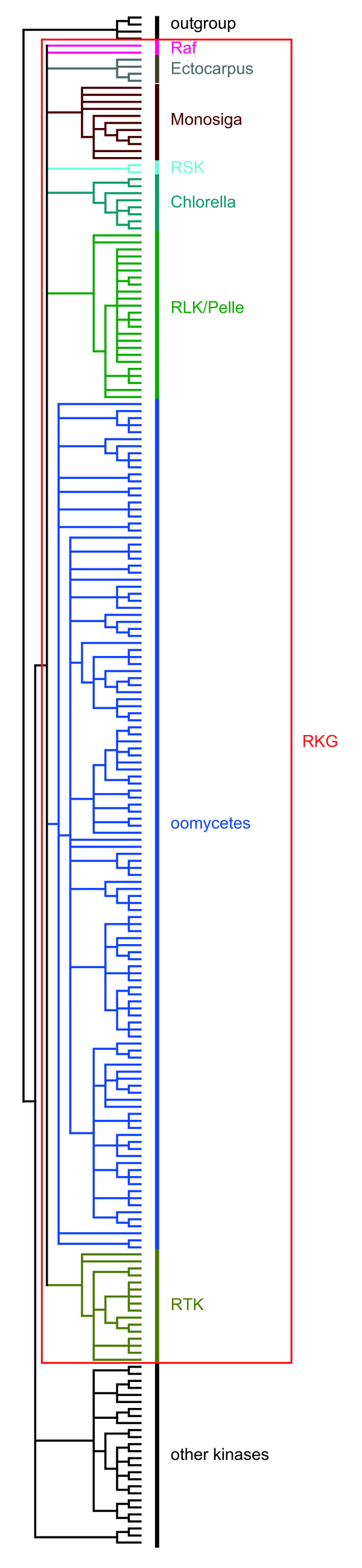
**Topology of the maximum likelihood tree representing the phylogenetic relationships among the LRR-RK KDs and other eukaryotic kinases**. The phylogenetic tree was generated from an alignment of the KDs (Additional file 4) of representative Arabidopsis and animal protein kinases, LRR-containing RKs and closely related receptors lacking TM domains and/or LRRs from *Monosiga brevicollis *(Monosiga, brown), *Chlorella variabilis *NC64A (Chlorella, greenish blue), *Ectocarpus siliculosus *(Ectocarpus, gray) and oomycetes (oomycetes, blue). Oomycete LRR-RKs, *Monosiga *LRR-RKs, *Chlorella *LRR-RKs and *Ectocarpus *LRR-RKs are included in the monophyletic receptor kinase group (RKG, red box), consisting of the plant receptor-like kinase (RLK) and animal cytoplasmic Pelle (forming the RLK/Pelle subfamily, green), receptor serine/threonine kinase (RSK, light blue), receptor tyrosine kinase (RTK, khaki) and Raf (Raf, pink) proteins. The RKG members are distinct from the other eukaryotic kinases. See Additional file 5 for the detailed phylogenetic tree.

A phylogenetic analysis of a representative subset of animal and plant eukaryotic kinases and all of the LRR-RKs, including receptors that are closely related to LRR-RKs but lack TM domains and/or LRRs, has been performed (Figure 2; see Additional file 4 for the sequence alignment and Additional file 5 for the details of Figure 2). This analysis reveals that the *Ectocarpus*, *Chlorella*, *Monosiga *and oomycete LRR-RK genes form four separate lineage-specific clades within the eukaryotic RKG. Because we did not observe incongruence between the phylogenetic and the species trees, we excluded the hypothesis of lateral transfer of the LRR-RK genes between species. However, because the tree is not fully resolved, we cannot rule out the possibility that the KDs of the LRR-RKs are more closely related to one of the RKG subfamilies. Nevertheless, with regard to the combination of the three modules (LRRs, TM and KD) to form new genes that are structurally related to LRR-RLKs, the most parsimonious hypothesis, based primarily on the species tree (Figure 1B), is that the association of LRRs with RKs appeared independently at least four times in eukaryotic evolutionary history. This hypothesis is supported by previous work on plants that showed that the KD of the RLK families has been recruited several times to form all of the LRR-RLK subfamilies [5]. Moreover, LRR domains are known to be highly versatile with respect to their associations with other protein domains [10]. Thus, our study expands what has been observed in plant lineages to several eukaryotic genomes. Alternatively, even if much less parsimonious, we cannot completely rule out a third hypothesis, which states that the LRR-RK and LRR-RLK gene families evolved from a common ancestor and have been subjected to massive gene losses.

### LRR-RK genes are in expansion in certain oomycete genomes

In plants, LRR-RLKs show a pattern of expansion with amplification/deletion and evolution of domain organization leading to the functional diversification of the members of the gene family [1]. The same evolutionary history can be observed in the oomycete LRR-RK subfamily. Indeed, we were able to identify gains and losses of LRR-RKs in each oomycete genome analyzed (Additional file 6). Moreover, some of the gene subgroups are *Saprolegnia*- or *Phytophthora*-specific, suggesting that several duplication events occurred independently in the *Saprolegnia *and *Phytophthora *genomes to give rise to ~25 copies in each lineage (Additional file 5B). In the *Phytophthora*-specific subgroups, most of the duplications are present in all of the *Phytophthora *genomes, indicating that these genetic changes occurred in the last common ancestor of the *Phytophthora *species. However, a few duplications are species specific, implying the possibility for the acquisition of new functions of these genes in these species. Notably, the absence of amplification in the *Pythium *genome and the independent amplifications in the *Saprolegnia *and *Phytophthora *genomes suggest that these genes may be involved in signaling pathways and, therefore, in functions that have diverged between *Saprolegnia*, *Phytophthora *and *Pythium *species. Indeed, similar to what is known in plants, these oomycete receptors could be involved in the perception of diverse signals leading to multiple cellular responses [8,13,14].

### Oomycete LRR-RKs may play a role in several stages of the oomycete life cycle

**Figure 3:**
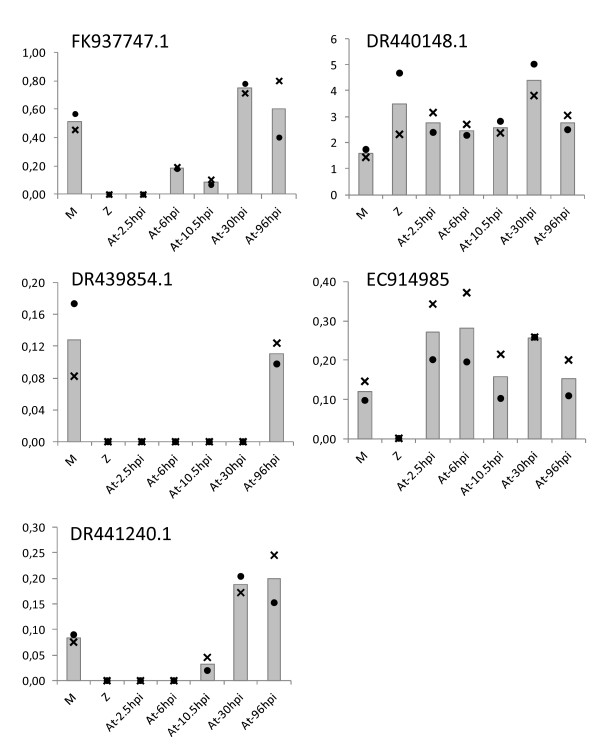
**Expression analysis of 5 *Phytophthora parasitica *LRR-RK genes during their interaction with *Arabidopsis thaliana***. The relative mRNA levels have been quantified by qRT-PCR at different development stages: samples corresponding to *Phytophthora parasitica *mycelium grown in V8 medium (M), *Phytophthora parasitica *motile zoospores (Z), and *Arabidopsis thaliana *roots collected 2.5 (At-2.5 hpi), 6 (At-6 hpi), 10.5 (At-10.5 hpi), 30 (At-30 hpi) and 96 (At-96 hpi) hours after inoculation with *Phytophthora parasitica *zoospores [30]. The data are presented as expression ratios relative to the mean expression values of three reference genes (2^-DCT^). Two independent RNA extractions corresponding to a pool of more than 5 biological replicates each were used. • and ×, biological replicates; bars, mean values.

To determine whether these oomycete LRR-RK genes are expressed, we first performed an *in silico *gene expression analysis by identifying expressed sequenced tags (ESTs) in the public databases (Additional file 7). Among the 85 oomycete LRR-RK genes assessed, 22 are represented by one to eight ESTs. These ESTs were obtained from libraries of various developmental stages, indicating that the oomycete LRR-RK genes are expressed during vegetative growth, mating, dissemination and host infection. To analyze the expression of these LRR-RK genes further, we used the plant pathosystem available in our laboratory: the *Phytophthora parasitica*/*Arabidopsis thaliana *interaction [15]. First, we searched for ESTs of *Phytophthora parasitica *and retrieved 6 ESTs (Additional file 8). Next, we verified and analyzed the expression patterns of these LRR-RK genes using quantitative reverse-transcriptase polymerase chain reaction (qRT-PCR) at several key stages during the course of the pathogen/host interaction (Figure 3): penetration (At-2.5 hpi (hours post inoculation)), biotrophic invasive growth (At-6 hpi and At-10.5 hpi), switch to necrotrophy (At-30 hpi) and necrotrophy (At-96 hpi). The data reveal that these genes are actually differentially expressed in the course of *Arabidopsis thaliana *infection. Moreover, the divergences in the expression profiles among the LRR-RK genes analyzed indicate that functional diversification may have occurred in the oomycete LRR-RK family. Taken together, these results suggest that these receptors could be involved in the perception of environmental cues, in the adaptation to specific conditions encountered in the host and/or in various developmental processes.

## Conclusions

In conclusion, we have shown that the *Monosiga*, *Chlorella*, *Ectocarpus *and oomycete LRR-RK receptors belong to the RKG and are likely to have acquired the LRRs in their ECDs independently. The evolutionary history of the oomycete LRR-RK receptor subfamily is consistent with the molecular evolution of plant LRR-RLKs [16,17]. *Saprolegnia *and *Phytophthora *species have developed, expanded and functionally diversified a subfamily of receptors that are structurally, but not phylogenetically, related to plant LRR-RLKs. Considering the key roles that plant LRR-RLKs play throughout the plant life cycle and in plant-environment interactions, it is tempting to propose that oomycete LRR-RKs may be important regulators of the oomycete life cycle. Future work should focus on deciphering the functions of oomycete LRR-RKs in host-oomycete interactions to reveal new targets to help combat these pathogens, which pose a serious threat to plants and aquaculture farming worldwide, causing tremendous economic damage every year [18].

## Methods

### Genomes analyzed

To analyze the representative species across all kingdoms, we first downloaded several publicly available completely sequenced genomes from each of the major kingdoms (Opisthokonta, Plantae, and Chromalveolates) from 2 sources (JGI and NCBI). We also downloaded all of the available bacterial and archaeal genomes from NCBI. When LRR-RK sequences were found in one genome, we downloaded all of the available complete genomes in that phylum. Thus, the complete proteomes of 77 eukaryotic, 50 archaeal and 884 bacterial species have been downloaded from their respective databases. See Additional file 1 for details regarding the genomes analyzed.

### Sequence retrieval and domain predictions

We retrieved genes containing leucine-rich repeats (LRRs) and a kinase domain (KD) by running the hmmsearch program (HMMER 2.3.2) to search for the kinase Hidden Markov Model (HMM) profile (PF00069.16) within the proteomic sequences of completely sequenced genomes. Within this set of kinase proteins, we then searched for LRR-domain HMM profiles (PF00560.24) (E value cut-off < 1) [19,20]. Signal peptides (SPs) and transmembrane domains (TMs) were predicted using the SignalP http://www.cbs.dtu.dk/services/SignalP/ and TMHMM http://www.cbs.dtu.dk/services/TMHMM/ websites, respectively, hosted at the Center for Biological Sequence Analysis, Technical University of Denmark [21]. Proteins containing LRRs, a TM and a KD were then considered to be putative LRR-RKs. We used the SMART web site http://smart.embl-heidelberg.de/ to check whether domains other than LRRs were predicted in the extracellular domain (ECD) of each protein [22]. If other domains were detected, the protein was rejected. Proteins containing only LRRs in their extracellular domain (ECD), a TM domain and a KD were classified as LRR-RKs. For the phylogenetic analysis presented in Figure 2, we first retrieved all of the peptide sequences of eukaryotic protein kinases used in [5] to establish the phylogenetic relationship between plant and animal protein kinases. We next retrieved one LRR-RLK protein per subgroup from the Arabidopsis genome. Finally, we included all of the newly identified LRR-RK proteins from the oomycete, *Monosiga*, *Ectocarpus *and *Chlorella *genomes. In oomycetes, *Ectocarpus*, *Monosiga *and *Chlorella*, we also retrieved proteins sequences of closely related kinases found using a Blastp search. This search was performed using the KD of the LRR-RKs, and we selected the non-LRR-RK best hit. Accessions numbers of all of these sequences are listed in the 'Accession number' section below.

### Alignment and phylogenetic analysis

Peptide KD sequences of all of the kinases to be analyzed (plus four bacterial kinase genes [YP_003956736.1, ZP_04777056.1, ZP_06621294.1 and P0A5S4.1] used as the outgroup [23]) were aligned using the MAFFT program (v6.525 b, einsi parameters, 1000 iterations maximum) and manually curated [24]. Phylogenetic trees were generated under the maximum likelihood criterion using PhyML 3.0 (LG model, NNI topological moves, optimizing branch lengths and branch supports). For the approximate likelihood ratio test (aLRT), we used the minimum value between the parametric approximate likelihood ratio test (aLRT, Chi2-based) and the non-parametric aLRT (based on a Shimodaira-Hasegawa-like procedure) [25,26]. All of the branches with support values less than 90 were collapsed. All of the manipulations of phylogenetic trees were performed using the TreeDyn [27] and MEGA4 [28] programs.

### Expression analysis

We used the NCBI tBLASTn web interface to search for expressed ESTs that were similar (identity > 95%) to our set of oomycete LRR-RK proteins [29]. Only the *Phytophthora infestans, Phytophthora sojae, Pythium ultimum *and *Saprolegnia parasitica *EST databases have been searched, as the EST database of the *Phytophthora ramorum *genome was not available. Each EST sequence retrieved was validated by a BLASTn search using the library of nucleotide sequences from that species and from the *Phytophthora infestans *nucleotide sequences in GenBank. To search for ESTs from *Phytophthora parasitica*, the 24 LRR-RK peptide sequences of *Phytophthora infestans *have been used as query for a tBLASTn search on the VBI microbial database http://vmd.vbi.vt.edu/toolkit/index.php. We used the *Phytophthora infestans *LRR-RK proteins as queries because it is the most complete dataset thus far. This search revealed that at least 25 *Phytophthora parasitica *genes are homologous to the 24 *Phytophthora infestans *LRR-RK genes. We searched for ESTs for each of these 25 *Phytophthora parasitica *LRR-RKs on the NCBI *Phytophthora parasitica *EST database. The qRT-PCR expression analysis of 5 of the 6 *Phytophthora parasitica *ESTs retrieved was performed as described in Kebdani et al. (2010) using UBC, WS21 and Mago nashi protein encoding sequences used as reference genes [30-32]. Note that one of the 6 ESTs (DR440392.1) has not been analyzed by qRT-PCR because we did not succeed in designing oligonucleotides sets for this gene.

### Accession numbers

The accession numbers for *Arabidopsis thaliana *are as follows: AtCKI1 [GenBank, CAA55395]; AtCDC2a [GenBank, AAB23643]; AtCPK7 [GenBank, AAB03247]; AtCKA1 [GenBank, BAA01090]; AtCTR1_Raf [GenBank, AAA32779]; AtAME2 [GenBank, BAA08215]; AtMKK3 [GenBank, BAA28829]; AtMEKK1 [GenBank, BAA09057]; AtNAK [GenBank, AAA18853]; AtNPH1 [GenBank, AAC01753]; AtPVPKlikePK5 [GenBank, BAA01715]; AtGSK3b [GenBank, CAA64408]; AtGSK3i [GenBank, CAA68027]; AtSnRK2 [GenBank, AAA32845]; AtMPK1 [TAIR, AT1G10210]; AtS6KlikePK1 [GenBank, AAA21142] and AtTousled [GenBank, AAA32874]. The accession numbers for *Homo sapiens *are as follows: hAXL [GenBank, NP_001690]; hRYK [GenBank, P34925]; hTRKalpha [GenBank, BAA34355]; hMuSK [GenBank, AAB63044]; hKLGlikePTK7 [GenBank, AAC50484]; hIR [GenBank, NP_000199]; hLTK [GenBank, P29376.3]; hRET [GenBank, AAH04257]; hTIE1 [GenBank, P35590]; hPDGFRbeta [GenBank, NM_002600.1]; hVGFR1 [GenBank, P17948.2]; hTousledLK1 [GenBank, NP_036422]; hMAPKKK1 [GenBank, Q13233]; hCLK1 [GenBank, P49759]; hMAPK1 [GenBank, NP_002736.3]; hCDK3 [GenBank, NP_001249]; hCKIalpha2 [GenBank, NP_001883]; hCaMK1 [GenBank, BAG70221]; hCK2a [GenBank, CAB65624]; hGRK6 [GenBank, P43250]; hEGFR [GenBank, P00533]; hFGFR2 [GenBank, P21802]; hHGFR [GenBank, P08581]; hEPH [GenBank, P21709]; hDDR [GenBank, Q08345]; hRaf1 [GenBank, AAA60247]; TGF beta receptors, hTGFbRI [GenBank, P36897] and hTGFbRII [GenBank, P37173]; hIRAK1 [GenBank, AAH54000] and hMAPKK1 [GenBank, Q02750]. Additional accession numbers are as follows: mCKIalpha [GenBank, NP_666199] for *Mus musculus*; xtPELLE [GenBank, NP_001006713] for *Xenopus tropicalis*; drIRAK1 [GenBank, CAP19555] for *Danio rerio *and dmPELLE [GenBank, NP_476971] for *Drosophila melanogaster*. The accessions of representative *Arabidopsis thaliana *LRR-RLK genes in each subfamily are as follows: [TAIR: AT4G29180] for LRRI, AtNIK1 for LRRII [TAIR: AT5G16000], AtIMK3 for LRRIII [TAIR: AT3G56100], [TAIR: AT2G45340] for LRRIV, AtSCM_SUB for LRRV [TAIR: AT1G11130], [TAIR: AT1G14390] for LRRVI-1, [TAIR: AT5G41180] for LRRVI-2, [TAIR: AT2G24230] for LRRVII, [TAIR: AT1G06840] for LRRVIII-1, [TAIR: AT3G14840] for LRRVIII-2, AtTMK1 for LRRIX [TAIR: AT1G66150], [TAIR: AT3G28450] for LRRXa, AtBRI1 for LRRXb [TAIR: AT4G39400), AtCLV1 for LRRXI [TAIR: AT1G75820], AtFLS2 for LRRXII [TAIR: AT5G46330], AtFEI1 for LRRXIIIa [TAIR: AT1G31420], AtER for LRRXIIIb [TAIR: AT2G26330], [TAIR: AT3G14840] for LRRXIV and AtRPK1 for LRRXV [TAIR: AT1G69270]. We followed the subfamily nomenclature of a previous report [33].

## List of abbreviations

LRR: leucine-rich repeat; RLK: receptor-like kinases; RK: receptor kinase; LRR-RLK: leucine-rich repeat receptor-like kinase; LRR-RK: leucine-rich repeat receptor kinase; TM: transmembrane; KD: kinase domain; ECD: extracellular domain; RKG: receptor kinase group; RTK: receptor tyrosine kinase; RSK: receptor serine/threonine kinase; EST: expressed sequence tag; TM: transmembrane domain; SP: signal peptide; hpi: hours post inoculation.

## Competing interests

The authors declare that they have no competing interests.

## Authors' contributions

AD, NG and CP designed the experiments and discussed the results. AD and GD performed the bioinformatics analyses. AA and MG performed the qRT-PCR experiments. AD, NG, EG and CP drafted the manuscript. All of the authors have read and approved the final manuscript.

## Supplementary Material

Additional file 1 **Genomes analyzed**. Click here for file

Additional file 2 **Step-by-step procedure to determine the number of LRR-RKs per genome**. The last column of the table shows the number of proteins that we considered to be LRR-RKs in our analysis. The accession number of each gene is listed in Additional file 3. KD, kinase domain; LRRs, leucine-rich repeats; TM, transmembrane domain; ECD, extracellular domain; LRR-RKs, Leucine-rich repeat receptor kinase. Click here for file

Additional file 3 **Structural features of the oomycete, *Monosiga*, *Chlorella *and *Ectocarpus *LRR-RKs and closely related receptors lacking LRRs and/or TM domains**. All sequences except one sequence of *Phytophthora ramorum *(Pr81779) and one sequence of *Phytophthora sojae *(Ps136026) have been used for the phylogenetic analysis presented in Figure 2 and Additional file 5. These sequences were excluded because their KDs did not align with those of the other kinases. In addition, one sequence of *Monosiga brevicollis *(Mb34608) and one sequence of *Chlorella variabilis *NC64A (Ch136834) produced inconsistent results in the phylogenetic analysis and do not appear in the tree. These sequences may represent misannotated proteins or pseudogenes. Abbreviations: S, *Saprolegnia parasitica*; Pi, *Phytophthora infestans*; Pr, *Phytophthora ramorum*; Ps, *Phytophthora sojae*; Pu, *Pythium ultimum*; Mb, *Monosiga brevicollis*; Ch, *Chlorella variabilis *NC64A; Esi, *Ectocarpus siliculosus*; SP, signal peptide; KD, kinase domain; LRRs, leucine-rich repeats; +, presence; -, absence. See Materials and Methods for details about predicted SPs, LRR numbers, TM domains and KDs. Click here for file

Additional file 4 **Alignment of LRR-RKs and reference eukaryotic KDs**. The KDs of LRR-RKs from *Ectocarpus siliculosus *(Esi), *Phytophthora ramorum *(Pr), *Phytophthora sojae *(Ps), *Phytophthora infestans *(Pi), *Saprolegnia parasitica *(S), *Pythium ultimum *(Pu), *Chlorella variabilis *NC64A (Ch) and *Monosiga brevicollis *(Mb) were aligned with reference eukaryotic kinases from human (h), *Arabidopsis thaliana *(At), *Drosophila melanogaster *(dm), *Danio rerio *(dr), mouse (m) and *Xenopus tropicalis *(Xt). The positions of the 12 kinase subdomains are shown in roman numerals. Click here for file

Additional file 5 **Details of the maximum likelihood tree representing the phylogenetic relationships among the LRR-RK KDs and other eukaryotic kinases**. **A**. **General view of the tree presented in **Figure 2. The phylogenetic tree was generated from an alignment of the KDs (Additional file 4) of representative Arabidopsis and animal protein kinases, LRR-containing RKs (☼) and closely related receptors lacking TM domains and/or LRRs. These sequences, devoid of LRRs and/or TM domains, have been included to highlight the versatility of these domains with respect to their associations with KDs. The tree branches are colored as follows: *Monosiga brevicollis *(Monosiga, brown); *Chlorella variabilis *NC64A (Chlorella, greenish blue); *Ectocarpus siliculosus *(Ectocarpus, gray) and oomycetes (oomycetes, blue). Oomycete LRR-RKs, *Monosiga *LRR-RKs, *Chlorella *LRR-RKs and *Ectocarpus *LRR-RKs are included in the monophyletic receptor kinase group (RKG, red box) consisting of the plant receptor-like kinase (RLK) and animal cytoplasmic Pelle (forming the RLK/Pelle subfamily, green), receptor serine/threonine kinase (RSK, light blue), receptor tyrosine kinase (RTK, khaki) and Raf (Raf, pink) proteins. The RKG members are distinct from other the eukaryotic kinases. Note that the tree is not fully resolved. The addition of more RKs lacking LRRs to this phylogenetic analysis did not improve the resolution of the tree (data not shown); similarly, neither did the addition of non-RKs from the oomycete species (data not shown). Branch support values are shown at the nodes. **B. A detailed view of the oomycete clade**. In the oomycete LRR-RK subfamily, some subdivisions are *Saprolegnia *or *Phytophthora *specific (black boxes), suggesting the lineage-specific amplification by duplications in the *Saprolegnia *and *Phytophthora *genomes. Three subgroups contain both of these lineages with or without *Pythium *(orange boxes), suggesting that at least three genes were present in the last common ancestor of these species. Oomycete genes with evidence of expression (expressed sequence tags) are indicated with a plus sign (details in Additional file 7). **C. A detailed view of other clades**. Click here for file

Additional file 6 **Number of gains and losses of oomycete LRR-RK genes in orthologous groups**. We counted the number of genes gained and lost for each orthologous group defined in the oomycete clades of the phylogenetic tree (Additional file 5B). **A**. Orthologous groups containing *Saprolegnia*, *Pythium *and *Phytophthora *species, **B**. orthologous groups containing only *Pythium *and *Phytophthora *species and **C**. orthologous groups containing only *Phytophthora *species. The number on the first branch represents the number of genes present in the last common ancestor. Numbers preceded by a plus sign are the number of genes gained; numbers preceded by a minus sign are the number of genes lost. Click here for file

Additional file 7 **Oomycete LRR-RK subgroups with evidence of expression**. Abbreviations: S, *Saprolegnia parasitica*; Pi, *Phytophthora infestans*; Ps, *Phytophthora sojae*; Pp, *Phytophthora parasitica*; Pp, *Phytophthora parasitica*; EST, expressed sequence tags. Click here for file

Additional file 8 ***Phytophthora parasitica *LRR-RKs with evidence of expression**. Abbreviations: Pi, *Phytophthora infestans*; EST, expressed sequence tags. Click here for file
