# Supplementary material for: Leucine-Rich repeat receptor kinases are sporadically distributed in eukaryotic genomes
Source: BMC Evol Biol. 2011 Dec 20;11:367. doi: 10.1186/1471-2148-11-367 (PMC3268121; doi:10.1186/1471-2148-11-367)
Supplement: Additional file 1 — Genomes analyzed. [file 1471-2148-11-367-S1.DOC]

**Additional file 1: Genomes analyzed**

**46 OPISTHOKONTA GENOMES**:

**1 Choanoflagellates**: *Monosiga brevicollis* (ftp://ftp.jgi-psf.org/pub/JGI_data/Monosiga_brevicollis/annotation/v1.0/Monbr1_best_proteins.fasta.gz) [1];

**15 Metazoa**: *Lottia gigantea* (http://genome.jgi-psf.org/Lotgi1/Lotgi1.download.ftp.html); *Acyrthosiphon pisum* (ftp://ftp.ncbi.nih.gov/genomes/Acyrthosiphon_pisum/protein/) [2]; *Drosophila melanogaster* (ftp://ftp.flybase.net/genomes/Drosophila_melanogaster/dmel_r5.10_FB2008_07/fasta/) [3]; *Daphnia pulex* (http://genome.jgi-psf.org/Dappu1/download/FilteredModelsv1.0.aa.fasta.gz); *Helobdella robusta* (ftp://ftp.jgi-psf.org/pub/JGI_data/Helobdella_robusta/v1.0/proteins.Helro1_FilteredModels3.fasta.gz); *Danio rerio* (ftp://ftp.ncbi.nih.gov/genomes/D_rerio/protein/, V2.1); *Xenopus* *tropicalis* (ftp://ftp.jgi-psf.org/pub/JGI_data/Frog/v4.1/proteins.Xentr4.fasta.gz); *Takifugu rubripes* (ftp://ftp.jgi-psf.org/pub/JGI_data/Fugu/v4.0/proteins.Takru4.fasta.gz); *Branchiostoma floridae* (ftp://ftp.jgi-psf.org/pub/JGI_data/Branchiostoma_floridae/v1.0/proteins.Brafl1.fasta.gz) [4]; *Ciona intestinalis* (ftp://ftp.jgi-psf.org/pub/JGI_data/Ciona/v2.0/FM1.aa.fasta.gz) [5]; *Homo sapiens* (ftp://ftp.ncbi.nih.gov/genomes/H_sapiens/protein/Build 16.1, 2009-08-03) [6]; *Bos taurus* (ftp://ftp.ncbi.nih.gov/genomes/Bos_taurus/ARCHIVE/BUILD.4.1/protein/, 2008-08-05) [7]; *Gallus gallus* (ftp://ftp.ncbi.nih.gov/genomes/Gallus_gallus/protein/) [8]; *Mus musculus* (ftp://ftp.ncbi.nih.gov/genomes/M_musculus/protein/) [9]; *Nematostella vectensis* (ftp://ftp.jgi-psf.org/pub/JGI_data/Nematostella_vectensis/v1.0/annotation/proteins.Nemve1FilteredModels1.fasta.gz) [10]; *Trichoplax adhaerens* (ftp://ftp.jgi-psf.org/pub/JGI_data/Trichoplax_adhaerens_Grell-BS-1999/annotation/v1.0/Triad1_best_proteins.fasta.gz) [11];

**31 fungi**: *Pyrenophora tritici-repentis* (http://www.broadinstitute.org/annotation/genome/pyrenophora_tritici_repentis.3/MultiDownloads.html); *Stagonospora nodorum* (http://genome.jgi-psf.org/Stano1/download/Stagonospora_nodorum_SN15_proteins.fasta.gz) [12]; *Aspergillus oryzae* (http://www.bio.nite.go.jp/dogan/) [13]; *Aspergillus fumigatus* (ftp://ftp.ncbi.nih.gov/genomes/Fungi/Aspergillus_fumigatus/) [14]; *Aspergillus nidulans* (ftp://ftp.ncbi.nih.gov/genomes/Fungi/Aspergillus_nidulans_FGSC_A4/) [15]; *Microsporum gypseum* (http://www.broadinstitute.org/annotation/genome/dermatophyte_comparative/MultiDownloads.html); *Paracoccidioides brasiliensis* (http://www.broadinstitute.org/annotation/genome/paracoccidioides_brasiliensis/MultiDownloads.html); *Trichophyton tonsurans* (http://www.broadinstitute.org/annotation/genome/dermatophyte_comparative/MultiDownloads.html); *Botrytis cinerea* (http://www.broadinstitute.org/annotation/genome/botrytis_cinerea.2/MultiDownloads.html); *Fusarium graminearum* (http://www.broadinstitute.org/annotation/genome/fusarium_graminearum/MultiDownloads.html) [16]; *Gibberella zeae* (ftp://ftp.ncbi.nih.gov/genomes/Fungi/Gibberella_zeae_PH-1/); *Thielavia terrestris* (http://genome.jgi-psf.org/Thite1/download/Thite1_GeneModels_FilteredModels2_aa.fasta.gz); *Verticillium dahliae* (http://www.broadinstitute.org/annotation/genome/verticillium_dahliae/MultiDownloads.htm); *Candida albicans* (ftp://ftp.ncbi.nih.gov/genomes/Fungi/Candida_albicans/) [17]; *Candida glabrata* (ftp://ftp.ncbi.nih.gov/genomes/Fungi/Candida_glabrata_CBS138/) [18]; *Debaryomyces hansenii* (ftp://ftp.ncbi.nih.gov/genomes/Fungi/Debaryomyces_hansenii_CBS767/) [19]; *Eremothecium gossypii* (ftp://ftp.ncbi.nih.gov/genomes/Fungi/Eremothecium_gossypii/) [20]; *Kluyveromyces lactis* (ftp://ftp.ncbi.nih.gov/genomes/Fungi/Kluyveromyces_lactis_NRRL_Y-1140/) [18]; *Pichia stipitis* (ftp://ftp.ncbi.nih.gov/genomes/Fungi/Pichia_stipitis_CBS_6054/) [21]; *Saccharomyces cerevisiae* (http://www.broadinstitute.org/annotation/genome/saccharomyces_cerevisiae.3/MultiDownloads.html, RM11-1a, 2009-06) [22]; *Yarrowia lipolytica* (ftp://ftp.ncbi.nih.gov/genomes/Fungi/Yarrowia_lipolytica_CLIB122/) [18]; *Sclerotinia sclerotiorum* (http://www.broadinstitute.org/annotation/genome/sclerotinia_sclerotiorum/MultiDownloads.html); *Podospora anserina* (http://podospora.igmors.u-psud.fr/download.php) [23]; *Neurospora crassa* (http://www.broadinstitute.org/annotation/genome/neurospora/MultiDownloads.html) [24]; *Magnaporthe grisea* (http://www.broadinstitute.org/annotation/genome/magnaporthe_grisea/MultiDownloads.html, 70-15, 2009-07) [25]; *Schizosaccharomyces pombe* (ftp://ftp.ncbi.nih.gov/genomes/Fungi/Schizosaccharomyces_pombe/) [26]; *Cryptococcus neoformans* (ftp://ftp.ncbi.nih.gov/genomes/Fungi/Cryptococcus_neoformans_var_JEC21/) [27]; *Puccinia graminis* f. sp. tritici (http://www.broadinstitute.org/annotation/genome/puccinia_group/MultiDownloads.html); *Ustilago maydis* (http://www.broadinstitute.org/annotation/genome/ustilago_maydis.2/MultiDownloads.html) [28]; *Encephalitozoon cuniculi* (ftp://ftp.ncbi.nih.gov/genomes/Fungi/Encephalitozoon_cuniculi/) [29] and *Rhizopus oryzae* (http://www.broadinstitute.org/annotation/genome/rhizopus_oryzae/MultiDownloads.html) [30];

**3 AMOEBOZOA GENOMES**: *Dictostelium discoideum* (http://dictybase.org/db/cgi-bin/dictyBase/download/download.pl?area=blast_databases&ID=dicty_primary_protein.gz, 2007-7-25) [31]; *Dictyostelium purpureum* (ftp://ftp.jgi-psf.org/pub/JGI_data/Dictyostelium_purpureum/v1.0/annotation/Dicpu1_best_proteins.fasta.gz) and *Entamoeba histolytica* (http://amoebadb.org/common/downloads/release-1.3/Ehistolytica/fasta/)[32];

**3 EXCAVATA GENOMES**: *Leishmania infantum* (ftp://ftp.ncbi.nih.gov/genomes/Protozoa/Leishmania_infantum/,2007-07-25) [33]; *Trypanosoma brucei* (ftp://ftp.ncbi.nih.gov/genomes/Protozoa/Trypanosoma_brucei/) [34] and *Naegleria gruberi* (http://genome.jgi-psf.org/Naegr1/download/Naegr1_best_proteins.fasta.gz) [35];

**10 PLANTAE GENOMES**: *Cyanidioschyzon merolae* (http://merolae.biol.s.u-tokyo.ac.jp/download/,2005-09-17) [36]; *Micromonas* sp.RCC299 (http://genome.jgi-psf.org/MicpuN3/download/MicromonasRCC299v3.FrozenGeneCatalog_20090404.proteins.fasta.gz) [37]; *Chlamydomonas reinhardtii* (http://genome.jgi-psf.org/Chlre4/download/annotation/Chlre4_best_proteins.fasta.gz) [38]; *Ostreococcus tauri* (ftp://ftp.jgi-psf.org/pub/JGI_data/Ostreococcus_tauri/O.tauri.FM.aa.fasta.gz) [39]; *Ostreococcus lucimarinus* (ftp://ftp.jgi-psf.org/pub/JGI_data/Ostreococcus_lucimarinus/O.lucimarinus.FM.aa.fasta.gz) [40]; *Chlorella* *variabilis* NC64A (http://genome.jgi-psf.org/ChlNC64A_1/ChlNC64A_1.download.ftp.html) [41]; *Volvox carteri* (http://genome.jgi-psf.org/Volca1/Volca1.download.ftp.html) [42]; *Physcomitrella patens* subsp. *patens* (ftp://ftp.jgi-psf.org/pub/JGI_data/Physcomitrella_patens/v1.1/proteins.Phypa1_1.FilteredModels.fasta.gz) [43]; *Arabidopsis thaliana* (ftp://ftp.arabidopsis.org/home/tair/Sequences/blast_datasets/TAIR9_blastsets/, 2009-06-19) [44] and *Oryza sativa* (ftp://ftp.plantbiology.msu.edu/pub/data/Eukaryotic_Projects/o_sativa/annotation_dbs/pseudomolecules/version_5.0) [45];

**17 CHROMALVEOLATA GENOMES**:

**1 Haptophytes**: *Emiliania huxleyi* CCMP1516 (http://genome.jgi-psf.org/Emihu1/download/annotation/v1.0/Emihu1_reduced_proteins.fasta.gz, CCMP1516);

**4 Alveolates**: *Paramecium tetraurelia* (http://paramecium.cgm.cnrs-gif.fr/download/fasta/archive/peptides/Ptetraurelia_peptides_v1.44.fasta.gz) [46]; *Plasmodium falciparum* (ftp://ftp.ncbi.nih.gov/genomes/Protozoa/Plasmodium_falciparum/, 3D7, 2007-11-26) [47]; *Cryptosporidium parvum* (ftp://ftp.ncbi.nih.gov/genomes/Protozoa/Cryptosporidium_parvum/) [48] and *Theileria parva* (ftp://ftp.ncbi.nih.gov/genomes/Protozoa/Theileria_parva/) [49];

**9 Stramenopiles**: 1 Brown algae: *Ectocarpus siliculosus* (https://bioinformatics.psb.ugent.be/gdb/ectocarpus/Ectsi_prot_LATEST.tfa.gz, 2010-06-16) [50]; 3 Diatoms: *Aureococcus anophagefferens* (ftp://ftp.jgi-psf.org/pub/JGI_data/Aureococcus_anophagefferens/annotation/v1.0/proteins.Auran1_FilteredModels3.fasta.gz); *Thalassiosira pseudonana* (http://genome.jgi-psf.org/Thaps3/download/Thaps3_chromosomes_geneModels_FilteredModels2_aa.fasta.gz) [51]; *Phaeodactylum tricornutum* (http://genome.jgi-psf.org/Phatr2/download/Phatr2_chromosomes_geneModels_FilteredModels2_aa.fasta.gz) [52]; 5 Oomycetes: *Saprolegnia parasitica* (http://www.broadinstitute.org/annotation/genome/Saprolegnia_parasitica/MultiDownloads.html, CBS 223.65, 2010-05); *Pythium ultimum* (http://pythium.plantbiology.msu.edu/data/pythium_ultimum_proteins.fasta.zip, BR144, 2010-08) [53]; *Phytophthora infestans* (http://www.broadinstitute.org/annotation/genome/phytophthora_infestans/MultiDownloads.html, T30-4, 2010-05) [54]; *Phytophthora ramorum* (ftp://ftp.jgi-psf.org/pub/JGI_data/Pramorum/assembly/v1.1/proteins.FM_Phyra1_1.fasta.gz) [55] and P*hytophthora sojae* (ftp://ftp.jgi-psf.org/pub/JGI_data/Psojae/assembly/v1.1/proteins.FM_Physo1_1.fasta.gz) [55].

**50 ARCHAEAL AND 884 BACTERIAL GENOMES** (ftp://ftp.ncbi.nih.gov/genomes/Bacteria/).
