# Supplementary material for: Leucine-Rich repeat receptor kinases are sporadically distributed in eukaryotic genomes
Source: BMC Evol Biol. 2011 Dec 20;11:367. doi: 10.1186/1471-2148-11-367 (PMC3268121; doi:10.1186/1471-2148-11-367)
Supplement: Additional file 2 — Step-by-step procedure to determine the number of LRR-RKs per genome. The last column of the table shows the number of proteins that we considered to be LRR-RKs in our analysis. The accession number of each gene is listed in Additional file 3. KD, kinase domain; LRRs, leucine-rich repeats; TM, transmembrane domain; ECD, extracellular domain; LRR-RKs, Leucine-rich repeat receptor kinase. [file 1471-2148-11-367-S2.DOC]

**Additional file 2. Step-by-strep procedure to determine the number of LRR-RKs per genome.**

| **Species** | **Phylogenetic group** | **Number of proteins with LRRs and KD** | **Presence of TM** | **Other domain than LRRs in ECD (LDLa)*** | **Number of proteins classified as LRR-RKs in our study** |
| --- | --- | --- | --- | --- | --- |
| *Monosiga brevicollis* | Choanoflagellates | 15 | 11 | 4 (LDLa) | 7 |
| *Chlorella NC64A* | Chlorophytes | 22 | 5 | 0 | 5 |
| *Ectocarpus siliculosus* | Stramenopiles | 14 | 2 | 0 | 2 |
| *Saprolegnia parasitica* | Stramenopiles | 36 | 34 | 0 | 34 |
| *Pythium ultimum* | Stramenopiles | 4 | 2 | 0 | 2 |
| *Phytophthora ramorum* | Stramenopiles | 23 | 20 | 0 | 20 |
| *Phytophthora sojae* | Stramenopiles | 28 | 25 | 0 | 25 |
| *Phytophthora infestans* | Stramenopiles | 27 | 24 | 0 | 24 |

* LDLa:Low-density lipoprotein receptor domain class A
