# Supplementary material for: Leucine-Rich repeat receptor kinases are sporadically distributed in eukaryotic genomes
Source: BMC Evol Biol. 2011 Dec 20;11:367. doi: 10.1186/1471-2148-11-367 (PMC3268121; doi:10.1186/1471-2148-11-367)
Supplement: Additional file 3 — Structural features of the oomycete, Monosiga, Chlorella and Ectocarpus LRR-RKs and closely related receptors lacking LRRs and/or TM domains. All sequences except one sequence of Phytophthora ramorum (Pr81779) and one sequence of Phytophthora sojae (Ps136026) have been used for the phylogenetic analysis presented in Figure 2 and Additional file 5. These sequences were excluded because their KDs did not align with those of the other kinases. In addition, one sequence of Monosiga brevicollis (Mb34608) and one sequence of Chlorella variabilis NC64A (Ch136834) produced inconsistent results in the phylogenetic analysis and do not appear in the tree. These sequences may represent misannotated proteins or pseudogenes. Abbreviations: S, Saprolegnia parasitica; Pi, Phytophthora infestans; Pr, Phytophthora ramorum; Ps, Phytophthora sojae; Pu, Pythium ultimum; Mb, Monosiga brevicollis; Ch, Chlorella variabilis NC64A; Esi, Ectocarpus siliculosus; SP, signal peptide; KD, kinase domain; LRRs, leucine-rich repeats; +, presence; -, absence. See Materials and Methods for details about predicted SPs, LRR numbers, TM domains and KDs. [file 1471-2148-11-367-S3.DOC]

**Additional file 3. Structural features of the oomycete, *Monosiga*, *Chlorella* and *Ectocarpus* LRR-RKs and closely related receptors lacking LRRs and/or TM domains.**

| Names | Accessions | Length | SP | Number of LRRs | TM domain | KD |
| --- | --- | --- | --- | --- | --- | --- |
| Pi00640T0 | PITG_00640T0 | 670 | - | 3 | + | + |
| Pi00643T0 | PITG_00643T0 | 896 | - | 3 | + | + |
| Pi00646T0 | PITG_00646T0 | 734 | + | 4 | + | + |
| Pi02124T0 | PITG_02124T0 | 737 | + | 4 | + | + |
| Pi03773T0 | PITG_03773T0 | 726 | + | 2 | + | + |
| Pi03775T0 | PITG_03775T0 | 696 | + | 3 | + | + |
| Pi04971T0 | PITG_04971T0 | 918 | - | 7 | + | + |
| Pi05112T0 | PITG_05112T0 | 699 | + | 4 | + | + |
| Pi05338T0 | PITG_05338T0 | 639 | + | 6 | + | + |
| Pi05340T0 | PITG_05340T0 | 722 | + | 3 | + | + |
| Pi07724T0 | PITG_07724T0 | 807 | + | 6 | + | + |
| Pi07725T0 | PITG_07725T0 | 774 | + | 4 | + | + |
| Pi07731T0 | PITG_07731T0 | 799 | + | 6 | + | + |
| Pi09664T0 | PITG_09664T0 | 760 | + | 6 | + | + |
| Pi09665T0 | PITG_09665T0 | 714 | + | 5 | + | + |
| Pi13397T0 | PITG_13397T0 | 747 | + | 6 | + | + |
| Pi14970T0 | PITG_14970T0 | 724 | + | 4 | + | + |
| Pi14971T0 | PITG_14971T0 | 814 | + | 3 | + | + |
| Pi14972T0 | PITG_14972T0 | 770 | + | 7 | + | + |
| Pi14992T0 | PITG_14992T0 | 937 | - | 9 | - | + |
| Pi17831T0 | PITG_17831T0 | 498 | - | 4 | - | + |
| Pi17832T0 | PITG_17832T0 | 767 | + | 4 | + | + |
| Pi17840T0 | PITG_17840T0 | 513 | - | 3 | + | + |
| Pi19256T0 | PITG_19256T0 | 296 | - | 0 | - | + |
| Pi21299T0 | PITG_21299T0 | 797 | + | 5 | + | + |
| Pi22892T0 | PITG_22892T0 | 728 | - | 2 | + | + |
| Pi23090T0 | PITG_23090T0 | 698 | + | 5 | - | + |
| Pi23143T0 | PITG_23143T0 | 542 | - | 3 | + | + |
| Pr72884 | jgi|Phyra1_1|72884|fgenesh1_pg.C_scaffold_1000326 | 751 | + | 3 | + | + |
| Pr72888 | jgi|Phyra1_1|72888|fgenesh1_pg.C_scaffold_1000330 | 1163 | - | 3 | + | + |
| Pr72890 | jgi|Phyra1_1|72890|fgenesh1_pg.C_scaffold_1000332 | 735 | + | 4 | + | + |
| Pr73421 | jgi|Phyra1_1|73421|fgenesh1_pg.C_scaffold_3000178 | 748 | + | 5 | + | + |
| Pr75310 | jgi|Phyra1_1|75310|fgenesh1_pg.C_scaffold_12000057 | 722 | + | 2 | + | + |
| Pr75312 | jgi|Phyra1_1|75312|fgenesh1_pg.C_scaffold_12000059 | 711 | + | 5 | + | + |
| Pr75691A* | jgi|Phyra1_1|75691|fgenesh1_pg.C_scaffold_14000053 | 1608 | + | 10 | + | + |
| Pr75692 | jgi|Phyra1_1|75692|fgenesh1_pg.C_scaffold_14000054 | 732 | + | 4 | + | + |
| Pr75791 | jgi|Phyra1_1|75791|fgenesh1_pg.C_scaffold_14000153 | 1072 | - | 5 | + | + |
| Pr75814 | jgi|Phyra1_1|75814|fgenesh1_pg.C_scaffold_14000176 | 733 | + | 5 | + | + |
| Pr76005 | jgi|Phyra1_1|76005|fgenesh1_pg.C_scaffold_15000139 | 703 | + | 0 | + | + |
| Pr76010 | jgi|Phyra1_1|76010|fgenesh1_pg.C_scaffold_15000144 | 647 | + | 2 | - | + |
| Pr77470 | jgi|Phyra1_1|77470|fgenesh1_pg.C_scaffold_24000139 | 806 | + | 5 | + | + |
| Pr77475 | jgi|Phyra1_1|77475|fgenesh1_pg.C_scaffold_24000144 | 1582 | + | 8 | + | + |
| Pr77476 | jgi|Phyra1_1|77476|fgenesh1_pg.C_scaffold_24000145 | 795 | + | 6 | + | + |
| Pr80778 | jgi|Phyra1_1|80778|fgenesh1_pg.C_scaffold_52000049 | 643 | - | 4 | + | + |
| Pr80780 | jgi|Phyra1_1|80780|fgenesh1_pg.C_scaffold_52000051 | 611 | + | 6 | + | + |
| Pr81229 | jgi|Phyra1_1|81229|fgenesh1_pg.C_scaffold_57000081 | 688 | + | 3 | + | + |
| Pr81472 | jgi|Phyra1_1|81472|fgenesh1_pg.C_scaffold_60000036 | 741 | + | 6 | + | + |
| Pr81631 | jgi|Phyra1_1|81631|fgenesh1_pg.C_scaffold_62000013 | 635 | + | 4 | + | + |
| Pr81779 | jgi|Phyra1_1|81779|fgenesh1_pg.C_scaffold_63000050 | 1048 | + | 3 | + | + |
| Pr82847 | jgi|Phyra1_1|82847|fgenesh1_pg.C_scaffold_78000042 | 697 | + | 4 | + | + |
| Pr93648 | jgi|Phyra1_1|93648|C_scaffold_4000012 | 1994 | + | 10 | - | + |
| Pr94275 | jgi|Phyra1_1|94275|C_scaffold_14000048 | 611 | - | 4 | - | + |
| Ps127796 | jgi|Physo1_1|127796|estExt_fgenesh1_pg.C_30005 | 687 | + | 4 | + | + |
| Ps132270 | jgi|Physo1_1|132270|estExt_fgenesh1_pg.C_200042 | 696 | + | 4 | + | + |
| Ps132640 | jgi|Physo1_1|132640|estExt_fgenesh1_pg.C_210200 | 738 | - | 2 | + | + |
| Ps132643 | jgi|Physo1_1|132643|estExt_fgenesh1_pg.C_210203 | 700 | - | 4 | + | + |
| Ps132644 | jgi|Physo1_1|132644|estExt_fgenesh1_pg.C_210204 | 706 | + | 2 | - | + |
| Ps133281 | jgi|Physo1_1|133281|estExt_fgenesh1_pg.C_250035 | 610 | + | 5 | + | + |
| Ps133283 | jgi|Physo1_1|133283|estExt_fgenesh1_pg.C_250037 | 687 | - | 2 | + | + |
| Ps133286 | jgi|Physo1_1|133286|estExt_fgenesh1_pg.C_250040 | 426 | + | 0 | - | + |
| Ps133943 | jgi|Physo1_1|133943|estExt_fgenesh1_pg.C_290010 | 747 | + | 2 | + | + |
| Ps135323 | jgi|Physo1_1|135323|estExt_fgenesh1_pg.C_370092 | 750 | - | 5 | + | + |
| Ps135332 | jgi|Physo1_1|135332|estExt_fgenesh1_pg.C_370101 | 592 | - | 2 | + | + |
| Ps135333 | jgi|Physo1_1|135333|estExt_fgenesh1_pg.C_370102 | 977 | + | 4 | + | + |
| Ps135334 | jgi|Physo1_1|135334|estExt_fgenesh1_pg.C_370103 | 752 | + | 5 | + | + |
| Ps136026 | jgi|Physo1_1|136026|estExt_fgenesh1_pg.C_420089 | 1099 | + | 2 | + | + |
| Ps136072 | jgi|Physo1_1|136072|estExt_fgenesh1_pg.C_430005 | 1028 | + | 8 | - | + |
| Ps136501 | jgi|Physo1_1|136501|estExt_fgenesh1_pg.C_460047 | 712 | - | 1 | + | + |
| Ps136506 | jgi|Physo1_1|136506|estExt_fgenesh1_pg.C_460052 | 701 | + | 4 | + | + |
| Ps138087B* | jgi|Physo1_1|138087|estExt_fgenesh1_pg.C_600046 | 1567 | + | 9 | + | + |
| Ps138088 | jgi|Physo1_1|138088|estExt_fgenesh1_pg.C_600047 | 773 | + | 6 | + | + |
| Ps139996 | jgi|Physo1_1|139996|estExt_fgenesh1_pg.C_800080 | 990 | + | 0 | + | + |
| Ps139997 | jgi|Physo1_1|139997|estExt_fgenesh1_pg.C_800081 | 781 | + | 5 | + | + |
| Ps140003 | jgi|Physo1_1|140003|estExt_fgenesh1_pg.C_800087 | 721 | - | 6 | + | + |
| Ps140146 | jgi|Physo1_1|140146|estExt_fgenesh1_pg.C_820047 | 747 | + | 6 | + | + |
| Ps140986 | jgi|Physo1_1|140986|estExt_fgenesh1_pg.C_920049 | 761 | + | 4 | + | + |
| Ps140988 | jgi|Physo1_1|140988|estExt_fgenesh1_pg.C_920051 | 1412 | - | 3 | + | + |
| Ps140991 | jgi|Physo1_1|140991|estExt_fgenesh1_pg.C_920054 | 741 | + | 4 | + | + |
| Ps141628 | jgi|Physo1_1|141628|estExt_fgenesh1_pg.C_1010041 | 865 | + | 4 | + | + |
| Ps144068 | jgi|Physo1_1|144068|estExt_fgenesh1_pg.C_1640023 | 749 | + | 5 | + | + |
| Ps157753 | jgi|Physo1_1|157753|C_scaffold_60000004 | 1167 | + | 9 | - | + |
| Ps158080 | jgi|Physo1_1|158080|C_scaffold_80000017 | 819 | + | 6 | + | + |
| Pu03658 | PYU1_T003658 | 775 | - | 6 | + | + |
| Pu07161 | PYU1_T007161 | 670 | - | 3 | + | + |
| Pu08017 | PYU1_T008017 | 853 | - | 5 | - | + |
| Pu14970 | PYU1_T014970 | 663 | + | 4 | - | + |
| S01258T0 | SPRG_01258T0 | 632 | + | 2 | + | + |
| S01416T0 | SPRG_01416T0 | 684 | + | 3 | + | + |
| S01930T0 | SPRG_01930T0 | 653 | - | 3 | + | + |
| S02178T0 | SPRG_02178T0 | 552 | - | 2 | + | + |
| S02311T0 | SPRG_02311T0 | 771 | + | 6 | + | + |
| S02316T0 | SPRG_02316T0 | 762 | - | 4 | + | + |
| S03267T0 | SPRG_03267T0 | 626 | - | 4 | + | + |
| S03896T0 | SPRG_03896T0 | 550 | - | 2 | + | + |
| S04928T0 | SPRG_04928T0 | 849 | - | 10 | + | + |
| S05673T0 | SPRG_05673T0 | 965 | - | 5 | + | + |
| S05981T0 | SPRG_05981T0 | 710 | - | 4 | + | + |
| S06057T0 | SPRG_06057T0 | 538 | - | 2 | + | + |
| S06128T0 | SPRG_06128T0 | 583 | - | 3 | + | + |
| S08428T0 | SPRG_08428T0 | 536 | + | 4 | + | + |
| S08432T0 | SPRG_08432T0 | 559 | - | 5 | - | + |
| S08727T0 | SPRG_08727T0 | 628 | + | 3 | + | + |
| S08987T0 | SPRG_08987T0 | 547 | - | 2 | + | + |
| S09249T0 | SPRG_09249T0 | 602 | + | 4 | + | + |
| S09257T0 | SPRG_09257T0 | 676 | - | 4 | + | + |
| S09298T0 | SPRG_09298T0 | 603 | - | 4 | - | + |
| S09308T0 | SPRG_09308T0 | 688 | - | 3 | + | + |
| S09505T0 | SPRG_09505T0 | 663 | - | 2 | + | + |
| S09620T0 | SPRG_09620T0 | 958 | + | 6 | + | + |
| S12826T0 | SPRG_12826T0 | 657 | - | 2 | + | + |
| S12836T0 | SPRG_12836T0 | 605 | - | 4 | + | + |
| S13082T0 | SPRG_13082T0 | 631 | + | 4 | + | + |
| S13910T0 | SPRG_13910T0 | 495 | - | 1 | + | + |
| S13911T0 | SPRG_13911T0 | 765 | + | 6 | + | + |
| S14923T0 | SPRG_14923T0 | 621 | - | 2 | + | + |
| S14924T0 | SPRG_14924T0 | 685 | - | 2 | + | + |
| S15453T0 | SPRG_15453T0 | 549 | - | 4 | + | + |
| S15543T0 | SPRG_15543T0 | 703 | + | 4 | + | + |
| S15848T0 | SPRG_15848T0 | 619 | - | 3 | + | + |
| S15849T0 | SPRG_15849T0 | 616 | - | 2 | + | + |
| S15852T0 | SPRG_15852T0 | 626 | - | 3 | + | + |
| S17043T0 | SPRG_17043T0 | 746 | + | 5 | + | + |

* when 2 KDs were predicted, they have been noted A and B

| Mb10450 | **jgi|Monbr1|10450|fgenesh1_pg.scaffold_21000102** | **1206** | **+** | **17** | **+** | **+** |
| --- | --- | --- | --- | --- | --- | --- |
| Mb25375 | jgi|Monbr1|25375|fgenesh2_pg.scaffold_10000025 | 1879 | - | 26 | - | + |
| Mb27170 | jgi|Monbr1|27170|fgenesh2_pg.scaffold_17000158 | 1235 | + | 0 | + | + |
| Mb28923 | jgi|Monbr1|28923|fgenesh2_pg.scaffold_30000008 | 1299 | - | 20 | + | + |
| Mb34096 | jgi|Monbr1|34096|estExt_fgenesh2_pg.C_300004 | 2002 | + | 15 | + | + |
| Mb34608 | jgi|Monbr1|34608|estExt_fgenesh2_pg.C_420022 | 825 | + | 3 | + | + |
| Mb36426 | jgi|Monbr1|36426|estExt_fgenesh1_pg.C_50276 | 1168 | + | 16 | + | + |
| Mb36839 | jgi|Monbr1|36839|estExt_fgenesh1_pg.C_80139 | 1310 | + | 16 | + | + |
| Mb37485 | jgi|Monbr1|37485|estExt_fgenesh1_pg.C_130220 | 1477 | - | 0 | + | **+** |
| Mb37167 | jgi|Monbr1|37167|estExt_fgenesh1_pg.C_110051 | 1541 | + | 0 | + | + |
| Mb28586 | jgi|Monbr1|28586|fgenesh2_pg.scaffold_27000062 | 1694 | + | 0 | + | + |
| Mb37923 | jgi|Monbr1|37923|estExt_fgenesh1_pg.C_180023 | 1311 | - | 16 | + | + |

| Esi0009_0077 |  | 900 | + | 6 | + | + |
| --- | --- | --- | --- | --- | --- | --- |
| Esi0009_0083 |  | 880 | + | 6 | + | + |
| Esi0020_0071 |  | 1089 | - | 0 | - | + |
| Esi0173_0029 |  | 1163 | + | 7 | - | + |

| Ch50123 | jgi|ChlNC64A_1|50123|fgenesh3_pg.C_scaffold_2000368 | **611** | **+** | **5** | **+** | **+** |
| --- | --- | --- | --- | --- | --- | --- |
| Ch136834 | jgi|ChlNC64A_1|136834|IGS.gm_17_00238 | 1252 | + | 13 | + | + |
| Ch137605 | jgi|ChlNC64A_1|137605|IGS.gm_2_00072 | 836 | + | 4 | + | + |
| Ch141452 | jgi|ChlNC64A_1|141452|IGS.gm_34_00056 | 1373 | + | 7 | + | + |
| Ch143410 | jgi|ChlNC64A_1|143410|IGS.gm_57_00001 | 908 | + | 6 | + | + |
| Ch136568 | jgi|ChlNC64A_1|136568|IGS.gm_16_00291 | 867 | + | 7 | - | + |
| Ch56654 | jgi|ChlNC64A_1|56654|estExt_fgenesh3_pg.C_20040 | 888 | + | 4 | - | + |
| Ch137597 | jgi|ChlNC64A_1|137597|IGS.gm_2_00064 | 879 | - | 6 | - | **+** |
| Ch141610 | jgi|ChlNC64A_1|141610|IGS.gm_36_00030 | 270 | - | 0 | - | + |
