# Supplementary material for: Leucine-Rich repeat receptor kinases are sporadically distributed in eukaryotic genomes
Source: BMC Evol Biol. 2011 Dec 20;11:367. doi: 10.1186/1471-2148-11-367 (PMC3268121; doi:10.1186/1471-2148-11-367)
Supplement: Additional file 4 — Alignment of LRR-RKs and reference eukaryotic KDs. The KDs of LRR-RKs from Ectocarpus siliculosus (Esi), Phytophthora ramorum (Pr), Phytophthora sojae (Ps), Phytophthora infestans (Pi), Saprolegnia parasitica (S), Pythium ultimum (Pu), Chlorella variabilis NC64A (Ch) and Monosiga brevicollis (Mb) were aligned with reference eukaryotic kinases from human (h), Arabidopsis thaliana (At), Drosophila melanogaster (dm), Danio rerio (dr), mouse (m) and Xenopus tropicalis (Xt). The positions of the 12 kinase subdomains are shown in roman numerals. [file 1471-2148-11-367-S4.DOC]

**Additional file 4: Alignment of LRR-RKs and reference eukaryotic KDs.**

**I** **II**

------------ ----

**10 20 30 40 50 60 70 80 90 100**

**....|....|....|....|....|....|....|....|....|....|....|....|....|....|....|....|....|....|....|....|**

**AT1G06840_LRRVIII-1** FNSST-------QI----------GQGGYG---KVYKGTLG----------------------------S--------------------GTV-VAIK--

**AT4G29180_LRRI**  NNFNK-------VI----------GKGGFG---IVYLGSLE----------------------------D--------------------GTE-IAVK--

**AT3G14840_LRRVIII-2** FDPAN-------KI----------GEGGFG---PVHKGIMT----------------------------D--------------------GTV-IAVK--

**AtNIK1_LRRII**  FSSKN-------LL----------GKGGYG---NVYKGILG----------------------------D--------------------STV-VAVK--

**AtNAK**  FRPDS-------VV----------GEGGFG---CVFKGWID----------------------------E----------SSLAPSKPGTGIV-IAVK--

**AT2G24230_LRRVII**  FDRDT-------LL----------ADGKFG---PVYRGFLP----------------------------G--------------------GIH-VAVK--

**AtBRI1_LRRXb**  FHNDS-------LI----------GSGGFG---DVYKAILK----------------------------D--------------------GSA-VAIK--

**AtCLV1_LRRXI**  LKEEN-------II----------GKGGAG---IVYRGSMP----------------------------N--------------------NVD-VAIK--

**AtFEI1_LRRXIIIa**  LNEEH-------II----------GCGGFG---TVYKLAMD----------------------------D--------------------GKV-FALK--

**AtER_LRRXIIIb**  LSEKY-------II----------GHGASS---TVYKCVLK----------------------------N--------------------CKP-VAIK--

**AtRPK1_LRRXV**  FSNSN-------CI----------GHGGFG---STYKAEVS----------------------------P--------------------TNV-FAVK--

**AtTMK1_LRRIX**  FSSDN-------IL----------GSGGFG---VVYKGELH----------------------------D--------------------GTK-IAVK--

**AT3G28450_LRRXa**  FNSEN-------II----------VSTRTG---TTYKALLP----------------------------D--------------------GSA-LAVK--

**AtIMK3_LRRIII**  CATAE-------IM----------GKSTYG---TVYKATLE----------------------------D--------------------GSQ-VAVK--

**AT2G45340_LRRIV**  FSEIN-------LL----------GKSNVS---SVYKGILR----------------------------D--------------------GSV-AAIK--

**AtSCM_SUB_LRRV**  FSEEN-------II----------GEGSIG---NVYRAELR----------------------------H--------------------GKF-LAVK--

**AtFLS2_LRRXII**  FNSAN-------II----------GSSSLS---TVYKGQLE----------------------------D--------------------GTV-IAVK--

**dmPELLE**  WSPDN-------RL----------GQGGFG---DVYRGKWK----------------------------QLD----------------------VAIK--

**drIRAK1**  FSPCK-------QI----------GEGGFG---HVYKAVMR----------------------------N---------------------TE-FAVK--

**hIRAK1**  FSEEL-------KI----------GEGGFG---CVYRAVMR----------------------------N---------------------TV-YAVK--

**xtPELLE**  FSQSL-------LI----------GEGGFG---CVYKATMR----------------------------N---------------------TE-YAVK--

**AtCTR1_Raf**  LNIKE-------KI----------GAGSFG---TVHRAEWH----------------------------G-S--------------------D-VAVK--

**Mb27170**  VRRID-------KE----------APGAFG---EVWLCRFN----------------------------DID----------------------VAVK--

**Mb28586**  ETRID-------AA----------AMGTYG---QVWRAKYH----------------------------DRS----------------------VAVK--

**hRaf1**  VMLST-------RI----------GSGSFG---TVYKGKWH----------------------------G----------------------D-VAVK--

**Mb37485**  LRNFR-------LL----------DKGTFA---EVYRAQWG----------------------------GEL----------------------VAVK--

**Esi0009_0077**  EVTNE-------VL----------GRGGFG---TVYLADLH----------------------------G-L--------------------N-AAAK--

**Esi0009_0083**  EVTNE-------VL----------GRGGFG---TVYLADLH----------------------------G-L--------------------N-AAAK--

**Esi0020_0071**  TITNE-------VL----------GKGGFG---EVYLADYN----------------------------G-R--------------------N-AAAK--

**Esi0173_0029**  VITEE-------ML----------GRGGFG---EVYLADYN----------------------------G-H--------------------N-AAAK--

**Ch136568**  IEILRRPDGADWQL----------GSGGFG---TVYKAMRN----------------------------GVQ--------------------P-VAVK--

**Ch50123**  ------------RL----------GSGGFA---TVYKAMHN----------------------------GVQ--------------------P-VAVK--

**Ch137597**  -QLV--------EL----------GEGAHA---VVYLARLQ----------------------------GVE----------------------VAVK--

**Ch137605**  -QLV--------EL----------GEGGHA---VVYLARLQ----------------------------GVE----------------------VAVK--

**Ch56654**  GRPV--------EL----------GQGGFG---VVYMARLQ----------------------------GVE----------------------VAVK--

**Ch141610**  -RLV--------EL----------GEGAYA---VVYLGSLS----------------------------GAQ----------------------VAVK--

**Ch143410**  -QLV--------EL----------GEGAHA---VVYLGKLS----------------------------GSH----------------------VAVK--

**Ch141452**  -ELV--------LL----------GEGGFA---SVYQARLG----------------------------DQP----------------------VAVKAR

**Mb10450**  ----E-------RIDGS-------IPGTFG---EVYRAEWG----------------------------KFE----------------------VCVK--

**Mb25375**  LELLK-------RIDGD-------SPGAFG---EVWGARWQ----------------------------DLE----------------------VCVK--

**Mb28923**  IALEE-------RIDGD-------SPGAFG---EVWRAQWQ----------------------------GFS----------------------VCVK--

**Mb37923**  LQLLN-------RIDGD-------SPGAFG---EVWRAEWN----------------------------RFE----------------------VCVK--

**Mb34096**  IRLLE-------RIDGD-------SQGAFG---SVYAADWD----------------------------HMR----------------------VCVK--

**Mb36839**  LDLE--------------------SPGASG---EVHSGTWG----------------------------NVR----------------------VAVK--

**Mb36426**  IILGR-------RIDVG-------YEGAFG---DVYQGQWN----------------------------SFV----------------------VAVK--

**hAXL**  VALGK-------TL----------GEGEFG---AVMEGQLN----------------------------Q-----------------DDSILK-VAVK--

**hHGFR**  VHFNE-------VI----------GRGHFG---CVYHGTLL----------------------------DND----------------GKKIH-CAVK--

**hRYK**  ITLKD-------VL----------QEGTFG---RIFHGILI----------------------------D-E-----------KDP--NKEKQ-AFVK--

**hDDR**  LRFKE-------KL----------GEGQFG---EVHLCEVD----------------------------S-PQDLVSLDFPLNVRK--GHPLL-VAVK--

**hIR**  ITLLR-------EL----------GQGSFG---MVYEGNAR----------------------------D-I-----------IKG--EAETR-VAVK--

**hLTK**  VTLLR-------AL----------GHGAFG---EVYEGLVI----------------------------G-L-----------PGD--SSPLQ-VAIK--

**hMuSK**  IEYVR-------DI----------GEGAFG---RVFQARAP----------------------------G-L-----------LPY--EPFTM-VAVK--

**hTRKalpha**  IVLKW-------EL----------GEGAFG---KVFLAECH----------------------------N-L-----------LPE--QDKML-VAVK--

**hKLGlikePTK7**  LQPIT-------TL----------GKSEFG---EVFLAKAQ----------------------------G-L-----------EEG--VAETL-VLVK--

**hFGFR2**  LTLGK-------PL----------GEGCFG---QVVMAEAV----------------------------G-I-----------DKDKPKEAVT-VAVK--

**hRET**  LVLGK-------TL----------GEGEFG---KVVKATAF----------------------------H-L-----------KGR--AGYTT-VAVK--

**hVGFR1**  LKLGK-------SL----------GRGAFG---KVVQASAF----------------------------G-I-----------KKS--PTCRT-VAVK--

**hPDGFRbeta**  LVLGR-------TL----------GSGAFG---QVVEATAH----------------------------G-L-----------SHS--QATMK-VAVK--

**hTIE1**  ITFED-------LI----------GEGNFG---QVIRAM--------------------------------I-----------KKD--GLKMN-AAIK--

**hEGFR**  FKKIK-------VL----------GSGAFG---TVYKGLWI-------------------------------------------PEGEKVKIP-VAIK--

**hEPH**  LMVDT-------VI----------GEGEFG---EVYRGTLR--------------------------------------------LPSQDCKT-VAIK--

**Pi00640T0**  LHVGR-------CI----------SKGGFG---LVYVGMYK----------------------------H-R--------------------R-VAIK--

**Pi19256T0**  --------------------------------------MYK----------------------------H-R--------------------R-VAIK--

**Pr72884**  LHVGR-------CI----------SKGGFG---LVYTGMYK----------------------------H-R--------------------Q-VAIK--

**Ps140986**  LHIGR-------CI----------SKGGFG---LVYTGMYK----------------------------H-R--------------------R-VAIK--

**Pi00643T0**  VALGR-------CI----------SRGGFG---LVFVGCYR----------------------------G-R--------------------Q-VAVK--

**Pr72888**  VALGR-------CI----------SRGGFG---LVFVGSYR----------------------------G-Q--------------------Q-VAVK--

**Ps140988**  VTLGR-------CI----------SRGGFG---LVFVGSYR----------------------------G-R--------------------Q-VAVK--

**Pi00646T0**  VKVAN-------CI----------NKGGFG---LVYSGVYN----------------------------R-R--------------------R-VAIK--

**Ps140991**  VKVAN-------CI----------NKGGFG---LVYSGVYN----------------------------R-R--------------------R-VAIK--

**Pr72890**  VKVAN-------CI----------NKGGFG---LVYSGVYN----------------------------R-R--------------------R-VAIK--

**Pi17831T0**  LRLGK-------CI----------SKGGFG---LVFTGEYR----------------------------G-R--------------------R-VAIK--

**Ps132644**  LRLGK-------CI----------SKGGFG---LVFTGEYK----------------------------G-R--------------------R-VAIK--

**Pi17840T0**  LKLGQ-------CI----------SKGGFG---LVFLGEYK----------------------------G-R--------------------R-VAIK--

**Pr76005**  LKMGR-------CI----------STGGFG---LVFMGEYR----------------------------G-R--------------------R-VAIK--

**Ps132640**  LKMGK-------CI----------SKGGFG---LVFMGEYK----------------------------G-R--------------------R-VAIK--

**Pi17832T0**  IKLDK-------CL----------SKGGFG---LVFVGEYR----------------------------G-R--------------------Q-VAVK--

**Pr76010**  VKIGK-------CL----------SRGGFG---LVFVGEYK----------------------------G-R--------------------Q-VAVK--

**Ps132643**  VKLGK-------CL----------SRGGFG---LVFVGQYK----------------------------G-R--------------------Q-VAVK--

**Pi05112T0**  VEAVK-------LL----------SRGGFG---EVWLGLYM----------------------------N-E--------------------N-VAIK--

**Pr82847**  VEAIK-------LL----------SRGGFG---EVWLGLFM----------------------------N-E--------------------N-VAIK--

**Ps132270**  VEAVK-------LL----------SRGGFG---EVWLGLYM----------------------------N-E--------------------N-VAIK--

**Pu14970**  VEAIK-------LL----------SRGGFG---EVWLGLYM----------------------------N-E--------------------N-VAIK--

**S05981T0**  VQEIQ-------LI----------SRGGFG---EVWSGIYL----------------------------G-R--------------------P-VAIK--

**Pr81631**  VTLVK-------KI----------AQGAFG---EVWVGQYR----------------------------N-S--------------------R-VAVK--

**Pi02124T0**  LNYTR-------KI----------AKGAFG---EVWLALYG----------------------------K-D--------------------L-VAVK--

**Pr73421**  LNYTR-------KI----------AKGAFG---EVWLALHG----------------------------K-D--------------------L-VAVK--

**Ps144068**  INYTR-------KI----------AKGAFG---EVWLALHG----------------------------K-D--------------------L-VAVK--

**S04928T0**  MVYTR-------TL----------GKGAKG---VVWLASYA----------------------------G-D--------------------V-VAVK--

**Pi07724T0**  VKDLK-------QL----------GGGAFA---IVWLVNYR----------------------------D-S--------------------QLLASK--

**Ps158080**  VKDLK-------QL----------GNGAFA---MVWLVRYR----------------------------D-S--------------------QLLASK--

**Pr77475A**  VKDMK-------QL----------GSGAFA---MVWLVRYR----------------------------D-S--------------------QLLASK--

**Pi07725T0**  IEDVK-------QL----------GIGAFA---TVWLVQYR----------------------------K-S--------------------QLLASK--

**Pi21299T0**  IEDVK-------QL----------GIGAFA---TVWLVQYR----------------------------K-S--------------------QLLASK--

**Pr77476**  VEDIK-------QL----------GSGAFA---TVWLVRYR----------------------------K-S--------------------QMLASK--

**Ps140003**  IEDLE-------QL----------GSGAFA---TVWLVRYR----------------------------N-S--------------------QLLASK--

**Pi07731T0**  IKDLK-------PL----------GSGAYA---TVWLVRYR----------------------------N-L--------------------QLLASK--

**Ps139996**  IKDLK-------PL----------GTGAYA---TVWLVRYR----------------------------N-L--------------------QLLASK--

**Ps139997**  IKDLK-------PL----------GTGAYA---TVWLVRYR----------------------------N-L--------------------QLLASK--

**Pr77470**  IKDIK-------QL----------GSGAYA---TVWLVHYR----------------------------K-L--------------------QILASK--

**Pr77475B**  IEDIR-------QL----------GSGAYA---TVWLVRYR----------------------------E-S--------------------QLLASK--

**Pi09664T0**  IEDIG-------TI----------GSGAFG---IVWLVKYR----------------------------G-S--------------------QLLASK--

**Pr75814**  IEDIG-------TI----------GSGAFG---IIWLVKYR----------------------------G-S--------------------QLLASK--

**Ps135332**  IEDIG-------TI----------GSGAFG---IIWLVKYR----------------------------G-N--------------------QLLASK--

**Ps135334**  IEDIG-------TI----------GSGAFG---IIWLVKYR----------------------------G-N--------------------QLLASK--

**Pi23090T0**  IQDVK-------LI----------GSGAFG---VVWLVRYR----------------------------S-A--------------------QLLASK--

**Pi23143T0**  IQDVK-------LI----------GSGAFG---VVWLVRYR----------------------------S-A--------------------QLLASK--

**Pr81229**  IQDVR-------LI----------GSGAFG---VVWLVRYR----------------------------N-A--------------------QLIASK--

**Pr75791**  IQDIR-------LI----------GSGSFG---VVWLVRYR----------------------------N-S--------------------ELLASK--

**Pi09665T0**  IEDIR-------KL----------GTGAFG---VVYLAKYR----------------------------Q-N--------------------RLVACK--

**Ps135333**  IEDVR-------KL----------GTGAFG---VVYLAKYR----------------------------K-N--------------------KLVACK--

**Ps127796**  IKNVK-------KI----------GAGGHC---VVWLVRYR----------------------------K-S--------------------QLLASK--

**Pi13397T0**  VHIER-------SI----------AKGGFG---IVYLANYQ----------------------------S-R--------------------S-VVVK--

**Ps140146**  VQIER-------SI----------AKGGFG---IVFLATYQ----------------------------S-R--------------------P-VVVK--

**Pr81472**  VQIER-------SI----------AKGGFG---IVFLAKYQ----------------------------G-R--------------------T-VVVK--

**Pi14970T0**  IVIQE-------EL----------ASGGFG---VVHLAQLY----------------------------G-Q--------------------R-VVVK--

**Ps138087B**  VVIQK-------EL----------ASGGFG---VVHLAQLY----------------------------G-Q--------------------R-VVVK--

**Pr75692**  IIIER-------EL----------ASGGFG---VVYLAGLY----------------------------S-Q--------------------L-VVVK--

**Pi14971T0**  VIRGK-------LI----------AKGGYG---AVYKATFR----------------------------G-K--------------------T-VVSK--

**Pi14972T0**  VVRGK-------LL----------AKGGYG---AVYLATFQ----------------------------S-E--------------------K-VVTK--

**Pr75691A**  VVRGK-------LL----------AKGGYG---AVYLATFQ----------------------------N-E--------------------K-VVTK--

**Ps138088**  VVRGK-------LL----------AKGGYG---AVYMATFQ----------------------------N-E--------------------K-VVTK--

**S06057T0**  IVMQQ-------TI----------ASGGFG---VVYLARYH----------------------------S-M--------------------D-VAVK--

**S02178T0**  IVRDR-------VI----------ARGGFG---IVYLATIYPSTRMP----------------------P-L--------------------R-VAMK--

**S02311T0**  IVRDR-------VL----------ARGGFG---IVYLATLQTEKGR-----------------------A-Q--------------------L-VAVK--

**S02316T0**  IERQS-------IL----------ARGGFG---IVYLATLQPTPTLPDVLS------------------P-T--------------------R-VALK--

**S13911T0**  ILYKE-------VV----------AKGGYG---VVYVATMVLRGR------------------------E-T--------------------K-VAMK--

**Pi05340T0**  LEVVQ-------QL----------SKGAGG---VVHLAMWKPK--------------------------N-Q--------------------Q-VVVK--

**Ps133283**  LEFVK-------QL----------SKGAGG---VVNLAMWRSR--------------------------K-E--------------------K-IVMK--

**Ps133286**  LEFVK-------QL----------SKGAGG---VVNLAMWRSR--------------------------K-E--------------------K-IVMK--

**Pr80778**  RRTKA-------AL----------AEKVK-----AEQAANESSSDDDDSDKMKMTSAILTDDVRGDPDFE-R--------------------Y-LAVK--

**S15848T0**  LLHKR-------VC----------GSGGFA---VVYKATFQ----------------------------A-Q--------------------I-VAVK--

**S15849T0**  LLHKQ-------IC----------GNGGFA---IVYKALFQ----------------------------D-Q--------------------V-VAVK--

**S15852T0**  LLQKQ-------IC----------GNGGFA---IVYKALFQ----------------------------D-Q--------------------V-VAVK--

**S01258T0**  VLGDK-------PL----------AAGAFG---EVWKGTYA----------------------------N-E--------------------V-VAIK--

**S09257T0**  VTSNK-------PL----------ASGAFG---EVWLGTYG----------------------------G-K--------------------Q-VAIK--

**S15543T0**  VTASK-------PL----------ASGAFG---EVWLGSYG----------------------------G-K--------------------Q-VAIK--

**S09298T0**  VTAKK-------PL----------ASGAFG---EVWLATYC----------------------------D-E--------------------K-VAVK--

**S15453T0**  VTAKK-------PL----------ASGAFG---EVWLATYC----------------------------D-E--------------------K-VAVK--

**S08727T0**  TVGSK-------PI----------ASGAYG---DVWRGRYL----------------------------K-E--------------------D-VAIK--

**S09308T0**  LNPIK-------PL----------ASNAYG---EVWLSHFG----------------------------A-D--------------------V-VVVQ--

**S01416T0**  VVPTR-------LL----------ASGAFA---DVYLGEYK----------------------------G-E--------------------P-VAIK--

**S06128T0**  VVIKD-------KI----------ATGAFG---EVWRAEYK----------------------------H-K--------------------T-VAVK--

**S12836T0**  VMLLR-------KL----------SEGAFG---EVWKGTLH----------------------------S-E--------------------I-VAVK--

**S17043T0**  VELHK-------KL----------AEGAYG---EVWKGSYN----------------------------G-A--------------------I-VAVK--

**S08987T0**  LVKLQ-------FV----------AEGAYG---QVSRGAYK----------------------------G-E--------------------P-VAIK--

**S13082T0**  LAAIK-------FV----------AEGAYG---QVWLGTYE----------------------------G-N--------------------P-VALK--

**S05673T0**  KLLDT-------IL----------GRGAYG---EVVLGDYN----------------------------G-R--------------------L-VAVK--

**S09620T0**  KLLDT-------IL----------GRGAYG---EVVLGDYN----------------------------G-R--------------------L-VAVK--

**S12826T0**  ILLLA-------PM----------SQGSYG---EVWLAQYQ----------------------------N-E--------------------T-IAIK--

**S03267T0**  ISMEK-------RL----------AQGGYG---EVWLGHYH----------------------------A-S--------------------V-VAIK--

**S13910T0**  LTRGH-------LL----------YRRELS---LVHRALYQAAGADD----------------------A-E--------------------M-VTLT--

**Pi05338T0**  VMQVR-------TL----------AHGSFA---MTSLVHLG----------------------------D-K--------------------Q-AVMK--

**Pr80780**  VMQVR-------TL----------AHGNFA---MTSLVYLG----------------------------D-K--------------------Q-AVMK--

**Ps133281**  VMQVR-------TL----------AHGSFA---MASLVYLG----------------------------D-K--------------------Q-AVMK--

**Pi22892T0**  ---------------------------------EAFLTRFR----------------------------G-S--------------------RFLVCK--

**Pr94275**  ---------------------------------VTFLTKFR----------------------------G-S--------------------RLLICK--

**Ps135323**  LEYIR-------KLT---------SEHRARSNRVTFLTRFR----------------------------G-S--------------------RFLVCK--

**S01930T0**  ---------------------------------EVWLGTFE----------------------------D-S--------------------H-VAIK--

**Ps133943**  RQTLR-------KQ----------SVSSAMNPREIWLASYS----------------------------SGK-----HSAAAVSPGGGTTGTL-VVAK--

**S03896T0**  LNDVQ-------ILGPLHGKHKNASVPGTM---MLYKAEFD----------------------------GRL----------------------VVLK--

**S09249T0**  VV----------VL----------DRLSTG---DLWKGLFE----------------------------GHV----------------------VSIR--

**Pi03775T0**  L-----------------------RVSSRS---ELWLGEYK----------------------------HDA----------------------VLVK--

**Pr75312**  L-----------------------RVSSRS---ELWLGEYK----------------------------H-E--------------------V-VLVK--

**Ps136506**  L-----------------------RVSSRS---ELWLGEYK----------------------------H-E--------------------A-VLVK--

**AT3G14840_LRRXIV**  FSDSN-------LI----------KKGQSG---DLFKGVLE----------------------------N-G-------------------VQ-IVVK--

**Ps141628**  SFS------------------------AF----VYFKAAYR----------------------------G-K--------------------T-VVLK--

**Pu08017**  ATSEG-------EI----VRGSGANTPGF----AYYTAIFK----------------------------S-Q--------------------Q-VVLK--

**S14923T0**  IKVIL-------------------PISAKC---SMWIGRFK----------------------------G-R--------------------E-VVMK--

**S14924T0**  CRILK-------AM----------GP-------AVFAGQYK----------------------------GEN----------------------VVVR--

**Mb37167**  LKWER-------NL----------DEGGFG---NVYLATWL----------------------------S-Q------------------DKR-VAVK--

**AtAME2**  YQILS-------KM----------GEGTFG---QVLECFDN----------------------------K-N------------------KEV-VAIK--

**hCLK1**  YEIVD-------TL----------GEGAFG---KVVECIDH----------------------------KAG------------------GRH-VAVK--

**AtCDC2a**  YEKVE-------KI----------GEGTYG---VVYKARDK----------------------------V-T------------------NET-IALK--

**hCDK3**  FQKVE-------KI----------GEGTYG---VVYKAKNR----------------------------E-T------------------GQL-VALK--

**AtMPK1**  YMPIK-------PI----------GRGAYG---VVCSSVNS----------------------------D-T------------------NEK-VAIK--

**hMAPK1**  YTNLS-------YI----------GEGAYG---MVCSAYDN----------------------------V-N------------------KVR-VAIK--

**AtGSK3b**  YMAER-------VV----------GTGSFG---IVFQAKCL----------------------------E-T------------------GES-VAIK--

**AtGSK3i**  YMAER-------VV----------GTGSFG---IVFQAKCL----------------------------E-T------------------GES-VAIK--

**AtCKA1**  YEVVR-------KV----------GRGKYS---EVFEGINV----------------------------N-S------------------KEK-CIIK--

**hCK2a**  YQLVR-------KL----------GRGKYS---EVFEAINI----------------------------T-N------------------NEK-VVVK--

**AtMEKK1**  WQKGQ-------LL----------GRGSFG---SVYEGISG----------------------------D--------------------GDF-FAVK--

**hMAPKKK1**  WLKGQ-------QI----------GLGAFS---SCYQAQDV----------------------------G-T------------------GTL-MAVK--

**AtMKK3**  MRVFG-------AI----------GSGASS---VVQRAIHI----------------------------P-N------------------HRI-LALK--

**hMAPKK1**  FEKIS-------EL----------GAGNGG---VVFKVSHK----------------------------P-S------------------GLV-MARK--

**AtCPK7**  YDLGR-------EV----------GRGEFG---ITYLCTDK----------------------------E-T------------------GEK-YACK--

**hCaMK1**  YDFRD-------VL----------GTGAFS---EVILAEDK----------------------------R-T------------------QKL-VAIK--

**AtNPH1**  FKPVK-------PL----------GSGDTG---SVHLVELV----------------------------G-T------------------DQL-FAMK--

**AtPVPKlikePK5**  FRLLK-------RL----------GCGDIG---SVYLSELS----------------------------G-T------------------KCY-FAMK--

**AtS6KlikePK1**  FEVMK-------VV----------GKGAFG---KVYQVRKK----------------------------E-T------------------SEI-YAMK--

**hGRK6**  FRQYR-------VL----------GKGGFG---EVCACQVR----------------------------A-T------------------GKM-YACK--

**AtSnRK2**  YDFVK-------DI----------GSGNFG---VARLMTDR----------------------------V-T------------------KEL-VAVK--

**OUTGROUP1**  FRVLQ-------PL----------GSGGMG---QVYLGEQV----------------------------S-L------------------GRK-VAIK--

**OUTGROUP2**  YDIKM-------LI----------GDGGMA---NVYLAYDR----------------------------T-L------------------KRH-VAIK--

**OUTGROUP3**  YKILD-------HL----------GTGGMA---TVWLGYDT----------------------------I-L------------------DRQ-VAIK--

**OUTGROUP4**  YELGE-------IL----------GFGGMS---EVHLARDL----------------------------R-L------------------HRD-VAVK--

**AtTousled**  YALLN-------LL----------GKGGFS---EVYKAYDL----------------------------V-D------------------HRY-VACK--

**hTousledLK1**  YLLLH-------LL----------GRGGFS---EVYKAFDL----------------------------Y-E------------------QRY-AAVK--

**S08428T0**  LRLQA-------TI-----------DGQ-----VLYLGAHV----------------------------QTK----------------------VCIK--

**S08432T0**  IKKLK-------LL----------SSTAYT---TMYVAERY----------------------------GAK----------------------VHAT--

**Pi03773T0**  ---LN-------QL----------SASGPV---VVALAEHR----------------------------TKL----------------------VLVT--

**Pr75310**  -------------L----------SMSGPI---TVALAEHC----------------------------TRL----------------------VLVT--

**Ps136501**  VVVLN-------QL----------STSGPV---TVALAEHC----------------------------TRL----------------------VLVT--

**Pu07161**  LQMLN-------VV----------TTNGVV---SVKKAKYN----------------------------KKL----------------------VYVT--

**S09505T0**  ------------LL-----------------------AKVR----------------------------GSP----------------------VYLN--

**AT1G14390_LRRVI-1**  ------------NLC---------GE-------QLYKGCLR----------------------------E--------------------GIA-VTVR--

**AT5G41180_LRRVI-2**  EDFSN-------II----------DSSAES---QIYKGTIK----------------------------G--------------------GTE-IAVI--

**hTGFbRI**  IVLQE-------SI----------GKGRFG---EVWRGKWR----------------------------G---------------------EE-VAVK--

**hTGFbRII**  IELDT-------LV----------GKGRFA---EVYKAKLK----------------------------Q---------------NTSEQFET-VAVK--

**Pi14992T0**  FHMHR-------IL----------SRSYHG---EVFLGDYC----------------------------GTQ----------------------VVVK--

**Ps157753**  FHMHR-------IL----------SRSYHG---EIFLGDYC----------------------------ATA----------------------------

**Pi04971T0**  ------------ALALSSVGVGNSRKKVDM---ALYLAEYQ----------------------------SYK----------------------VVIQ--

**Ps136072**  ------------ALALSTVGVGNPRKKMDS---ALFLAEYQ----------------------------GYK----------------------VVIQ--

**Pr93648**  ----SSKKNSKAALGFSSVGLGSAQKKVDH---ALFLAEYQ----------------------------GYK----------------------VAIQ--

**Pu03658**  YQHQA-----------------------------------------------------------------------------------------VAQR--

**AtCKI1**  FKLGR-------KL----------GSGSFG---ELYLGINI----------------------------Q-T------------------GEE-VAVK--

**hCKIalpha2**  YKLVR-------KI----------GSGSFG---DIYLAINI----------------------------T-N------------------GEE-VAVK--

**mCKIalpha**  YKLVR-------KI----------GSGSFG---DIYLAINI----------------------------T-N------------------GEE-VAVK--

**III** **IV**

**----------- --------**

**110 120 130 140 150 160 170 180 190 200**

**....|....|....|....|....|....|....|....|....|....|....|....|....|....|....|....|....|....|....|....|**

**AT1G06840_LRRVIII-1** ----------RAQEGSL---------------QGEK-EFLTEIELL--SRL-HH------RN---LVSLLGFC---------------------------

**AT4G29180_LRRI**  ----------MINDSSFGKSKGSSSSSSSS--QVSK-EFQVEAELL--LTV-HH------RN---LASFVGYC---------------------------

**AT3G14840_LRRVIII-2** ----------QLSAKSK---------------QGNR-EFLNEIAMI--SAL-QH------PH---LVKLYGCC---------------------------

**AtNIK1_LRRII**  ----------RLKDGGAL--------------GGEI-QFQTEVEMI--SLA-VH------RN---LLRLYGFC---------------------------

**AtNAK**  ----------RLNQEGF---------------QGHR-EWLAEINYL--GQL-DH------PN---LVKLIGYC---------------------------

**AT2G24230_LRRVII**  ----------VLVHGST---------------LSDQ-EAARELEFL--GRI-KH------PN---LVPLTGYC---------------------------

**AtBRI1_LRRXb**  ----------KLIHVSG---------------QGDR-EFMAEMETI--GKI-KH------RN---LVPLLGYC---------------------------

**AtCLV1_LRRXI**  ----------RLVGRGTG--------------RSDH-GFTAEIQTL--GRI-RH------RH---IVRLLGYV---------------------------

**AtFEI1_LRRXIIIa**  ----------RILKLNE---------------GFDR-FFERELEIL--GSI-KH------RY---LVNLRGYC---------------------------

**AtER_LRRXIIIb**  ----------RLYSHNP---------------QSMK-QFETELEML--SSI-KH------RN---LVSLQAYS---------------------------

**AtRPK1_LRRXV**  ----------RLSVGRF---------------QGDQ-QFHAEISAL--EMV-RH------PN---LVMLIGYH---------------------------

**AtTMK1_LRRIX**  ----------RMENGVIAG-------------KGFA-EFKSEIAVL--TKV-RH------RH---LVTLLGYC---------------------------

**AT3G28450_LRRXa**  ----------HLSTCK----------------LGER-EFRYEMNQL--WEL-RH------SN---LAPLLGFC---------------------------

**AtIMK3_LRRIII**  ----------RLRERS--------------------------------------------PK--------------------------------------

**AT2G45340_LRRIV**  ----------CIAKSSCK--------------SDES-EFLKGLKML--TLL-KH------EN---LARLRGFC---------------------------

**AtSCM_SUB_LRRV**  ----------KLSNTINRT-------------QSDG-EFLNLVSNV--LKL-KR------GH---ILELLGYC---------------------------

**AtFLS2_LRRXII**  ----------VLNLKEFSA-------------ESDK-WFYTEAKTL--SQL-KH------RN---LVKILGFA---------------------------

**dmPELLE**  ----------VMNYRSPNIDQKM---------VELQ-QSYNELKYL--NSI-RH------DN---ILALYGYS---------------------------

**drIRAK1**  ----------KLKEDS-----------HLDWNVVKE-SFRTEVDKL--SQY-RH------PN---IMDFVGYC---------------------------

**hIRAK1**  ----------RLKENA-----------DLEWTAVKQ-SFLTEVEQL--SRF-RH------PN---IVDFAGYC---------------------------

**xtPELLE**  ----------RLKQDS-----------ELEWSTMKK-SFLTEIEKL--TCL-RH------PN---IIDLAGYS---------------------------

**AtCTR1_Raf**  ----------ILMEQDFHA-------------ERVN-EFLREVAIM--KRL-RH------PN---IVLFMGAV---------------------------

**Mb27170**  ----------RLKGHLADMDP-----------SLNE-EFLNEIKFM--RTL-RH------PN---IVYFFGAG---------------------------

**Mb28586**  ----------LLNPVMVHMDE-----------NLTN-DFFREIRMM--RMI-RH------TN---CVFFYGAG---------------------------

**hRaf1**  ----------ILKVVDPTP-------------EQFQ-AFRNEVAVL--RKT-RH------VN---ILLFMGYM---------------------------

**Mb37485**  ----------KMHTALVEMRIWNR--------SITS-DFDREVRLL--QRT-RH------RN---IVLFYGAG---------------------------

**Esi0009_0077**  ----------VVSREHQ---------------RQRR-MFMRELEAM--KRL-RG------PH---TVTIYGAV---------------------------

**Esi0009_0083**  ----------VVSREHQ---------------RQRR-MFMRELEAM--KRL-RG------PH---TVTIYGAV---------------------------

**Esi0020_0071**  ----------HHQND-----------------SQRR-AFLRELDAM--IRL-RS------PH---TVNVYGAV---------------------------

**Esi0173_0029**  ----------VGKK------------------IQRK-AFLRELDAM--IRL-RH------PH---TVNVYGAI---------------------------

**Ch136568**  ----------ALGSVNSEVLKS----------MTDE-DFVQEISIL--RAC-RD------TN---ILQFQGAC---------------------------

**Ch50123**  ----------VLSTTVSHMKRA----------MSQA-AFAQEIAIL--RAC-RN------AN---ILQFQGAC---------------------------

**Ch137597**  ----------VFELQPG---------------LDPH-TAWREVSLL--RQC-AH------AR---IVPLFGVA---------------------------

**Ch137605**  ----------VTELLSG---------------VESQ-SIWRETALL--RRC-IH------ER---IVPLYGVA---------------------------

**Ch56654**  ----------VFELAPG---------------AASA-AMWREAALL--RRC-AH------ER---IVPLYGVA---------------------------

**Ch141610**  ----------VYELDHG---------------MDAS-VMWREAAML--RDA-VH------DH---IVPLYGVA---------------------------

**Ch143410**  ----------VFELGSG---------------VHSS-AVWREAAML--RDC-GH------AR---IVPLFGVA---------------------------

**Ch141452**  DAAAPAASGRVFELVAD---------------LDST-AIWREVALL--RQC-QH------PR---VVPVYGVA---------------------------

**Mb10450**  ----------VLRLNWLHMDP-----------MVRQ-DFEKEAEFL--QQT-KH------RN---LVRFFGLG---------------------------

**Mb25375**  ----------ILNASAAANF------------AAVQ-DFEKELDFL--QRT-RH------PH---LVRFFGAG---------------------------

**Mb28923**  ----------VLKASATLDT------------AAVE-DFQKELELL--QRT-RH------PQ---LVRFFGAG---------------------------

**Mb37923**  ----------VLQHQLSMET------------NVVN-DFEKEIDFL--QRT-RH------PH---LVRLFGAG---------------------------

**Mb34096**  ----------LLREGMSFDE------------MTEG-VFDREINFL--QRF-RH------PQ---LVRFFGAG---------------------------

**Mb36839**  ----------VLRASLAQFDE-----------RSRE-EFESEVAFL--QKT-RH------PN---LVRFFGAG---------------------------

**Mb36426**  ----------VAKARVGPMQT-----------DERA-MFQREVDFV--MRA-SH------PC---LVHFFGFA---------------------------

**hAXL**  ----------TMKIAICTR-------------SELE-DFLSEAVCM--KEF-DH------PN---VMRLIGVC---------------------------

**hHGFR**  ----------SLN-RITDI-------------GEVS-QFLTEGIIM--KDF-SH------PN---VLSLLGIC---------------------------

**hRYK**  ----------TVK-DQASE-------------IQVT-MMLTESCKL--RGL-HH------RN---LLPITHVC---------------------------

**hDDR**  ----------ILR-PDATK-------------NARN-DFLKEVKIM--SRL-KD------PN---IIRLLGVC---------------------------

**hIR**  ----------TVN-ESASL-------------RERI-EFLNEASVM--KGF-TC------HH---VVRLLGVV---------------------------

**hLTK**  ----------TLP-ELCSP-------------QDEL-DFLMEALII--SKF-RH------QN---IVRCVGLS---------------------------

**hMuSK**  ----------MLK-EEASA-------------DMQA-DFQREAALM--AEF-DN------PN---IVKLLGVC---------------------------

**hTRKalpha**  ----------ALK--EASE-------------SARQ-DFQREAELL--TML-QH------QH---IVRFFGVC---------------------------

**hKLGlikePTK7**  ----------SLQ--SKDE-------------QQQL-DFRRELEMF--GKL-NH------AN---VVRLLGLC---------------------------

**hFGFR2**  ----------MLK-DDATE-------------KDLS-DLVSEMEMM--KMIGKH------KN---IINLLGAC---------------------------

**hRET**  ----------MLK-ENASP-------------SELR-DLLSEFNVL--KQV-NH------PH---VIKLYGAC---------------------------

**hVGFR1**  ----------MLK-EGATA-------------SEYK-ALMTELKIL--THIGHH------LN---VVNLLGAC---------------------------

**hPDGFRbeta**  ----------MLK-STARS-------------SEKQ-ALMSELKIM--SHLGPH------LN---VVNLLGAC---------------------------

**hTIE1**  ----------MLK-EYASE-------------NDHR-DFAGELEVL--CKLGHH------PN---IINLLGAC---------------------------

**hEGFR**  ----------ELR-EATSP-------------KANK-EILDEAYVM--ASV-DN------PH---VCRLLGIC---------------------------

**hEPH**  ----------TLK-DTSPG-------------GQWW-NFLREATIM--GQF-SH------PH---ILHLEGVV---------------------------

**Pi00640T0**  ----------KIIRE--KCE-KLS---------QIR-MFIREITLM--GAL-KH------ER---IVEFIGVA---------------------------

**Pi19256T0**  ----------KIIRE--KCE-KLS---------QIR-MFIREITLM--GAL-KH------ER---IVEFIGVA---------------------------

**Pr72884**  ----------KIMRE--KCE-KLS---------QIR-MFVREITLM--GSL-KH------ER---IVEFIGVA---------------------------

**Ps140986**  ----------KIMRE--KCE-KLS---------QIR-MFIREITLM--GSL-KH------ER---IVEFIGVA---------------------------

**Pi00643T0**  ----------KIRNE--RGV-QRE---------QVE-QFVGEISLI--SGL-NH------PR---IVEFIGAC---------------------------

**Pr72888**  ----------KIRNE--RGV-GRE---------QVE-QFVREISLI--SGL-NH------PR---IVEFVGAC---------------------------

**Ps140988**  ----------KIRNE--RDV-ERE---------QVE-QFVREISLI--SGL-NH------PR---IVEFIGAC---------------------------

**Pi00646T0**  ----------RIRTD--LCQ-DMK---------HVE-AFMKEVCLI--STL-EH------PR---IVEFIGVA---------------------------

**Ps140991**  ----------RIRTD--LCQ-DMK---------HVE-AFMKEICLI--STL-EH------PR---IVEFIGVA---------------------------

**Pr72890**  ----------RIRTD--LCQ-DMK---------HVE-AFMKEICLI--STL-EH------PR---VVEFIGVA---------------------------

**Pi17831T0**  ----------KIRPD--RSG-EVS---------EIE-TFLKEIILM--AVL-YH------PR---IVEFIGVA---------------------------

**Ps132644**  ----------KIRPD--RSG-DVS---------EIE-VFLKEIIMM--AVL-YH------PR---IVEFIGVT---------------------------

**Pi17840T0**  ----------KIRPD--RSV-TTA---------EIE-TFLKEIIMM--AVL-YH------PR---IVKFIGVA---------------------------

**Pr76005**  ----------KIRPD--RSA-TAA---------EIE-LFLKEIIMM--AVL-YH------PR---VVEFVGVA---------------------------

**Ps132640**  ----------KIRPD--RSA-SAA---------EIE-QFLKEIIMM--AVL-YH------PR---IVEFVGVA---------------------------

**Pi17832T0**  ----------KIRPD--RSQ-DPK---------DVK-AFLKEIILM--AEL-HH------PR---IVEFIGVA---------------------------

**Pr76010**  ----------RIRPD--RNQ-DPK---------DVK-AFIKEIALM--AEL-HH------PR---IVEFVGVA---------------------------

**Ps132643**  ----------KIRPD--RSL-DPK---------DVK-AFLKEIILM--AEL-HH------PR---IVEFVGVA---------------------------

**Pi05112T0**  ----------RLLND--K-K-SMQ---------DAL-AFATEIKTM--ARL-DH------PK---IVHFIGVS---------------------------

**Pr82847**  ----------RLLND--K-K-SMQ---------DAL-AFATEIKTM--ARM-DH------PK---IVHFIGVS---------------------------

**Ps132270**  ----------RLLGD--K-K-TMQ---------DAL-AFATEIKTM--ARL-EH------PK---IVRFIGVS---------------------------

**Pu14970**  ----------RLLSD--K-K-SMD---------DAL-AFASEIRIM--ARL-EH------PK---IVRFIGVS---------------------------

**S05981T0**  ----------RLLPE--K-R-TWD---------DAM-NFAMEIKMM--TRL-NH------PK---IVEFIGAA---------------------------

**Pr81631**  ----------RLIQE--Q-V-SLA---------SSE-DFLREVKLM--AWL-EH------PK---IVQFVGVA---------------------------

**Pi02124T0**  ----------KLITV------EGT---------SVQ-DFVSELNLL--ASL-SH------RC---ILSLIGAC---------------------------

**Pr73421**  ----------KLITV------EGT---------SVQ-DFVSELNLL--ASL-SH------RC---ILTLIGAC---------------------------

**Ps144068**  ----------KLITV------EGT---------SVQ-DFVSELNLL--ASL-SH------RC---ILTLIGAC---------------------------

**S04928T0**  ----------KMVQTAPDAD-RQE---------ALC-NLVAEANLL--LTL-RH------AH---IIQLIGVV---------------------------

**Pi07724T0**  ----------RLRPD--R-R-TKK---------HTA-MFIEEIKLI--AQF-DH------PS---LVNFVGAA---------------------------

**Ps158080**  ----------RLRPE--R-R-TKK---------HTI-MFIEEIKLV--ANF-DH------PN---LVNFVGAA---------------------------

**Pr77475A**  ----------RLRPE--R-R-TKR---------HTA-TFIEEIKLI--AQF-DH------PN---LVNFVGAA---------------------------

**Pi07725T0**  ----------RLRPE--R-R-TKK---------HTS-NFVEEIKLI--ANF-DH------PN---LVKLIGAA---------------------------

**Pi21299T0**  ----------RLRPE--R-R-TKK---------HTS-NFVEEIKLI--ANF-DH------PN---LVKLIGAA---------------------------

**Pr77476**  ----------RLRPE--R-R-TKK---------HTS-SFVEEIKLL--ANF-DH------PN---LVKLIGAA---------------------------

**Ps140003**  ----------RLRPE--R-R-TKK---------HTS-NFVEEIKLI--ANF-EH------PN---LVKFIGAA---------------------------

**Pi07731T0**  ----------RLRPE--R-R-TKK---------HTA-AFVEEIKLI--ANF-DH------PN---LVRLIGAA---------------------------

**Ps139996**  ----------RLKPE--R-R-TKK---------HTA-AFVEEIKLI--ARF-DH------PN---LVKLIGAA---------------------------

**Ps139997**  ----------RLKPE--R-R-TKK---------HTA-AFVEEIKLI--ARF-DH------PN---LVKLIGAA---------------------------

**Pr77470**  ----------RLRPE--R-R-TKK---------HTA-AFVEEIKLI--SNF-DH------PN---LVKLIGAA---------------------------

**Pr77475B**  ----------RMRPE--L-R-SKQ---------RTS-AFVEEIKLV--ASF-EH------PN---LVKFIGAA---------------------------

**Pi09664T0**  ----------RLRSD--L-V-TKQ---------RTQ-AFIEEIKMV--APL-DH------PN---VVRLLGCA---------------------------

**Pr75814**  ----------RLRSD--Q-V-TKQ---------RTQ-AFIEEIKMV--APF-DH------PN---VVKLVGCA---------------------------

**Ps135332**  ----------RLRSD--Q-V-TKQ---------RTQ-AFIEEIKMV--APF-DH------PN---IVRLVGCA---------------------------

**Ps135334**  ----------RLRSD--Q-V-TKQ---------RTQ-AFIEEIKMV--APF-DH------PN---IVRLVGCA---------------------------

**Pi23090T0**  ----------RLRED--M-A-TRE---------RVQ-DFVDEIKMV--SKF-NH------PS---IVKFIGAA---------------------------

**Pi23143T0**  ----------RLRED--M-A-TRE---------RVQ-DFVDEIKMV--SKF-NH------PS---IVKFIGAA---------------------------

**Pr81229**  ----------RLRED--M-T-TRE---------RVH-DFVAEIKMV--SRF-DH------PN---IVKFVGAA---------------------------

**Pr75791**  ----------RILND--T-V-ITE---------RTQ-AFVEEIKLV--SKL-DH------PN---IVALIGAA---------------------------

**Pi09665T0**  ----------RLKKG--D-A-TFE---------NTQ-NFVAEIKLC--ATL-DH------PR---VVQLLGVA---------------------------

**Ps135333**  ----------RLKKG--E-A-TYE---------NTQ-SFIAEIKLC--ATL-DH------PR---VVQLLGVA---------------------------

**Ps127796**  ----------RLRKD--L-A-RRR---------ITK-AFIEEIKLA--APL-DH------PS---VVKLVGIA---------------------------

**Pi13397T0**  ----------KILPE--KAA-DDR---------CLS-AFIEEIKLI--SSL-SH------AK---IVRFIGVS---------------------------

**Ps140146**  ----------KILPE--KAA-DDR---------CLS-AFIEEIKLI--SSL-SH------AK---IVRFIGVS---------------------------

**Pr81472**  ----------KILPE--KAA-DDR---------CLS-AFIDEIKLI--SSL-SH------AK---VVRFIGVS---------------------------

**Pi14970T0**  ----------QILPK---AM-SKD---------LLR-RFMDEIRLF--ARM-DH------PK---IVHFIGLA---------------------------

**Ps138087B**  ----------QILPK---AM-SKD---------LLR-RFMDEIRLF--ARM-DH------PK---IVHFIGLA---------------------------

**Pr75692**  ----------QILPK---AM-SKD---------LLR-RFMDEIRLY--ARM-DH------PK---IVRFIGLA---------------------------

**Pi14971T0**  ----------QLLPE--RAR-DPK---------MLN-DFMDEIRTC--ASL-DH------PK---IVRFLGFT---------------------------

**Pi14972T0**  ----------QLLPE--RAR-DKR---------WLG-SFMDEIRLC--STL-DH------PK---IVRFIGVT---------------------------

**Pr75691A**  ----------QLLPE--RAR-DKR---------WLI-SFMEEIRIC--STL-DH------PK---IVHFIGVT---------------------------

**Ps138088**  ----------QLLPE--RAR-DKR---------YLA-GFMEEVRIC--STL-DH------PK---IVRFIGVT---------------------------

**S06057T0**  ----------RMLPS--AVEGNSD---------GVR-DFMDEIRLC--AQL-DH------PH---IVRFIGVS---------------------------

**S02178T0**  ----------RVLPQ--FAD-DVG---------RIE-DFMVEIDLG--ARL-CH------PK---IVTFVGIT---------------------------

**S02311T0**  ----------RMLPE--RLR-SLR---------SIE-DFMEEIRFC--IRL-QH------PH---IVEFIGYC---------------------------

**S02316T0**  ----------RLLPS--HLS-DLR---------CMD-DFMDEIRFS--ARL-RH------DN---IVRFYGFS---------------------------

**S13911T0**  ----------RLRPE--RAD-DTA---------AVE-AFMDEIRLM--SQL-EH------PH---VVGFLGVT---------------------------

**Pi05340T0**  ----------RVAPE--KSK-SVR---------ELQ-RFTREIRLY--GSL-KH------PK---IVAFLGIS---------------------------

**Ps133283**  ----------QVAPE--KAK-SVR---------ELQ-RFTREIRLY--ASL-NH------PK---IVAFRGIS---------------------------

**Ps133286**  ----------QVAPE--KAK-SVR---------ELQ-RFTREIRLY--ASL-NH------PK---IVAFRGIS---------------------------

**Pr80778**  ----------QVAPE--KSK-SVR---------ELQ-RFTREIRLY--AIL-KH------PK---IVAFRGIA---------------------------

**S15848T0**  ----------ELQAV--HAR-QSI---------YIQ-AFMHEIKMF--STL-NH------DN---IVAFVGVS---------------------------

**S15849T0**  ----------ELQAV--HTR-HAT---------HVQ-AFMHEIQLF--STL-QH------DN---IVRFVGVS---------------------------

**S15852T0**  ----------ELQAV--HTR-HAT---------HVQ-AFMHEIQLF--STL-QH------DN---IVRFVGVS---------------------------

**S01258T0**  ----------RNKDK------LEK---------GLQ-SFIAEITLM--AKM-DS------PY---IVRLVGAS---------------------------

**S09257T0**  ----------RMKNQ------EAR---------MVQ-KFIDEIVLM--SQM-NS------DY---IVKFVGAS---------------------------

**S15543T0**  ----------RMKNQ------EAR---------MVQ-KFIDEIVLM--SQM-NS------DY---IVKFVGAS---------------------------

**S09298T0**  ----------RIKDR------RIE---------SVK-KFIDEIKLM--AMM-ES------EF---VVKFVGVS---------------------------

**S15453T0**  ----------RIKDR------RIE---------SVK-KFIDEIKLM--AMM-ES------EF---VVKFVGVS---------------------------

**S08727T0**  ----------RIKHD------STR---------HIQ-KFMDEILLL--SKM-QC------SY---IVEFVGAS---------------------------

**S09308T0**  ----------RIHDK------SSE---------SLA-MFVEEILLR--ATI-ES------PY---IVQFIGVS---------------------------

**S01416T0**  ----------KLLGHRV----GIT---------EVQ-GLVDEIKLM--ASF-DC------PY---IVKCLGAC---------------------------

**S06128T0**  ----------MLLPTRS----SAV---------DVQ-GLVDEIKLM--AQF-DS------PH---IVSLIGAT---------------------------

**S12836T0**  ----------KLLPHKA----DVE---------DAI-QFIDEVKLM--AEF-KS------PY---IVALRGAA---------------------------

**S17043T0**  ----------RLLPNKS----SPK---------DIL-NFINECKLM--ARF-ES------PY---VVKLYGVS---------------------------

**S08987T0**  ----------SLLPGKH----SQA---------DVT-TFVAEIQLA--SMM-ES------PY---IVKLLGAS---------------------------

**S13082T0**  ----------RLLSGAT----SQS---------AIA-TFVDEILLA--SKL-ES------LY---VVQLIGAS---------------------------

**S05673T0**  ----------RLREDNA----TIK---------NIE-TFIQEIELM--SRF-DS------PF---LVQFVGAS---------------------------

**S09620T0**  ----------RLREDNA----TIK---------NIE-TFIQEIELM--SRF-DS------PF---LVQFVGAS---------------------------

**S12826T0**  ----------KPMPSFL----SAT---------NMR-RMIGEISLL--SRL-SS------PY---VVTLLGAC---------------------------

**S03267T0**  ----------ILLPE--K-R-SQS---------DIA-AFLREITLL--ASL-DH------PR---IVRCIGAA---------------------------

**S13910T0**  ----------SLPPD--KSD-DPD---------AVG-IFLETVRLH--AQL-HH------PK---IVRFVGVT---------------------------

**Pi05338T0**  ----------KVSVH-SQGQ-DRD---------QMV-AFMDEIRVY--AKL-EH------PK---VVGFLGIM---------------------------

**Pr80780**  ----------KVSVH-NKGQ-DRD---------QMI-AFMDEIRVC--AKL-DH------PK---VVGFLGIM---------------------------

**Ps133281**  ----------KVTVH-TQGQ-DRD---------QMI-AFMDEIRVC--AKL-EH------PK---VVAFLGIM---------------------------

**Pi22892T0**  ----------RLQQK--DLG-ETS---------EMQ-HFAEEIHLA--ASL-DH------PR---IVALVGVI---------------------------

**Pr94275**  ----------RLQQE--AVD-EAI---------EME-RFVEEVQIS--ASL-DH------PR---IVALVGVL---------------------------

**Ps135323**  ----------RLQQE--SID-EAS---------ETQ-RFAQEVRLA--ATL-DH------PR---IVALVGII---------------------------

**S01930T0**  ----------KLALH--VTD-VEG---------CAS-ALYDELKAL--ARL-SH------PN---ILRVVGVL---------------------------

**Ps133943**  ----------YITPKEDQGPRSSS---NGNGGGARD-KLKGELKRQ--AIF-SH------PQ---VVAFIGVA---------------------------

**S03896T0**  ----------TLMTDTHAAGALA---------AQAD-AFLAVITLR--ARL-DH------PN---IVTFIGAV---------------------------

**S09249T0**  ----------YLFTSSSTT-------------SEMQ-TGIQQVLAA--TRL-DS------RY---VLALHGVS---------------------------

**Pi03775T0**  ----------KIKSNTASK-------------ALMR-DFVTEIELM--FEL-KH------PR---IAAFRGAM---------------------------

**Pr75312**  ----------KIKSNTASK-------------ALMR-DFVTEIELM--FEL-KH------PR---IAAFRGAM---------------------------

**Ps136506**  ----------KIKSNTASK-------------ALMR-DFVTEIELM--FEL-KH------PR---IAAFRGAM---------------------------

**AT3G14840_LRRXIV**  ----------RISLE--ST-------------KNNE-AYLTELDFF--SRF-AH------PR---IIPFVGKS---------------------------

**Ps141628**  ----------TLSIA--SRGGRNGDKRDNTLLAELW-GFVEEIRLS--ATL-SH------PQ---LVAFYGFV-----KMT-----------RFAE----

**Pu08017**  ----------HLSC-------RDKNVSEVKRVASLR-CFLQEIHLN--TMV-SH------PQ---IVTFLGYFNDELPSTT-----------LSAEDLSA

**S14923T0**  ----------RVHAQEVGD-------------AKIQ-TFVADVNAI--AQL-KH------PH---MLRLYGIA---------------------------

**S14924T0**  ----------RVDARIISE-------------DDVA-TFVSDVNQL--AQL-VH------PQ---LVALHGIV---------------------------

**Mb37167**  ----------CLKNTTQVLD-----------AVAVQ-EFAREIRTL--RLM-RH------RN---IVFFYGAC---------------------------

**AtAME2**  ----------VIRSIN----------------KYRE-AAMIEIDVL--QRL-TR-HDVGGSR---CVQIRNWF---------------------------

**hCLK1**  ----------IVKNVD----------------RYCE-AARSEIQVL--EHL-NTTDPNSTFR---CVQMLEWF---------------------------

**AtCDC2a**  ----------KIRLEQEDE-------------GVPS-TAIREISLL--KEM-QH------SN---IVKLQDVV---------------------------

**hCDK3**  ----------KIRLDLEME-------------GVPS-TAIREISLL--KEL-KH------PN---IVRLLDVV---------------------------

**AtMPK1**  ----------KIHNVYENR-------------IDAL-RTLRELKLL--RHL-RH------EN---VIALKDVM---------------------------

**hMAPK1**  ----------KISPFEHQ--------------TYCQ-RTLREIKIL--LRF-RH------EN---IIGINDII---------------------------

**AtGSK3b**  ----------KVLQDRR--------------------YKNRELQLM--RLM-DH------PN---VVSLKHCF---------------------------

**AtGSK3i**  ----------KVLQDRR--------------------YKNRELQLM--RPM-DH------PN---VISLKHCF---------------------------

**AtCKA1**  ----------ILKPVKKK-------------------KIRREIKIL--QNLCGG------PN---IVKLLDVV---------------------------

**hCK2a**  ----------ILKPVKKK-------------------KIKREIKIL--ENLRGG------PN---IITLADIV---------------------------

**AtMEKK1**  ----------EVSLLDQGSQAQ----------ECIQ-QLEGEIKLL--SQL-QH------QN---IVRYRGTA---------------------------

**hMAPKKK1**  ----------QVTYVRNTSSEQE---------EVVE-ALREEIRMM--SHL-NH------PN---IIRMLGAT---------------------------

**AtMKK3**  ----------KINIFER---------------EKRQ-QLLTEIRTL--CEAPCH------EG---LVDFHGAF---------------------------

**hMAPKK1**  ----------LIHLEIKP--------------AIRN-QIIRELQVL--HEC-NS------PY---IVGFYGAF---------------------------

**AtCPK7**  ----------SISKKKLRTA------------VDIE-DVRREVEIM--KHMPKH------PN---VVSLKDSF---------------------------

**hCaMK1**  ----------CIAKEALE--------------GKEG-SMENEIAVL--HKI-KH------PN---IVALDDIY---------------------------

**AtNPH1**  ----------AMDKAVMLNR------------NKVH-RARAEREIL--DLL-DH------PF---LPALYASF---------------------------

**AtPVPKlikePK5**  ----------VMDKTSLASR------------KKLL-RAQTEREIL--QSL-DH------PF---LPTLYTHF---------------------------

**AtS6KlikePK1**  ----------VMRKDHIMEK------------NHAE-YMKAERDIL--TKI-DH------PF---IVQLKYSF---------------------------

**hGRK6**  ----------KLEKKRIKKR------------KGEA-MALNEKQIL--EKV-NS------RF---VVSLAYAY---------------------------

**AtSnRK2**  ----------YIERGE----------------KIDE-NVQREIINH--RSL-RH------PN---IVRFKEVI---------------------------

**OUTGROUP1**  ----------VLHQDLHLQP------------GMVE-RFKREAQLL--SAV-EH------PA---VVRIIDFG---------------------------

**OUTGROUP2**  ----------MLRYELSKDE------------RFIK-RFKRESSQV--INL-DH------PN---IVHVYNVG---------------------------

**OUTGROUP3**  ----------TFKIDANDE-------------DAVK-RFNREAKAV--TSL-SH------PN---IVSIYDVE---------------------------

**OUTGROUP4**  ----------VLRADLARDP------------SFYL-RFRREAQNA--AAL-NH------PA---IVAVYDTG---------------------------

**AtTousled**  ----------LHGLNAQWSEEKKQ--------SYIR-HANRECEIH--KSL-VH------HH---IVRLWDKF---------------------------

**hTousledLK1**  ----------IHQLNKSWRDEKKE--------NYHK-HACREYRIH--KEL-DH------PR---IVKLYDYF---------------------------

**S08428T0**  ----------VAADT-----------------AAVT-ATVHEVVLL--SAI-DH------PH---IVRLVGFV---------------------------

**S08432T0**  ----------MLTPCAVQDTT-----------MRLG-GLVHGIAFL--ARL-SH------PN---IVPLLGFA---------------------------

**Pi03773T0**  ----------KLQPSD----------------DDVE-AALDMVPTL--SQM-RH------PQ---LLSITGLV---------------------------

**Pr75310**  ----------KLQTSD----------------DDVE-AALDMVPTL--SQM-RH------PQ---LLSITGLV---------------------------

**Ps136501**  ----------KLQTSD----------------DDVE-AALDMVPTL--SQM-RH------PQ---ILSITGLV---------------------------

**Pu07161**  ----------QLKLKEDAA-------------ENCE-MVLDVVPVV--SQL-RH------PQ---LLSVIGLM---------------------------

**S09505T0**  ----------RVLPLTQD-------------------KALSLLSML--STL-RH------PR---LMNVLGVV---------------------------

**AT1G14390_LRRVI-1**  ----------CIKLKQK---------------NSTQ-NLAQQMEVL--SKL-RH------MH---LVSVLGHC---------------------------

**AT5G41180_LRRVI-2**  ----------SLCVKEENWT------------GYLELNFQREVAAL--ARL-NH------EN---AGKLLGYC---------------------------

**hTGFbRI**  ----------IFSSR------------------EER-SWFREAEIYQTVML-RH------EN---ILGFIAAD---------------------------

**hTGFbRII**  ----------IFPYE------------------EYA-SWKTEKDIFSDINL-KH------EN---ILQFLTAE---------------------------

**Pi14992T0**  ----------RMMTLRFEV-------------KELA-DTIKDVELV--GSL-HH------PN---IVTCLGTM---------------------------

**Ps157753**  ------------------------------------------------YGM---------SK--------------------------------------

**Pi04971T0**  ----------ALMRSK----------------KRLETRFVEQIRLA--AAL-DH------AS---IVHFIGVTTGCSTAASRRRGSSAAAPAQGPYNSYG

**Ps136072**  ----------ALMRSK----------------KRLEKHFVEQIRLA--ASF-DH------AS---IVHFIGVTTGCSTTASRRRGSSAVASPH-PYNANG

**Pr93648**  ----------ALMRSK----------------KRLEKRFVEQIRLA--ASL-DH------AS---IVHFIGVTTGCSTTASRRRGSSAASPQP----PNG

**Pu03658**  ----------------------------------------------------------------------------------------------------

**AtCKI1**  -----------LEPVKTRHP-----------------QLQYESKIY--MFL-QG------GTGVPHLKWFGVE---------------------------

**hCKIalpha2**  -----------LESQKARHP-----------------QLLYESKLY--KIL-QG------GVGIPHIRWYGQE---------------------------

**mCKIalpha**  -----------LESQKARHP-----------------QLLYESKLY--KIL-QG------GVGIPHIRWYGQE---------------------------

**V**

**------------**

**210 220 230 240 250 260 270 280 290 300**

**....|....|....|....|....|....|....|....|....|....|....|....|....|....|....|....|....|....|....|....|**

**AT1G06840_LRRVIII-1** -DEE---------------------GEQ--MLVYEYMENGTLRDNIS-----------------------------------------------------

**AT4G29180_LRRI**  -DDG---------------------RSM--ALIYEYMANGNLQDYLS-----------------------------------------------------

**AT3G14840_LRRVIII-2** -VEG---------------------DQL--LLVYEYLENNSLARALF-----------------------------------------------------

**AtNIK1_LRRII**  -ITQ---------------------TEK--LLVYPYMSNGSVASRMK-----------------------------------------------------

**AtNAK**  -LEE---------------------EHR--LLVYEFMTRGSLENHLF-----------------------------------------------------

**AT2G24230_LRRVII**  -IAG---------------------DQR--IAIYEYMENGNLQNLLH-----------------------------------------------------

**AtBRI1_LRRXb**  -KVG---------------------DER--LLVYEFMKYGSLEDVLH-----------------------------------------------------

**AtCLV1_LRRXI**  -ANK---------------------DTN--LLLYEYMPNGSLGELLH-----------------------------------------------------

**AtFEI1_LRRXIIIa**  -NSP---------------------TSK--LLLYDYLPGGSLDEALH-----------------------------------------------------

**AtER_LRRXIIIb**  -LSH---------------------LGS--LLFYDYLENGSLWDLLH-----------------------------------------------------

**AtRPK1_LRRXV**  -ASE---------------------TEM--FLIYNYLSGGNLQDFIK-----------------------------------------------------

**AtTMK1_LRRIX**  -LDG---------------------NEK--LLVYEYMPQGTLSRHLF-----------------------------------------------------

**AT3G28450_LRRXa**  -VVE---------------------EEK--FLVYKYMSNGTLHSLLD-----------------------------------------------------

**AtIMK3_LRRIII**  -VKK---------------------REK--LVVFDYMSRGSLATFLH-----------------------------------------------------

**AT2G45340_LRRIV**  -CSKGR-------------------GEC--FLIYEFVPNGNLLQYLD-----------------------------------------------------

**AtSCM_SUB_LRRV**  -NEF---------------------GQR--LLVYEYCPNGSLQDALH-----------------------------------------------------

**AtFLS2_LRRXII**  -WES---------------G-----KTK--ALVLPFMENGNLEDTIH-----------------------------------------------------

**dmPELLE**  -IKG---------------------GKP--CLVYQLMKGGSLEARLR-----------------------------------------------------

**drIRAK1**  -I-S---------------G-----QTY--CVIYVYMPNGSLDDRLR-----------------------------------------------------

**hIRAK1**  -A-Q---------------N-----GFY--CLVYGFLPNGSLEDRLH-----------------------------------------------------

**xtPELLE**  -F-Q---------------G-----EEY--CLIYLYLPNGSLEDRLH-----------------------------------------------------

**AtCTR1_Raf**  -T-Q---------------P-----PNL--SIVTEYLSRGSLYRLLH-----------------------------------------------------

**Mb27170**  -V-H---------------E-----EQS--FLVLEYMGRGSLTKTLD-----------------------------------------------------

**Mb28586**  -QEPE--------------K-----GSP--FLVTEYMELGSLSTVLK-----------------------------------------------------

**hRaf1**  -T-----------------K-----DNL--AIVTQWCEGSSLYKHLH-----------------------------------------------------

**Mb37485**  -E-DP--------------D-----QRP--FLVTEFCERGSLRSLLV-----------------------------------------------------

**Esi0009_0077**  -T-SL--------------P-----DRL--VLVMELLQGGDLRHRLK-----------------------------------------------------

**Esi0009_0083**  -T-SL--------------P-----DRL--VLVMELLQGGDLRHRLK-----------------------------------------------------

**Esi0020_0071**  -T-SQ--------------P-----DHM--VLVMELLSGGDLRTLLR-----------------------------------------------------

**Esi0173_0029**  -T-SL--------------P-----DRL--ILVLELLPGGDLRTMLK-----------------------------------------------------

**Ch136568**  -F-K---------------D-----QRT--LLVTEYMEGGNLTHNLR-----------------------------------------------------

**Ch50123**  -V-L---------------D-----SRL--VLVTEYMEGGNLTQNLA-----------------------------------------------------

**Ch137597**  -LRG---------------------SLL--MLAMELMRGGSLRVALQ-----------------------------------------------------

**Ch137605**  -LKG---------------------CLL--MLAMRLMEGGSLRAALQ-----------------------------------------------------

**Ch56654**  -LKG---------------------CLL--MLAMRLMEGGSLRAALQ-----------------------------------------------------

**Ch141610**  -VKG---------------------RMV--MLAMKLMRGGTLRAALQ-----------------------------------------------------

**Ch143410**  -LKG---------------------QIV--LLAMDLMKGGTLRAALQ-----------------------------------------------------

**Ch141452**  -IVN---------------------SLL--MMAMGLMEGGTLRTALK-----------------------------------------------------

**Mb10450**  -------------------ETNWSPPAP--FIVLELVERGALNDVLN-----------------------------------------------------

**Mb25375**  -----------------EGDFGAQRQIP--FLVLELVKQGSLRSVLR-----------------------------------------------------

**Mb28923**  -----------------EGAFDGQAGVP--FLVLELVSLGSLRSVLR-----------------------------------------------------

**Mb37923**  -----------------YGPIHGAGETS--FLVLELLERGSLKSVLR-----------------------------------------------------

**Mb34096**  -----------------HMPADGT-DVP--FLVLELVDRGSLAQVLL-----------------------------------------------------

**Mb36839**  -TLS---------------S-----GCP--FLVLELVELGSYQKFLR-----------------------------------------------------

**Mb36426**  -YMD---------------------DRP--ALVLEFVELGSLDRLCR-----------------------------------------------------

**hAXL**  -F-QG--------------SERESFPAP--VVILPFMKHGDLHSFLLYSR--------------------------------------------------

**hHGFR**  -LRS---------------E-----GSP--LVVLPYMKHGDLRNFIRNETHN------------------------------------------------

**hRYK**  -I-E-E-------------G-----EKP--MVILPYMNWGNLKLFLRQCKL-------------------------------------------------

**hDDR**  -V-Q---------------D-----DPL--CMITDYMENGDLNQFLSAHQL---------EDKAAEGA--------------------------------

**hIR**  -S-K---------------G-----QPT--LVVMELMAHGDLKSYLRSLRP---------EAENNPG---------------------------------

**hLTK**  -L-R---------------A-----TPR--LILLELMSGGDMKSFLRHSRP-------------------------------------------------

**hMuSK**  -A-V---------------G-----KPM--CLLFEYMAYGDLNEFLRSMSPHTVCSLSHSDLSMRAQV--------------------------------

**hTRKalpha**  -T-E---------------G-----RPL--LMVFEYMRHGDLNRFLRSHGP---------DAKLLAGG--------------------------------

**hKLGlikePTK7**  -R-E---------------A-----EPH--YMVLEYVDLGDLKQFLRISKS---------KDEKL-----------------------------------

**hFGFR2**  -T-Q---------------D-----GPL--YVIVEYASKGNLREYLRARR------------------PPGMEY--------------------------

**hRET**  -S-Q---------------D-----GPL--LLIVEYAKYGSLRGFLRESR------------------KVGPGY--------------------------

**hVGFR1**  -TKQ---------------G-----GPL--MVIVEYCKYGNLSNYLKSKRDLFFLNKDAALHMEPKKEKMEPGLEQGKKPRLDSVTSSES----------

**hPDGFRbeta**  -T-K---------------G-----GPI--YIITEYCRYGDLVDYLHRNKHT-FLQHHSDKRRPPSAELYSNALPVGLPLPSHVSLTGESDGGYMDMSKD

**hTIE1**  -K-N---------------R-----GYL--YIAIEYAPYGNLLDFLRKSR------------------VLETDP--------------------------

**hEGFR**  -L-T---------------------STV--QLITQLMPFGCLLDYVREHKDN------------------------------------------------

**hEPH**  -TKR---------------------KPI--MIITEFMENGALDAFLREREDQ------------------------------------------------

**Pi00640T0**  -W-DS--------------L-----RNL--SAVTEYMERGDLRDVLH-----------------------------------------------------

**Pi19256T0**  -W-DS--------------L-----RNL--SAVTEYMERGDLRDVLH-----------------------------------------------------

**Pr72884**  -W-DS--------------L-----RNL--SAITEYMERGDLRDVLH-----------------------------------------------------

**Ps140986**  -W-DS--------------L-----RNL--SAVTEYMERGDLRDVLH-----------------------------------------------------

**Pi00643T0**  -W-TS--------------P-----AEL--SAVTELMERGDLRDVTR-----------------------------------------------------

**Pr72888**  -W-TT--------------P-----AEL--SAVTELMERGDLRDVTR-----------------------------------------------------

**Ps140988**  -W-TT--------------P-----AEL--SAVTELMERGDLRDVTR-----------------------------------------------------

**Pi00646T0**  -W-DT--------------L-----RNL--SAVTEYMERGDLREVLR-----------------------------------------------------

**Ps140991**  -W-DT--------------L-----RNL--SAVTEYMERGDLREVLR-----------------------------------------------------

**Pr72890**  -W-DT--------------L-----RNL--SAVTEYMERGDLREVLH-----------------------------------------------------

**Pi17831T0**  -W-DD--------------I-----RHL--SAVTEYMNKGDLR---------------------------------------------------------

**Ps132644**  -W-DN--------------L-----RHL--AAVTEFMDNGDLREVLY-----------------------------------------------------

**Pi17840T0**  -W-DN--------------L-----RHL--SAVTEFMDNGDLREVLH-----------------------------------------------------

**Pr76005**  -W-DN--------------L-----RHL--SAVTEFMDNGDLRDVLH-----------------------------------------------------

**Ps132640**  -W-DN--------------L-----RHL--SAVTEFMDNGDLRDVLH-----------------------------------------------------

**Pi17832T0**  -W-DN--------------L-----KHI--SAVTEFMESGDLRHVLR-----------------------------------------------------

**Pr76010**  -W-DN--------------L-----RHI--SAVSEFMESGDLRHVLR-----------------------------------------------------

**Ps132643**  -W-DN--------------L-----KHL--SAVSEFMEGGDLRHVLR-----------------------------------------------------

**Pi05112T0**  -W-TN--------------A-----LTI--QAVTEFMDCGDLKSLLD-----------------------------------------------------

**Pr82847**  -W-TN--------------A-----LTI--QAVTEFMDCGDLKSLLD-----------------------------------------------------

**Ps132270**  -W-SN--------------A-----LTI--QAVTEFMDCGDLKSLLD-----------------------------------------------------

**Pu14970**  -W-TN--------------Y-----LTI--QAVTEFMDCGDLKSLLD-----------------------------------------------------

**S05981T0**  -W-SN--------------A-----LSI--QAICEYMNCGDLKSLLD-----------------------------------------------------

**Pr81631**  -W-TK--------------L-----VDM--LAVIEYMDGGDLRTLLD-----------------------------------------------------

**Pi02124T0**  -W-DDE-------------L-----TDI--QIVMEYMDSGDLLSVLR-----------------------------------------------------

**Pr73421**  -W-DEE-------------L-----TDI--QIVMEYMDSGDLLSVLR-----------------------------------------------------

**Ps144068**  -W-DED-------------L-----TDI--QIVLEYMDSGDLLSVLR-----------------------------------------------------

**S04928T0**  -T-DR--------------V-----NEI--AVVLEYMDLGDLREMLM-----------------------------------------------------

**Pi07724T0**  -W-TI--------------E-----SDL--QMLLEYMDGGDLRRYLA-----------------------------------------------------

**Ps158080**  -W-TI--------------E-----SDL--QMLLEYMEGGDLRRYLS-----------------------------------------------------

**Pr77475A**  -W-TI--------------E-----SDL--QALMEYMEGGDLRQYLA-----------------------------------------------------

**Pi07725T0**  -W-TI--------------E-----SDL--QALLEYMDGGDLRQYLS-----------------------------------------------------

**Pi21299T0**  -W-TI--------------E-----RDL--QALLEYMDGGDLRQYLS-----------------------------------------------------

**Pr77476**  -W-TI--------------E-----SDL--QALLEYMDGGDLRQYLG-----------------------------------------------------

**Ps140003**  -W-TI--------------E-----SDL--QALLEYMDGGDLRHYLA-----------------------------------------------------

**Pi07731T0**  -W-TV--------------E-----TDL--QMLLEYMEGGDLRRYLA-----------------------------------------------------

**Ps139996**  -W-TV--------------E-----TDL--QMLLEYMEAGDLRRYLA-----------------------------------------------------

**Ps139997**  -W-TV--------------E-----TDL--QMLLEYMEAGDLRRYLA-----------------------------------------------------

**Pr77470**  -W-TV--------------E-----TDL--QMLLEYMEGGDLRRYLA-----------------------------------------------------

**Pr77475B**  -W-TI--------------E-----SDL--QALLEYMDGGDLRDYLA-----------------------------------------------------

**Pi09664T0**  -W-TI--------------E-----SDL--QALFEYMEGGDLRDYLV-----------------------------------------------------

**Pr75814**  -W-TI--------------E-----SDL--QALFEYMENGDLRDYLM-----------------------------------------------------

**Ps135332**  -W-TI--------------E-----TDL--QALFEYMENGDLRDYLV-----------------------------------------------------

**Ps135334**  -W-TI--------------E-----TDL--QALFEYMENGDLRDYLV-----------------------------------------------------

**Pi23090T0**  -W-SI--------------E-----SDL--QAIFEYMENGDLRTYLS-----------------------------------------------------

**Pi23143T0**  -W-SI--------------E-----SDL--QAIFEYMENGDLRTYLS-----------------------------------------------------

**Pr81229**  -W-SI--------------Q-----SDL--QALFEYMENGDLRTYLS-----------------------------------------------------

**Pr75791**  -W-SS--------------G-----SDL--QALFEFVENGDLRSYLA-----------------------------------------------------

**Pi09665T0**  -W-TI--------------E-----SDL--QAMFEYMAQGDLRTYLE-----------------------------------------------------

**Ps135333**  -W-TI--------------E-----SDL--QAMFEYMSNGDLRTYLE-----------------------------------------------------

**Ps127796**  -W-TV--------------K-----TDI--QALYEYVDNGNVTDYLT-----------------------------------------------------

**Pi13397T0**  -W-SM--------------L-----SDM--AVLMEYLPNGDLDMLLK-----------------------------------------------------

**Ps140146**  -W-SM--------------L-----SDM--AVLMEYMPNGDLDMLLK-----------------------------------------------------

**Pr81472**  -W-SM--------------L-----SDM--AVLMEYMPNGDLDMLLK-----------------------------------------------------

**Pi14970T0**  -W-TN--------------L-----LDL--SLVVEYMPRGDLTTLIR-----------------------------------------------------

**Ps138087B**  -W-TN--------------L-----MDL--SLIVEYMPRGDLTTLLR-----------------------------------------------------

**Pr75692**  -W-TN--------------L-----MDL--SLILEYMPRGDLTTLVR-----------------------------------------------------

**Pi14971T0**  -F-TS--------------L-----MDL--SVVFEHMPNGDLATLLQ-----------------------------------------------------

**Pi14972T0**  -W-SS--------------L-----SDI--SMVMEYMPRGDLSTMLQ-----------------------------------------------------

**Pr75691A**  -W-SS--------------L-----LDI--NMVMEYMPRGDLSTLLQ-----------------------------------------------------

**Ps138088**  -W-ST--------------M-----LDI--SMVMEYMPRGDLSTMLQ-----------------------------------------------------

**S06057T0**  -W-ST--------------L-----KDL--SAVLEFMPNGDLATLVR-----------------------------------------------------

**S02178T0**  -W-TT--------------L-----YNI--SSLTEYMPNGDVWSLLE-----------------------------------------------------

**S02311T0**  -W-TT--------------L-----PNL--SVVTEYMSEGDLWTLLD-----------------------------------------------------

**S02316T0**  -Y-ST--------------L-----ADL--AIVTEYMERGDLWQWLR-----------------------------------------------------

**S13911T0**  -W-TH--------------L-----HNI--GLLTEYMEHGDLWGLLQ-----------------------------------------------------

**Pi05340T0**  -W-SF--------------L-----ADL--SLVLEYMPNGDLARFLE-----------------------------------------------------

**Ps133283**  -W-SS--------------L-----ADL--SLVIEYMPNGDLAQFLE-----------------------------------------------------

**Ps133286**  -W-SS--------------L-----ADL--SLVIEYMPNGDLAQFLE-----------------------------------------------------

**Pr80778**  -W-SS--------------L-----ADL--SLVIEYMPNGDLAQFLE-----------------------------------------------------

**S15848T0**  -W-TT--------------L-----HDL--ALITEFLPNGDLRELLS-----------------------------------------------------

**S15849T0**  -W-TT--------------L-----HDL--ALVTEFLPNGDLRDLLL-----------------------------------------------------

**S15852T0**  -W-TT--------------L-----HDL--ALVTEFLPNGDLRDLLL-----------------------------------------------------

**S01258T0**  -W-VR--------------P-----VDI--ECVVEFMDLGDLRCFLA-----------------------------------------------------

**S09257T0**  -W-TR--------------P-----IEI--ECVVEYMDLGDLRSYLV-----------------------------------------------------

**S15543T0**  -W-TR--------------P-----IEI--ECVVEYMDLGDLRSYLV-----------------------------------------------------

**S09298T0**  -W-RR--------------P-----IEM--EVVVEYMDLGDLRNYLM-----------------------------------------------------

**S15453T0**  -W-RR--------------P-----IEM--EVVVEYMDLGDLRNYLM-----------------------------------------------------

**S08727T0**  -W-RR--------------P-----MDL--ACVVEYMDLGDLRSFLS-----------------------------------------------------

**S09308T0**  -W-HQ--------------P-----RDM--ECVLEYMNRGNLQDYLA-----------------------------------------------------

**S01416T0**  -W-DQ--------------P-----SDM--KCVLEFMGGGDLRDYLQ-----------------------------------------------------

**S06128T0**  -W-TT--------------P-----SSI--RCVLEYMNMGDLRDYLA-----------------------------------------------------

**S12836T0**  -W-NN--------------P-----VEL--FAVIEYMDKGDLKDYLA-----------------------------------------------------

**S17043T0**  -W-TR--------------P-----VDF--MAVMEFMDRGDLKSYLA-----------------------------------------------------

**S08987T0**  -W-RT--------------P-----SEL--EMVLER----------------------------------------------------------------

**S13082T0**  -W-RI--------------P-----AEL--QMVLEWMDRGDLRSVLE-----------------------------------------------------

**S05673T0**  -W-TH--------------L-----ADM--KCVVEYMEKGDLQTYLE-----------------------------------------------------

**S09620T0**  -W-TH--------------L-----ADM--KCVVEYMEKGDLQTYLE-----------------------------------------------------

**S12826T0**  -W-NR--------------P-----MDL--MMAMEHMDGGNLQGHLA-----------------------------------------------------

**S03267T0**  -W-PKS-------------K-----RDM--MLLTEYVAGGDLRALLD-----------------------------------------------------

**S13910T0**  -W-RT--------------V-----KQL--ALITEYMERGDLWSLLQ-----------------------------------------------------

**Pi05338T0**  -W-AS--------------L-----SDL--SAIVEYVPRGSVGAYLK-----------------------------------------------------

**Pr80780**  -W-AS--------------L-----SDL--SAIVEYVPKGSLAMYLK-----------------------------------------------------

**Ps133281**  -W-AS--------------L-----SDL--SAIVEYIPRGSLAVFLK-----------------------------------------------------

**Pi22892T0**  -W-SR--------------M-----YGL--EALFEYMEGGNLRVYLD-----------------------------------------------------

**Pr94275**  -W-SR--------------V-----YGL--EALYEYMEGGNLRSYLA-----------------------------------------------------

**Ps135323**  -W-SR--------------M-----YGL--EALYEYMEGGDLRSHLA-----------------------------------------------------

**S01930T0**  -L-P---------------P-----ASV--AGVLAYCAHGDLQSLLG-----------------------------------------------------

**Ps133943**  -W-SR--------------E-----THL--VAVTEYMAQGDLRQWLH-----------------------------------------------------

**S03896T0**  -W-SS--------------NLRKGMLGF--GFLSEHMAKGDLGRLLT-----------------------------------------------------

**S09249T0**  -WTT---------------P-----KDL--MLVQEYMDMGDLETYLR-----------------------------------------------------

**Pi03775T0**  -W-DAD-------------G-----TEL--CAVVEYVEKGALRDCTV-----------------------------------------------------

**Pr75312**  -W-DAD-------------G-----TEL--CAVVEYVEKGALRDCTV-----------------------------------------------------

**Ps136506**  -W-DAD-------------G-----TEL--CAVVEYVEKGALRDCTV-----------------------------------------------------

**AT3G14840_LRRXIV**  -L-ES--------------A-----THK--FLVYKYMLNRDLPSSLF-----------------------------------------------------

**Ps141628**  -H-SD--------------S-----EGV--ALVMEYMGKGDLNSFIQ-----------------------------------------------------

**Pu08017**  GN-DS--------------D-----ESL--TLVTECMGKGSLDAFIQ-----------------------------------------------------

**S14923T0**  -R-MN--------------D-----FEL--CVVAELMSMGSLAQVLQ-----------------------------------------------------

**S14924T0**  -RLGD--------------------YEV--GAVAEFMHCGSLPHILM-----------------------------------------------------

**Mb37167**  -N-----------------E-----SPC--LLVMEFVERGSLADIIK-----------------------------------------------------

**AtAME2**  -----------------DYR-----NHI--CIVFEKL-GPSLYDFLR-----------------------------------------------------

**hCLK1**  -----------------EHH-----GHI--CIVFELL-GLSTYDFIK-----------------------------------------------------

**AtCDC2a**  -----------------HSE-----KRL--YLVFEYLDL-DLKKHMD-----------------------------------------------------

**hCDK3**  -----------------HNE-----RKL--YLVFEFLSQ-DLKKYMD-----------------------------------------------------

**AtMPK1**  -----------------MPIHKMSFKDV--YLVYELMD-TDLHQIIK-----------------------------------------------------

**hMAPK1**  -----------------RAPTIEQMKDV--YIVQDLME-TDLYKLLK-----------------------------------------------------

**AtGSK3b**  -----------------FSTTTRDELFL--NLVMEYVPE-TLYRVLK-----------------------------------------------------

**AtGSK3i**  -----------------FSTTSRDELFL--NLVMEYVPE-TLYRVLR-----------------------------------------------------

**AtCKA1**  ---------------RDQHS-----KTP--SLIFEYVNSTDFKVLYP-----------------------------------------------------

**hCK2a**  ---------------KDPVS-----RTP--ALVFEHVNNTDFKQLYQ-----------------------------------------------------

**AtMEKK1**  -----------------KDG-----SNL--YIFLELVTQGSLLKLYQ-----------------------------------------------------

**hMAPKKK1**  -----------------CEK-----SNY--NLFIEWMAGGSVAHLLS-----------------------------------------------------

**AtMKK3**  ---------------YSPDS-----GQI--SIALEYMNGGSLADILK-----------------------------------------------------

**hMAPKK1**  -----------------YSD-----GEI--SICMEHMDGGSLDQVLK-----------------------------------------------------

**AtCPK7**  -----------------EDD-----DAV--HIVMELCEGGELFDRIV-----------------------------------------------------

**hCaMK1**  -----------------ESG-----GHL--YLIMQLVSGGELFDRIV-----------------------------------------------------

**AtNPH1**  -----------------QTK-----THI--CLITDYYPGGELFMLLD-----------------------------------------------------

**AtPVPKlikePK5**  -----------------ETE-----KFS--CLVMEFCPGGDLHTLRQ-----------------------------------------------------

**AtS6KlikePK1**  -----------------QTK-----YRL--YLVLDFINGGHLFFQLY-----------------------------------------------------

**hGRK6**  -----------------ETK-----DAL--CLVLTLMNGGDLKFHIY-----------------------------------------------------

**AtSnRK2**  -----------------LTP-----SHL--AIVMEYAAGGELYERIC-----------------------------------------------------

**OUTGROUP1**  -----------------ESE-----HSA--CLVMELVEGESLNDALQ-----------------------------------------------------

**OUTGROUP2**  -----------------DYK-----QQP--FIVMEYVKGKTLKDYLR-----------------------------------------------------

**OUTGROUP3**  -----------------NEG-----EFY--YLILEYVEGMTLKDYMI-----------------------------------------------------

**OUTGROUP4**  ------------------EAETPAGPLP--YIVMEYVDGVTLRDIVH-----------------------------------------------------

**AtTousled**  ---------------------HIDMHTF--CTVLEYCSGKDLDAVLK-----------------------------------------------------

**hTousledLK1**  ---------------------SLDTDTF--CTVLEYCEGNDLDFYLK-----------------------------------------------------

**S08428T0**  --------------------ASPRLVEM--TIVLEYTQLGSLERVLK-----------------------------------------------------

**S08432T0**  -TSSSL-------------------LDL--TVVTEFMALGSLSDVLRDASLVKY----------------------------------------------

**Pi03773T0**  ---------------------WDDRHAM--TAVCEYMTLGTLEAYLR-----------------------------------------------------

**Pr75310**  ---------------------WDERHAM--TAVCEYMTLGTLEAYLR-----------------------------------------------------

**Ps136501**  ---------------------WDERHAM--TAVCEYMTLGTLEAYLR-----------------------------------------------------

**Pu07161**  ---------------------CEDFHPV--SVVCEAMDQGTLEDYLM-----------------------------------------------------

**S09505T0**  -W-----------------RSVDVGIQM--DVALEFLDSGTLESVLL-----------------------------------------------------

**AT1G14390_LRRVI-1**  -------------IGTYQDHHPYAGSTI--FIVQEYISNGSLRDYLT-----------------------------------------------------

**AT5G41180_LRRVI-2**  -KES---------------------TPFTRMLVFEYASNGTLYDHLH-----------------------------------------------------

**hTGFbRI**  -N-----------------KDNGTWTQL--WLVSDYHEHGSLFDYLN-----------------------------------------------------

**hTGFbRII**  -E-----------------RKTELGKQY--WLITAFHAKGNLQEYLT-----------------------------------------------------

**Pi14992T0**  -WSDP--------------------EHL--CVISEYVKGGDLTVVLE-----------------------------------------------------

**Ps157753**  ----------------------------------------------------------------------------------------------------

**Pi04971T0**  PTNSMSESMA---AKSRYPSMGAPAWHL--GVVFEYMQHGSLAAMFE-----------------------------------------------------

**Ps136072**  PTNS-----TSATTKSRYPNMGAPSWHL--GVVFEYMQHGSLATMFE-----------------------------------------------------

**Pr93648**  PTNSMGDSLARSTTKSRYTNMGAPSWNL--GVVFEYMQHGSLATMFE-----------------------------------------------------

**Pu03658**  -------------------------WKF--AVVFEYMHNGSLTAMFQ-----------------------------------------------------

**AtCKI1**  -------------------G-----EYS--CMVIDLL-GPSLEDLFN-----------------------------------------------------

**hCKIalpha2**  -------------------K-----DYN--VLVMDLL-GPSLEDLFN-----------------------------------------------------

**mCKIalpha**  -------------------K-----DYN--VLVMDLL-GPSLEDLFN-----------------------------------------------------

**310 320 330 340 350 360 370 380 390 400**

**....|....|....|....|....|....|....|....|....|....|....|....|....|....|....|....|....|....|....|....|**

**AT1G06840_LRRVIII-1** ------------------------------------------------------VKLKEP---------------LDF----------------------

**AT4G29180_LRRI**  ------------------------------------------------------SENAED---------------LSW----------------------

**AT3G14840_LRRVIII-2** ----------------------------------------------------GPQETQIP---------------LNW----------------------

**AtNIK1_LRRII**  --------------------------------------------------------AKPV---------------LDW----------------------

**AtNAK**  ----------------------------------------------------RRGTFYQP---------------LSW----------------------

**AT2G24230_LRRVII**  --------------------------DLPFGVQTTDDWTTDTWEEETDNGTQNIGTEGPV---------------ATW----------------------

**AtBRI1_LRRXb**  ----------------------------------------------------DPKKAGVK---------------LNW----------------------

**AtCLV1_LRRXI**  ------------------------------------------------------GSKGGH---------------LQW----------------------

**AtFEI1_LRRXIIIa**  ------------------------------------------------------VERGEQ---------------LDW----------------------

**AtER_LRRXIIIb**  -----------------------------------------------------GPTKKKT---------------LDW----------------------

**AtRPK1_LRRXV**  ------------------------------------------------------ERSKAA---------------IEW----------------------

**AtTMK1_LRRIX**  ---------------------------------------------------EWSEEGLKP---------------LLW----------------------

**AT3G28450_LRRXa**  -------------------------------------------------------SNRGE---------------LDW----------------------

**AtIMK3_LRRIII**  -----------------------------------------------------ARGPDVH---------------INW----------------------

**AT2G45340_LRRIV**  ----------------------------------------------------VKDETGEV---------------LEW----------------------

**AtSCM_SUB_LRRV**  ----------------------------------------------------LDRKLHKK---------------LTW----------------------

**AtFLS2_LRRXII**  -------------------------------------------------------GSAAP---------I-----GSL----------------------

**dmPELLE**  --------------------------------------------------AHKAQNPLPA---------------LTW----------------------

**drIRAK1**  -------------------------------------------------------NEDSN---------A-----LSW----------------------

**hIRAK1**  -----------------------------------------------------CQTQACP---------P-----LSW----------------------

**xtPELLE**  ------------------------------------------------------PQGRFP---------K-----LPM----------------------

**AtCTR1_Raf**  ------------------------------------------------------KSGARE---------Q-----LDE----------------------

**Mb27170**  --------------------------------------------------------NESI---------D-----LPW----------------------

**Mb28586**  --------------------------------------------------------DGSY---------P-----LDW----------------------

**hRaf1**  -------------------------------------------------------VQETK---------------FQM----------------------

**Mb37485**  --------------------------------------------------------DVKE---------D-----LPV----------------------

**Esi0009_0077**  --------------------------------------------------------KAKE---------P-----LEE----------------------

**Esi0009_0083**  --------------------------------------------------------KAKE---------P-----LEE----------------------

**Esi0020_0071**  --------------------------------------------------------TSEQ---------P-----LPQ----------------------

**Esi0173_0029**  --------------------------------------------------------NSEQ---------P-----LPE----------------------

**Ch136568**  ---------------------------------------------------------GKK---------------VSWY---------------------

**Ch50123**  ---------------------------------------------------------AKK---------------VNWY---------------------

**Ch137597**  -----------------------------------------------------DAELQQR---------------LRWE---------------------

**Ch137605**  -----------------------------------------------------DEGRRER---------------LHWA---------------------

**Ch56654**  -----------------------------------------------------DEGRRER---------------LRWA---------------------

**Ch141610**  -----------------------------------------------------QADKREA---------------LRWR---------------------

**Ch143410**  -----------------------------------------------------DAHKRAA---------------LHWL---------------------

**Ch141452**  -----------------------------------------------------DEARRPR---------------LAWH---------------------

**Mb10450**  ---------------------------------------------------------KEH---------------LPW----------------------

**Mb25375**  ------------------------------------------------------GSQPRD---------------LST----------------------

**Mb28923**  ------------------------------------------------------GCQPRD---------------LNL----------------------

**Mb37923**  ------------------------------------------------------GRQPIL---------------LTD----------------------

**Mb34096**  ---------------------------------------------------------KES---------------LTR----------------------

**Mb36839**  ------------------------------------------------------NSLGPD---------------LDF----------------------

**Mb36426**  -------------------------------------------------------ETPEL---------------FDW----------------------

**hAXL**  -----------------------------------------------------LGDQPVY---------------LPT----------------------

**hHGFR**  ---------------------------------------------------------------------------PTV----------------------

**hRYK**  ----------------------------------------------------VEANNPQA---------------ISQ----------------------

**hDDR**  -------------------------------------------------PGDGQAAQGPT---------------ISY----------------------

**hIR**  --------------------------------------------------------RPPP----------------TL----------------------

**hLTK**  -----------------------------------------------------HLGQPSP---------------LVM----------------------

**hMuSK**  -------------------------------------------------S----SPGPPP---------------LSC----------------------

**hTRKalpha**  -------------------------------------------------E----DVAPGP---------------LGL----------------------

**hKLGlikePTK7**  --------------------------------------------------------KSQP---------------LST----------------------

**hFGFR2**  -------------------------------------------------SYDINRVPEEQ---------------MTF----------------------

**hRET**  --------------------------------LGSGGSRN---------SSSLDHPDERA---------------LTM----------------------

**hVGFR1**  --------------------------------FASSGFQEDKSLSDVEEEEDSDGFYKEP---------------ITM----------------------

**hPDGFRbeta**  ESVDYVPMLDMKGDVKYADIESSNYMAPYDNYVPSAPERT---------CRATLINESPV---------------LSY----------------------

**hTIE1**  -------------------------------------------------AFAREHGTAST---------------LSS----------------------

**hEGFR**  ---------------------------------------------------------------------------IGS----------------------

**hEPH**  ---------------------------------------------------------------------------LVP----------------------

**Pi00640T0**  ----------------------------------------------------ILKH-RES---------TVDNG-LTW--Q-------------------

**Pi19256T0**  ----------------------------------------------------ILKH-RES---------TVDNG-LTW--Q-------------------

**Pr72884**  ----------------------------------------------------ALKH-QGP---------NTNHE-LTW--Q-------------------

**Ps140986**  ----------------------------------------------------TLKH-QGS---------NVDHQGLTW--N-------------------

**Pi00643T0**  ----------------------------------------------------RFKR-RGY---------R-----LTW--E-------------------

**Pr72888**  ----------------------------------------------------RFKR-RSY---------R-----LTW--E-------------------

**Ps140988**  ----------------------------------------------------RFKR-RGY---------R-----LTW--E-------------------

**Pi00646T0**  ----------------------------------------------------SFQL-RNT---------P-----LTW--E-------------------

**Ps140991**  ----------------------------------------------------SFQL-RNT---------S-----LTW--E-------------------

**Pr72890**  ----------------------------------------------------SFKL-RNT---------P-----LTW--E-------------------

**Pi17831T0**  ----------------------------------------------------------------------------------------------------

**Ps132644**  ----------------------------------------------------TFKE-RGS---------P-----LSW--Q-------------------

**Pi17840T0**  ----------------------------------------------------GYKL-KGE---------Q-----LSW--E-------------------

**Pr76005**  ----------------------------------------------------GYKM-KGE---------R-----LSW--E-------------------

**Ps132640**  ----------------------------------------------------GYKL-KGE---------R-----LSW--E-------------------

**Pi17832T0**  ----------------------------------------------------SCKR-QGA---------R-----LSW--R-------------------

**Pr76010**  ----------------------------------------------------GFKR-QGN---------P-----LSW--R-------------------

**Ps132643**  ----------------------------------------------------SFKR-QGS---------P-----LSW--R-------------------

**Pi05112T0**  ----------------------------------------------------SS---RAS---------S-----LTW--A-------------------

**Pr82847**  ----------------------------------------------------SS---RAS---------S-----LTW--A-------------------

**Ps132270**  ----------------------------------------------------SS---RAS---------S-----LTW--A-------------------

**Pu14970**  ----------------------------------------------------SN---RAS---------S-----LTW--A-------------------

**S05981T0**  ----------------------------------------------------RS---STK---NDSNGTV-----LTW--A-------------------

**Pr81631**  ----------------------------------------------------KK---SPT---------R-----LPWQEG-------------------

**Pi02124T0**  ----------------------------------------------------KS---PPS---------V-----LTW--E-------------------

**Pr73421**  ----------------------------------------------------KS---PPS---------V-----LTW--E-------------------

**Ps144068**  ----------------------------------------------------KN---PPS---------V-----LTW--E-------------------

**S04928T0**  ----------------------------------------------------KK---KPS--------PT-----FDW--Q-------------------

**Pi07724T0**  ----------------------------------------------------GS----QT---------P-----VGW--T-------------------

**Ps158080**  ----------------------------------------------------SP----NT---------P-----VGW--T-------------------

**Pr77475A**  ----------------------------------------------------DP----DT---------P-----VGW--T-------------------

**Pi07725T0**  ----------------------------------------------------DT----ST---------P-----FGW--S-------------------

**Pi21299T0**  ----------------------------------------------------DT----ST---------P-----FGW--S-------------------

**Pr77476**  ----------------------------------------------------DA----ST---------A-----FGW--S-------------------

**Ps140003**  ----------------------------------------------------DA----ST---------P-----FGW--S-------------------

**Pi07731T0**  ----------------------------------------------------DS----NT---------P-----SGW--T-------------------

**Ps139996**  ----------------------------------------------------DP----DT---------P-----SGW--T-------------------

**Ps139997**  ----------------------------------------------------DP----DT---------P-----SGW--T-------------------

**Pr77470**  ----------------------------------------------------HV----ST---------P-----SGW--T-------------------

**Pr77475B**  ----------------------------------------------------SA----NA---------P-----LDW--T-------------------

**Pi09664T0**  ----------------------------------------------------DP----TS---------P-----RHW--S-------------------

**Pr75814**  ----------------------------------------------------DP----NS---------P-----RHW--S-------------------

**Ps135332**  ----------------------------------------------------DP----NS---------P-----RHW--S-------------------

**Ps135334**  ----------------------------------------------------DP----NS---------P-----RHW--S-------------------

**Pi23090T0**  ----------------------------------------------------AS----YL---------P-----HYW--T-------------------

**Pi23143T0**  ----------------------------------------------------AS----YL---------P-----HYW--T-------------------

**Pr81229**  ----------------------------------------------------AS----YL---------P-----RYW--T-------------------

**Pr75791**  ----------------------------------------------------AP----SL---------P-----RYW--T-------------------

**Pi09665T0**  ----------------------------------------------------KT---KSG---------S-----SLW--N-------------------

**Ps135333**  ----------------------------------------------------KT---KSS---------P-----RNW--N-------------------

**Ps127796**  ----------------------------------------------------RP----GT---------L-----REW--T-------------------

**Pi13397T0**  ----------------------------------------------------QQ---HER---QEMYPKE-----FDW------YQNSSVL---------

**Ps140146**  ----------------------------------------------------QQ---HER---QEMYPKE-----FDW------YQNSSVL---------

**Pr81472**  ----------------------------------------------------QQ---NGR---QELYPKE-----FDW------YQNSSVL---------

**Pi14970T0**  ----------------------------------------------------QK---HAE----KDGRRT-----FSW------VGENSQP---------

**Ps138087B**  ----------------------------------------------------QK---RSE----EGSRRT-----FSW------VGESSRP---------

**Pr75692**  ----------------------------------------------------QK---RRE----KNYRRT-----FSW------LGDDSQP---------

**Pi14971T0**  ----------------------------------------------------KQ---LKRETRDPSARGS-----YGW--FRSTKSERGGV---------

**Pi14972T0**  ----------------------------------------------------KQ---LQRELDAEYARDG-----YGW--FHSV-GEGENL---------

**Pr75691A**  ----------------------------------------------------KQ---QQREARDEFARDG-----YSW--FHSV-GNADNM---------

**Ps138088**  ----------------------------------------------------KQ---LQRETRDEFARDG-----YSW--FHSV-GEGDNM---------

**S06057T0**  ----------------------------------------------------AK---DDDDSQRSSAALK-----LVWPAQTSVKT--------------

**S02178T0**  ----------------------------------------------------RS---K-HE-------IV-----LPW--NV---ASTAALPTPSDEKAL

**S02311T0**  ----------------------------------------------------TD---HDAQR-------------IPW--HVQDEKDTVMLAPA------

**S02316T0**  ----------------------------------------------------VQ---KLND-------TP-----LGW--QL---QKDATVPLRPYSPCI

**S13911T0**  ----------------------------------------------------SR---P-----------H-----LAW--TA---ADAKRANVDSD----

**Pi05340T0**  ----------------------------------------------------RQQMLDGQ---------R-----RGW--SWSTDEDSGFK---------

**Ps133283**  ----------------------------------------------------RQRMLDSQ---------R-----RGW--NWTTEEDSGFA---------

**Ps133286**  ----------------------------------------------------RQRMLDSQ---------R-----RGW--NWTTEEDSGFA---------

**Pr80778**  ----------------------------------------------------RQQMLDSQ---------R-----RGW--SWTADEDSTFT---------

**S15848T0**  ----------------------------------------------------RD---ATAQ--------A-----LHW--T---ATSDTFP---------

**S15849T0**  ----------------------------------------------------RD---LDTQ--------S-----LHW--T---MPSSTFP---------

**S15852T0**  ----------------------------------------------------RD---LDTQ--------S-----LHW--T---MPSDTFP---------

**S01258T0**  ----------------------------------------------------DH---SMT---------Q-----FTW----------------------

**S09257T0**  ----------------------------------------------------NQ---SPE---------Q-----FSW----------------------

**S15543T0**  ----------------------------------------------------NQ---SPA---------Q-----FSW----------------------

**S09298T0**  ----------------------------------------------------SR---PPA---------T-----YSW----------------------

**S15453T0**  ----------------------------------------------------SR---PPT---------T-----YSW----------------------

**S08727T0**  ----------------------------------------------------KT---TPD---------Q-----FTW----------------------

**S09308T0**  ----------------------------------------------------RH---SPT---------T-----YPW----------------------

**S01416T0**  ----------------------------------------------------RY---SPA---------E-----YTW----------------------

**S06128T0**  ----------------------------------------------------SH---KPN---------E-----FSW----------------------

**S12836T0**  ----------------------------------------------------QT---TLD---------Q-----VSW----------------------

**S17043T0**  ----------------------------------------------------HH---SPM---------T-----VSW----------------------

**S08987T0**  ---------------------------------------------------------RRP---------H-----FYW----------------------

**S13082T0**  ----------------------------------------------------AT---DDT---------S-----FLW----------------------

**S05673T0**  ----------------------------------------------------ATTEDKEV---------L-----FPW----------------------

**S09620T0**  ----------------------------------------------------ATAEDKEV---------L-----FPW----------------------

**S12826T0**  ----------------------------------------------------RH---SQI---------S-----LPW----------------------

**S03267T0**  ---------------------------------------------------------LDP---------N-----MKW--P-------------------

**S13910T0**  ----------------------------------------------------EP---PM----------------HAW----------------------

**Pi05338T0**  ----------------------------------------------------EK---KHS---RKSSRST-----FSW------LEVSDVS---------

**Pr80780**  ----------------------------------------------------EK---KSS-------RKT-----FTW------MESTSES---------

**Ps133281**  ----------------------------------------------------EK---KPA---RKNSRPT-----FTW------LESSNET---------

**Pi22892T0**  ----------------------------------------------------KAET-TTE---------F-----KGWRSQS------------------

**Pr94275**  ----------------------------------------------------EVEN-SKE---------L-----CSWRSHS------------------

**Ps135323**  ----------------------------------------------------SVDGASHD---------L-----RSWRSQS------------------

**S01930T0**  ----------------------------------------------------SS---SVHDD-------S-----WSW--F-------------------

**Ps133943**  ------------------------------------------------------RTASGQ---------A-----GKWS---------------------

**S03896T0**  --------------------------------------------------LDQSRSSAER---------L-----FKWMPRSSNA---------------

**S09249T0**  ------------------------------------------------------NLNTQQ---------------ILW----------------------

**Pi03775T0**  -------------------------------------------------------NNAIE---------------LSV----------------------

**Pr75312**  -------------------------------------------------------NNAME---------------LSV----------------------

**Ps136506**  -------------------------------------------------------NNAVE---------------LSV----------------------

**AT3G14840_LRRXIV**  ---------------------------------------------------------YKSNSLVDNGLRS-----LDW----------------------

**Ps141628**  ----------------------THKRALQAKKAERPSG---DNSLDKRESDDDYDVDALQSEDEDSTMLADGGNRWSWRSSSSAY---------------

**Pu08017**  ----------------------KERELAREEMKLQQAGFGRENSIVDETGRPDH---------------------WTWQYDSASY---------------

**S14923T0**  --------------------------------------------------------TASL---------P-----LTW----------------------

**S14924T0**  -------------------------------------------------------NQDVD---------------IAW----------------------

**Mb37167**  --------------------------------------------------------LQGA---------N-----MPW----------------------

**AtAME2**  ------------------------------------------------------KNSYRS---------------FPI----------------------

**hCLK1**  ---------------------------------------------------------------------ENGFLPFRL----------------------

**AtCDC2a**  ------------------------------------------------------STPDFS---------------KDL----------------------

**hCDK3**  ------------------------------------------------------STPGSE---------------LPL----------------------

**AtMPK1**  --------------------------------------------------------SSQV---------------LSN----------------------

**hMAPK1**  ---------------------------------------------------------TQH---------------LSN----------------------

**AtGSK3b**  ----------------------------------------------------HYTSSNQR---------------MPI----------------------

**AtGSK3i**  ----------------------------------------------------HYTSSNQR---------------MPI----------------------

**AtCKA1**  -----------------------------------------------------------T---------------LTD----------------------

**hCK2a**  -----------------------------------------------------------T---------------LTD----------------------

**AtMEKK1**  ---------------------------------------------------------RYQ---------------LRD----------------------

**hMAPKKK1**  --------------------------------------------------------KYGA---------------FKE----------------------

**AtMKK3**  --------------------------------------------------------VTKK---------------IPE----------------------

**hMAPKK1**  --------------------------------------------------------KAGR---------------IPE----------------------

**AtCPK7**  --------------------------------------------------------ARGH---------------YTE----------------------

**hCaMK1**  --------------------------------------------------------EKGF---------------YTE----------------------

**AtNPH1**  ------------------------------------------------------RQPRKV---------------LKE----------------------

**AtPVPKlikePK5**  ------------------------------------------------------RQPGKH---------------FSE----------------------

**AtS6KlikePK1**  --------------------------------------------------------HQGL---------------FRE----------------------

**hGRK6**  ------------------------------------------------------HMGQAG---------------FPE----------------------

**AtSnRK2**  --------------------------------------------------------NAGR---------------FSE----------------------

**OUTGROUP1**  ---------------------------------------------------------KGP---------------LSP----------------------

**OUTGROUP2**  --------------------------------------------------------ENGA---------------LSP----------------------

**OUTGROUP3**  --------------------------------------------------------KNPR---------------IPI----------------------

**OUTGROUP4**  --------------------------------------------------------TEGP---------------MTP----------------------

**AtTousled**  --------------------------------------------------------ATSN---------------LPE----------------------

**hTousledLK1**  --------------------------------------------------------QHKL---------------MSE----------------------

**S08428T0**  --------------------------------------------------------RTSP---------------LEP----------------------

**S08432T0**  ---------------------------------------------------------------------------LRWTTPTSNGVV-------------

**Pi03773T0**  -------------------------------------------------------SAGSK---------------LNWV---------------------

**Pr75310**  -------------------------------------------------------SSGSE---------------LNWK---------------------

**Ps136501**  -------------------------------------------------------SAGTE---------------LNWS---------------------

**Pu07161**  -------------------------------------------------------QSGND---------------LTWG---------------------

**S09505T0**  -------------------------------------------------------HGHRS---------------LSWH---------------------

**AT1G14390_LRRVI-1**  -----------------------------------------------------DWRKKEV---------------LKW----------------------

**AT5G41180_LRRVI-2**  ------------------------------------------------------YADGSL---------------VSW----------------------

**hTGFbRI**  ---------------------------------------------------------RYT---------------VTV----------------------

**hTGFbRII**  ---------------------------------------------------------RHV---------------ISW----------------------

**Pi14992T0**  -------------------------------------VDGLDRPNRNNSSLSLLSASSADSDRKRSIFAANGMSKTSL----------------------

**Ps157753**  ---------------------------------------------------------------------------RAL----------------------

**Pi04971T0**  -------------SERHRREGKGFYPNSSVAAAIGSGNGNIFSWYPVFANSSASVNANPN---------------ADW----------------------

**Ps136072**  -------------SERHRREGKGFHPNSSVAASIGSGNGNIFSWYPVFANSSASVNANPN---------------ADW----------------------

**Pr93648**  -------------AERHRREGKGFYPNSSIAAAIGSGNGNIFSWYPVFANSSASVNANPN---------------ADW----------------------

**Pu03658**  -------------SERRRREGKKYMSNSSVNNLIKG--GNIFSWFPVVPAQENPETAD-----------------YDW----------------------

**AtCKI1**  -------------------------------------------------------YCKRI---------------FSL----------------------

**hCKIalpha2**  -------------------------------------------------------FCSRR---------------FTM----------------------

**mCKIalpha**  -------------------------------------------------------FCSRR---------------FTM----------------------

**VIa VIb**

**---------------- -------**

**410 420 430 440 450 460 470 480 490 500**

**....|....|....|....|....|....|....|....|....|....|....|....|....|....|....|....|....|....|....|....|**

**AT1G06840_LRRVIII-1** -------------------------AMRLRIALGSAKGILYLHTE--ANP-------P-IFHRDIKA----------------S----------------

**AT4G29180_LRRI**  -------------------------EKRLHIAIDSAQGLEYLHHG--CRP-------P-IVHRDVKT----------------A----------------

**AT3G14840_LRRVIII-2** -------------------------PMRQKICVGIARGLAYLHEE--SRL-------K-IVHRDIKA----------------T----------------

**AtNIK1_LRRII**  -------------------------SIRKRIAIGAARGLVYLHEQ--CDP-------K-IIHRDVKA----------------A----------------

**AtNAK**  -------------------------NTRVRMALGAARGLAFLH-N--AQP-------Q-VIYRDFKA----------------S----------------

**AT2G24230_LRRVII**  -------------------------RFRHKIALGTARALAFLHHG--CSP-------P-IIHRDVKA----------------S----------------

**AtBRI1_LRRXb**  -------------------------STRRKIAIGSARGLAFLHHN--CSP-------H-IIHRDMKS----------------S----------------

**AtCLV1_LRRXI**  -------------------------ETRHRVAVEAAKGLCYLHHD--CSP-------L-ILHRDVKS----------------N----------------

**AtFEI1_LRRXIIIa**  -------------------------DSRVNIIIGAAKGLSYLHHD--CSP-------R-IIHRDIKS----------------S----------------

**AtER_LRRXIIIb**  -------------------------DTRLKIAYGAAQGLAYLHHD--CSP-------R-IIHRDVKS----------------S----------------

**AtRPK1_LRRXV**  -------------------------KVLHKIALDVARALSYLHEQ--CSP-------K-VLHRDIKP----------------S----------------

**AtTMK1_LRRIX**  -------------------------KQRLTLALDVARGVEYLHGL--AHQ-------S-FIHRDLKP----------------S----------------

**AT3G28450_LRRXa**  -------------------------STRFRIGLGAARGLAWLHHG--CRP-------P-ILHQNICS----------------S----------------

**AtIMK3_LRRIII**  -------------------------PTRMSLIKGMARGLFYLHTH--AN----------IIHGNLTS----------------S----------------

**AT2G45340_LRRIV**  -------------------------ATRVSIINGIARGIVYLHGENGNKP-------A-IVHQNLSA----------------E----------------

**AtSCM_SUB_LRRV**  -------------------------NVRINIALGASKALQFLHEV--CQP-------P-VVHQNFKS----------------S----------------

**AtFLS2_LRRXII**  -------------------------LEKIDLCVHIASGIDYLHSG--YGF-------P-IVHCDLKP----------------A----------------

**dmPELLE**  -------------------------QQRFSISLGTARGIYFLHTA--RGT-------P-LIHGDIKP----------------A----------------

**drIRAK1**  -------------------------SQRVNVLLGTAKAIQYLH-S--CSP-------A-LIHGDIKS----------------S----------------

**hIRAK1**  -------------------------PQRLDILLGTARAIQFLH-Q--DSP-------S-LIHGDIKS----------------S----------------

**xtPELLE**  -------------------------EQRISILQGAACGLQYLH-N--YQP-------S-IIHGDVKS----------------S----------------

**AtCTR1_Raf**  -------------------------RRRLSMAYDVAKGMNYLH-N--RNP-------P-IVHRDLKS----------------P----------------

**Mb27170**  -------------------------SRRIAFARDAAAGMAFLH-A--LSP-------P-RIHRDLKS----------------P----------------

**Mb28586**  -------------------------ARRLAFALDAARGMEHLH-T--RTPV--------CLHRDLKSLWFRFLCLFSCFLFFTC----------------

**hRaf1**  -------------------------FQLIDIARQTAQGMDYLH-A--KN----------IIHRDMKS----------------N----------------

**Mb37485**  -------------------------DLCLSLATDAARGLRFLH-R--LRP-------P-QIHRDIKS----------------P----------------

**Esi0009_0077**  -------------------------KVLRKIVRDVCCGMAFLH-A--KA----------TVHGDLKS----------------A----------------

**Esi0009_0083**  -------------------------KVLRKIVRDVCCGMAFLH-A--KA----------TVHGDLKS----------------A----------------

**Esi0020_0071**  -------------------------EQSRRIIGDICAGMAFLH-E--KD----------TVHGDLKS----------------A----------------

**Esi0173_0029**  -------------------------EQSRQIIKDICAGMAFLH-S--KS----------TVHGDLKS----------------A----------------

**Ch136568**  -------------------------RKGKKIALDVARALVYLH-S--RR----------ILHLDIKS----------------A----------------

**Ch50123**  -------------------------RKGKKIALHIAKALVYLH-S--RR----------IVHLDIKS----------------A----------------

**Ch137597**  -------------------------AGGRQVALDVAEGLHHLHTT--QR----------VMHSDLKS----------------G----------------

**Ch137605**  -------------------------QGGCQVAADVAQALDHLHRQ--LG----------IMHGDLSS----------------G----------------

**Ch56654**  -------------------------QGGCQVAADVAQALDHLHRQ--LG----------IMHGDLSS----------------G----------------

**Ch141610**  -------------------------AGGRQAALEVASALVFLHTE--LN----------VLHSDLSS----------------S----------------

**Ch143410**  -------------------------ARGRQVAADVAGALDYLH-R--RN----------VMHGDLSS----------------S----------------

**Ch141452**  -------------------------EGGRQVAMDVAEALDYLHTQ--RQ----------IMHSDLKA----------------S----------------

**Mb10450**  -------------------------RTKLSIAADASRGLAFIH-S--LG----------HLHRDVKS----------------G----------------

**Mb25375**  -------------------------EQRVQILADVASGLAYLH-Q--LG----------HAHRDIKA----------------G----------------

**Mb28923**  -------------------------SQRLLILRDVATGLAYLH-E--LG----------HAHRDVKA----------------G----------------

**Mb37923**  -------------------------EQRCRILLDVAQGLAYLH-N--LG----------HTHRDIKS----------------G----------------

**Mb34096**  -------------------------AQKLSILLDVARGIEHLH-S--HG----------VAHRDLKS----------------G----------------

**Mb36839**  -------------------------AIRLRILVDVCRGVGFLH-S--LG----------LIHRDLKP----------------AISSTGLYAAHLHRFFL

**Mb36426**  -------------------------PRRCSLALDVARGMRYVHEE--MK----------SMHRDLKP----------------G----------------

**hAXL**  -------------------------QMLVKFMADIASGMEYLS-T--KR----------FIHRDLAA----------------R----------------

**hHGFR**  -------------------------KDLIGFGLQVAKGMKYLA-S--KK----------FVHRDLAA----------------R----------------

**hRYK**  -------------------------QDLVHMAIQIACGMSYLA-R--RE----------VIHKDLAA----------------R----------------

**hDDR**  -------------------------PMLLHVAAQIASGMRYLA-T--LN----------FVHRDLAT----------------R----------------

**hIR**  -------------------------QEMIQMAAEIADGMAYLN-A--KK----------FVHRDLAA----------------R----------------

**hLTK**  -------------------------RDLLQLAQDIAQGCHYLE-E--NH----------FIHRDIAA----------------R----------------

**hMuSK**  -------------------------AEQLCIARQVAAGMAYLS-E--RK----------FVHRDLAT----------------R----------------

**hTRKalpha**  -------------------------GQLLAVASQVAAGMVYLA-G--LH----------FVHRDLAT----------------R----------------

**hKLGlikePTK7**  -------------------------KQKVALCTQVALGMEHLS-N--NR----------FVHKDLAA----------------R----------------

**hFGFR2**  -------------------------KDLVSCTYQLARGMEYLA-S--QK----------CIHRDLAA----------------R----------------

**hRET**  -------------------------GDLISFAWQISQGMQYLA-E--MK----------LVHRDLAA----------------R----------------

**hVGFR1**  -------------------------EDLISYSFQVARGMEFLS-S--RK----------CIHRDLAA----------------R----------------

**hPDGFRbeta**  -------------------------MDLVGFSYQVANGMEFLA-S--KN----------CVHRDLAA----------------R----------------

**hTIE1**  -------------------------RQLLRFASDAANGMQYLS-E--KQ----------FIHRDLAA----------------R----------------

**hEGFR**  -------------------------QYLLNWCVQIAKGMNYLE-D--RR----------LVHRDLAA----------------R----------------

**hEPH**  -------------------------GQLVAMLQGIASGMNYLS-N--HN----------YVHRDLAA----------------R----------------

**Pi00640T0**  -------------------------DRKLTIALHIAEGLAYMH-S--LNP-------K-VIHRDLKS----------------K----------------

**Pi19256T0**  -------------------------DRKLTIALHIAEGLAYMH-S--LNP-------K-VIHRDLKS----------------K----------------

**Pr72884**  -------------------------GRKLTIALHIAEGLAYMH-S--LSP-------K-VIHRDLKS----------------K----------------

**Ps140986**  -------------------------GKKLTIALHIAEGLAYMH-S--LNP-------K-VIHRDLKS----------------K----------------

**Pi00643T0**  -------------------------THKTVIALHIAEALTHLH-G--LNP-------T-VIHRDLKA----------------K----------------

**Pr72888**  -------------------------AHKAAIALHIAEALTYLH-G--LSP-------T-VIHRDLKA----------------K----------------

**Ps140988**  -------------------------AHKTVIALHIAEALTYLH-G--LSP-------T-VIHRDLKA----------------K----------------

**Pi00646T0**  -------------------------AHKFRITQHIAEALTYLH-S--LDP-------K-LIHRDLKS----------------K----------------

**Ps140991**  -------------------------AHKLRITQHIAEALTYLH-S--LDP-------K-LIHRDLKS----------------K----------------

**Pr72890**  -------------------------AHKYRITQHIGEALTYLH-S--LDP-------K-LIHRDLKS----------------K----------------

**Pi17831T0**  ------------------------------------EALAYLH-C--QRP-------K-VIHRDLKS----------------K----------------

**Ps132644**  -------------------------THKASISLHIVEALAYLH-S--QHP-------K-VIHRDLKS----------------K----------------

**Pi17840T0**  -------------------------SHKATIALHIAEALAYLH-A--LKP-------K-IIHRDLKS----------------K----------------

**Pr76005**  -------------------------SHKATIALHIAEALSYLH-G--LKP-------M-IIHRDLKS----------------K----------------

**Ps132640**  -------------------------SHKATIALHIAEALSYLH-A--LKP-------K-IIHRDLKS----------------K----------------

**Pi17832T0**  -------------------------NHKTTISLHIAEAMQHLH-S--HNP-------K-VIHRDLKS----------------K----------------

**Pr76010**  -------------------------NHKTSISLHIAEAMEYLH-A--HSP-------K-VIHRDLKS----------------K----------------

**Ps132643**  -------------------------SHKAVIALHIAEAMHYLH-S--HSP-------K-VIHRDLKS----------------K----------------

**Pi05112T0**  -------------------------NLKCQIAIDIADALVYLH-T--LNP-------K-LIHRDLKS----------------R----------------

**Pr82847**  -------------------------NLKCQIAIDIADALVYLH-T--LNP-------K-LIHRDLKS----------------R----------------

**Ps132270**  -------------------------NLKCQIAIDIADALVYLH-T--LNP-------K-LIHRDLKS----------------R----------------

**Pu14970**  -------------------------NLKCQISIDIADALVYLH-T--LNP-------K-LIHRDLKS----------------R----------------

**S05981T0**  -------------------------NIKLHLAIDVADALVYLH-S--LNP-------K-FIHRDLKS----------------R----------------

**Pr81631**  -------------------------GQKLQYARDTIDAIVYLH-S--LSP-------V-IIHRDLKS----------------R----------------

**Pi02124T0**  -------------------------NGKATYCVQVCEAVYYLH-S--LQP-------A-LIHRDIKS----------------R----------------

**Pr73421**  -------------------------NGKATYCVQVCEAVYYLH-S--LQP-------A-LIHRDIKS----------------R----------------

**Ps144068**  -------------------------NGKAAYCVQVCEAVYYLH-S--LQP-------A-LIHRDIKS----------------R----------------

**S04928T0**  -------------------------SLKGTYALHVAKGLAYLH-G--KNP-------P-LIHRDIKA----------------R----------------

**Pi07724T0**  -------------------------RRKFDIAIGVVEALVYLH-S--FEP-------P-LVHRDLKS----------------K----------------

**Ps158080**  -------------------------NKKFNIAIGVIEALVYLH-S--FVP-------P-LVHRDLKS----------------K----------------

**Pr77475A**  -------------------------NRKFDIAIGIIEALVYLH-S--FVP-------Q-LVHRDLKS----------------K----------------

**Pi07725T0**  -------------------------FRKFDIAIGIIEALVYLH-S--FVP-------P-LVHRDLKS----------------K----------------

**Pi21299T0**  -------------------------FRKFDIAIGIIEALVYLH-S--FVP-------P-LVHRDLKS----------------K----------------

**Pr77476**  -------------------------FMKFDIAIGIIEALVYLH-S--FVP-------P-LVHRDLKS----------------K----------------

**Ps140003**  -------------------------LRKFDVAIGIIEALVYLH-S--FVP-------P-LVHRDLKS----------------K----------------

**Pi07731T0**  -------------------------TTKFNIAIDVIEALVYLH-S--FTP-------P-LVHRDLKS----------------N----------------

**Ps139996**  -------------------------TVKFNIAIDIIEALVYLH-S--FQP-------P-LVHRDLKS----------------K----------------

**Ps139997**  -------------------------TVKFNIAIDIIEALVYLH-S--FQP-------P-LVHRDLKS----------------K----------------

**Pr77470**  -------------------------PHKFGIAIDIIEALVYLH-S--LVP-------A-LVHRDLKS----------------K----------------

**Pr77475B**  -------------------------PEKFDIALGVVEALVYLH-S--FLP-------P-LVHRDLKS----------------R----------------

**Pi09664T0**  -------------------------QELLQLAADIIEALVYVH-S--FTP-------P-LVHRDLKS----------------R----------------

**Pr75814**  -------------------------QELLQLAVDIIEALVYVH-S--FTP-------P-LVHRDLKS----------------R----------------

**Ps135332**  -------------------------QELLQLAVDIIEALVYVH-S--FTP-------P-LVHRDLKS----------------R----------------

**Ps135334**  -------------------------QELLQLAVDIIEALVYVH-S--FTP-------P-LVHRDLKS----------------R----------------

**Pi23090T0**  -------------------------PTKFQLAIDTIEALVYVH-S--FNP-------P-LIHRDLKS----------------R----------------

**Pi23143T0**  -------------------------PTKFQLAIDTIEALVYVH-S--FNP-------P-LIHRDLKS----------------R----------------

**Pr81229**  -------------------------STKFQLAIDTVEALVYVH-S--FDP-------P-LVHRDLKS----------------R----------------

**Pr75791**  -------------------------RVKVQLAVDVIEALVYVH-S--FTP-------P-LAYRTLKS----------------R----------------

**Pi09665T0**  -------------------------AEKLQLSADVTEALVYVH-S--FTP-------P-IVHRDLKS----------------R----------------

**Ps135333**  -------------------------TEKLQLAADITEALVYVH-S--FTP-------P-IVHRDLKS----------------R----------------

**Ps127796**  -------------------------QQKLQIALEVAEALVYLH-S--FAP-------P-VLHRDLRA----------------S----------------

**Pi13397T0**  -------------------------PAKASIALDVLEAIVYLH-S--FPS-------P-IIHRDLKS----------------K----------------

**Ps140146**  -------------------------PAKAAIALDVLEAIVYLH-S--FPS-------P-IIHRDLKS----------------K----------------

**Pr81472**  -------------------------PAKAAIALDVLEAIVYLH-S--FPS-------P-IIHRDLKS----------------K----------------

**Pi14970T0**  -------------------------RSKIEIALDIAEGLVFMH-S--FDP-------T-IIHRDIKS----------------R----------------

**Ps138087B**  -------------------------RSKVDIALDTAEALVYMH-S--FDP-------T-IIHRDIKS----------------R----------------

**Pr75692**  -------------------------RNKVSIALDMAEALVYLH-S--FDP-------T-IIHRDIKS----------------R----------------

**Pi14971T0**  -------------------------KCKSLVALDIAEALVYLH-S--FES-------P-MIHRDLKP----------------N----------------

**Pi14972T0**  -------------------------KCKSLIALDIAEALVYLH-S--FES-------P-IIHRDMKP----------------K----------------

**Pr75691A**  -------------------------KCKSLMALDIAEALVYLH-S--FES-------P-IIHRDLKP----------------K----------------

**Ps138088**  -------------------------KCKSLIALDVAEALVYLH-S--FES-------P-IIHRDLKP----------------K----------------

**S06057T0**  -------------------------YSKVQVLSDVIAGLSYLH-E--LH----------VIHRDLKA----------------K----------------

**S02178T0**  PR---LTVSDVTELSTFTEYDPCCPVSKVSILADMADALAYLH-C--LRP-------T-IVHRDVKT----------------K----------------

**S02311T0**  -------------------------LSKLGVLVDILKALVYLH-A--QD----------VIHRDLKA----------------K----------------

**S02316T0**  LRSRSRPPA----------------ISKFSFLVDIVEGLLYLH-T--RQP-------P-LVHRDLKA----------------K----------------

**S13911T0**  -------------------------VTKASLLLDVVKGLRYLH-A--CDP-------T-VVHRDLKA----------------K----------------

**Pi05340T0**  -------------------------HSKLTMALDVADALVYLH-S--FAE-------P-IMHRDLKA----------------Q----------------

**Ps133283**  -------------------------HSKLTVALDIADALVYLH-S--FAE-------P-IMHRDLKA----------------Q----------------

**Ps133286**  -------------------------HSKLTVALDIADALVYLH-S--FAE-------P-IMHRDLKA----------------Q----------------

**Pr80778**  -------------------------HSKLMVALDVADALVYLH-S--FAE-------P-IMHRDLKA----------------Q----------------

**S15848T0**  -------------------------VSKLQMAVNVIDAITYLH-S--FEP-------K-ILHRDLKS----------------R----------------

**S15849T0**  -------------------------VSKLQMAVNVIDAITYLH-S--FEP-------K-ILHRDLKS----------------R----------------

**S15852T0**  -------------------------VSKLQMAVNVIDAITYLH-S--FEP-------K-ILHRDLKS----------------R----------------

**S01258T0**  -------------------------SEKAPVVRSIVFGLIFLH-T--FDP-------P-IVHRDLKS----------------R----------------

**S09257T0**  -------------------------DRKLNCILSIVRGLVYLH-T--YKP-------P-IIHRDLKS----------------R----------------

**S15543T0**  -------------------------DQKLKSILSIVRGLVYLH-T--YKP-------P-IIHRDLKS----------------R----------------

**S09298T0**  -------------------------QEKFTSIMSIVRGLVYLH-T--FEP-------P-IIHRDLKS----------------R----------------

**S15453T0**  -------------------------QEKFTSIMSIVRGLVYLH-T--FEP-------P-IIHRDLKS----------------R----------------

**S08727T0**  -------------------------PQKHMSISSVVLGLLYLH-T--FET-------P-IIHRDLKS----------------R----------------

**S09308T0**  -------------------------AAKLEAIVSIVRGLIYLH-T--LDP-------P-IIHRDLRS----------------R----------------

**S01416T0**  -------------------------PAKVATMYCVVEALMYLH-S--LP----------VIHRDLKS----------------R----------------

**S06128T0**  -------------------------PAKISCIHSIVYALVYLH-S--MQ----------IIHRDLKS----------------R----------------

**S12836T0**  -------------------------AEKLEILRPIGRGLAYLH-E--RK----------VIHRDLKS----------------R----------------

**S17043T0**  -------------------------EFKHKIMSHILEGLVYLH-S--MQ----------IIHRDLKS----------------R----------------

**S08987T0**  -------------------------DEKATCMLSIAEGLVYMY-T--MN----------IIHRDLKS----------------R----------------

**S13082T0**  -------------------------DEKIQLMLSVAEGLVYLH-S--MF----------VIHRDLKS----------------R----------------

**S05673T0**  -------------------------RDKIRSARDMTRGLVYLH-N--QN----------VIHRDLKS----------------R----------------

**S09620T0**  -------------------------HDKIRSARDMTRGLVYLH-N--QN----------VIHRDLKS----------------R----------------

**S12826T0**  -------------------------SFKLQSLVQIAEALAYLH-A--IP----------VVHRDLKC----------------K----------------

**S03267T0**  -------------------------STKVRYALHIAEALEYLH-S--LD----------IVHRDIKA----------------K----------------

**S13910T0**  -------------------LDDMSGATKLSLLHDVVDGLRYLH-S--CDP-------I-VFHMDLKA----------------E----------------

**Pi05338T0**  -------------------------PSKLSIALQASEALVYLQ-S--FAP-------P-IIHGHLKA----------------D----------------

**Pr80780**  -------------------------PSKHSLALQISEALVYLQ-S--FAP-------P-VIHGYLKA----------------D----------------

**Ps133281**  -------------------------PSKLSFALQMSEALVYLQ-S--FAP-------P-VIHGHLKA----------------D----------------

**Pi22892T0**  -------------------------AWKLQVAFDVAEALAYAH-A--FKP-------T-LVHRNLTS----------------H----------------

**Pr94275**  -------------------------AWKLQVAFDMAEALA------------------------------------------------------------

**Ps135323**  -------------------------AWKLQVAFDVAEALAYAH-A--FSP-------R-LIHRDLTS----------------H----------------

**S01930T0**  -------------------------ERKYALALDLARALAYLH-A--QSP-------S-VIHRDVRA----------------R----------------

**Ps133943**  -------------------------VQKVHMLLDVNRALLYLH-S--MHP-------R-LVHGNCNS----------------R----------------

**S03896T0**  -------------------------LPKLSLVSDIALAIVCLH-S--FAP-------A-IMHRNLHA----------------R----------------

**S09249T0**  -------------------------KWKTKTLEQIVSALAYLHNT--AN----------FVHGHVCA----------------A----------------

**Pi03775T0**  -------------------------PKQHAIARQISEAMAFLH-K--QN----------IVHGRLNA----------------F----------------

**Pr75312**  -------------------------PKQHTIARQISEAMAFLH-K--QN----------IVHGRLNA----------------F----------------

**Ps136506**  -------------------------PKQHAIARQISEAIAFLH-K--QN----------IVHGRLNA----------------F----------------

**AT3G14840_LRRXIV**  -------------------------ITRLKIALGVAEGLAYLHHD--CSP-------S-VVHRDIQA----------------S----------------

**Ps141628**  -------------------------KSKLSIAIEVAQAVQYLH-S--FSQ-------P-LFHGNLSS----------------R----------------

**Pu08017**  -------------------------KSKLSIAVDIARGLAYLH-S--FSP-------S-LFHGNLSS----------------R----------------

**S14923T0**  -------------------------PQQICLAWQVASAVAYLH-S--TDD-------NHPRLWTLTS----------------H----------------

**S14924T0**  -------------------------PDTLRMCFQVASGLAYVH-S--QAEF--------ARTECLTS----------------R----------------

**Mb37167**  -------------------------ERRLNFMSDAASGMQYLH-L--NH----------KLHMDLKS----------------G----------------

**AtAME2**  -------------------------DLVRELGRQLLESVAYMH-D--LR----------LIHTDLKP----------------E----------------

**hCLK1**  -------------------------DHIRKMAYQICKSVNFLH-S--NK----------LTHTDLKP----------------E----------------

**AtCDC2a**  -------------------------HMIKTYLYQILRGIAYCH-S--HR----------VLHRDLKP----------------Q----------------

**hCDK3**  -------------------------HLIKSYLFQLLQGVSFCH-S--HR----------VIHRDLKP----------------Q----------------

**AtMPK1**  -------------------------DHCQYFLFQLLRGLKYIH-S--AN----------ILHRDLKP----------------G----------------

**hMAPK1**  -------------------------DHICYFLYQILRGLKYIH-S--AN----------VLHRDLKP----------------S----------------

**AtGSK3b**  -------------------------FYVKLYTYQIFRGLAYIHTA--PG----------VCHRDIKP----------------Q----------------

**AtGSK3i**  -------------------------FYVKLYTYQIFRGLAYIHTV--PG----------VCHRDVKP----------------Q----------------

**AtCKA1**  -------------------------YDIRYYIYELLKALDFCH-S--QG----------LMHRDVKP----------------H----------------

**hCK2a**  -------------------------YDIRFYMYEILKALDYCH-S--MG----------IMHRDVKP----------------H----------------

**AtMEKK1**  -------------------------SVVSLYTRQILDGLKYLH-D--KG----------FIHRDIKC----------------A----------------

**hMAPKKK1**  -------------------------SVVINYTEQLLRGLSYLH-E--NQ----------IIHRDVKG----------------A----------------

**AtMKK3**  -------------------------PVLSSLFHKLLQGLSYLH-G--VRH---------LVHRDIKP----------------A----------------

**hMAPKK1**  -------------------------QILGKVSIAVIKGLTYLREK--HK----------IMHRDVKP----------------S----------------

**AtCPK7**  -------------------------RAAAAVMKTIVEVVQICH-K--QG----------VMHRDLKP----------------E----------------

**hCaMK1**  -------------------------RDASRLIFQVLDAVKYLH-D--LG----------IVHRDLKP----------------E----------------

**AtNPH1**  -------------------------DAVRFYAAQVVVALEYLH-C--QG----------IIYRDLKP----------------E----------------

**AtPVPKlikePK5**  -------------------------QAVKFYIAESLLALEYLH-M--LG----------IVYRDLKP----------------E----------------

**AtS6KlikePK1**  -------------------------DLARVYTAEIVSAVSHLH-E--KG----------IMHRDLKP----------------E----------------

**hGRK6**  -------------------------ARAVFYAAEICCGLEDLH-R--ER----------IVYRDLKP----------------E----------------

**AtSnRK2**  -------------------------DEARFFFQQLISGVSYCH-A--MQ----------ICHRDLKL----------------E----------------

**OUTGROUP1**  -------------------------ERALTTLRQLAEGLSAIH-A--RG----------IIHRDIKP----------------E----------------

**OUTGROUP2**  -------------------------EVAVHLMKQLAEGVLYAH-E--NN----------IIHRDLKT----------------Q----------------

**OUTGROUP3**  -------------------------ETIVHIAKQIAAGLSHAH-Q--NG----------IIHRDIKP----------------Q----------------

**OUTGROUP4**  -------------------------KRAIEVIADACQALNFSH-Q--NG----------IIHRDVKP----------------A----------------

**AtTousled**  -------------------------KEARIIIVQIVQGLVYLN-K--KSQK--------IIHYDLKP----------------G----------------

**hTousledLK1**  -------------------------KEARSIVMQIVNALRYLN-E--IKPP--------IIHYDLKP----------------G----------------

**S08428T0**  -------------------------LVTLRIVQDVAIAVAYLH-Q--LDP-------P-IVHNALTP----------------N----------------

**S08432T0**  -------------------------PLKAQLALDVATAVAHLH-S--LAT-------P-ILHNAISI----------------D----------------

**Pi03773T0**  -------------------------NFKMKAAAEIARGLMYLH-S--QHM---------VTYDGLNG----------------R----------------

**Pr75310**  -------------------------NFKMKAASEIARGLMYLH-S--QHM---------VTYDGLNG----------------R----------------

**Ps136501**  -------------------------NFKMKAAAEIARGLMYLH-S--QHM---------ATYDGLNG----------------R----------------

**Pu07161**  -------------------------NFKLRAAMDIAGGLMYLH-S--KHK---------LSYDGLNG----------------R----------------

**S09505T0**  -------------------------SGKRQMALEVALALLQVH-E--HD----------FVYNGLTG----------------K----------------

**AT1G14390_LRRVI-1**  -------------------------PQRMSIAIGVARGIQFLHTG--VAPG--------IFGNNLEI----------------E----------------

**AT5G41180_LRRVI-2**  -------------------------AKRMKIVIGIARGLKYLHTE--LHP-------P-FTVSELSS----------------T----------------

**hTGFbRI**  -------------------------EGMIKLALSTASGLAHLHME--IVGTQGKP--A-IAHRDLKS----------------K----------------

**hTGFbRII**  -------------------------EDLRKLGSSLARGIAHLHSD--HTPC-GRPKMP-IVHRDLKS----------------S----------------

**Pi14992T0**  -------------------------TTRFQMALDICKALVYMH-N--KG----------VSHSDLRS----------------R----------------

**Ps157753**  -------------------------VTRFQMALDICKALAYMH-N--KG----------ISHSDLRS----------------R----------------

**Pi04971T0**  -------------------------RCKLSIALDVAMGLVYLH-A--NN----------YAHGRVCA----------------R----------------

**Ps136072**  -------------------------RCKLSIALDVAMGLVYLH-A--NN----------CAHGRVCA----------------R----------------

**Pr93648**  -------------------------RCKLSIALDVAMGLVYLH-A--NN----------YAHGRVCA----------------R----------------

**Pu03658**  -------------------------RCKLSIALDITMALVYLH-S--SQ----------LVHGSLNS----------------S----------------

**AtCKI1**  -------------------------KSVLMLADQLICRVEYMH-S--RG----------FLHRDIKP----------------D----------------

**hCKIalpha2**  -------------------------KTVLMLADQMISRIEYVH-T--KN----------FIHRDIKP----------------D----------------

**mCKIalpha**  -------------------------KTVLMLADQMISRIEYVH-T--KN----------FIHRDIKP----------------D----------------

**VII**

**--------**

**510 520 530 540 550 560 570 580 590 600**

**....|....|....|....|....|....|....|....|....|....|....|....|....|....|....|....|....|....|....|....|**

**AT1G06840_LRRVIII-1** ------NIL------L---D---S---R--FT--AKVADFGLS--------------------------------------RLAPVPDMEGISPQHV---

**AT4G29180_LRRI**  ------NIL------L---N---D---N--LE--AKIADFGLS--------------------------------------KVFPEDDL-----SHV---

**AT3G14840_LRRVIII-2** ------NVL------L---D---K---E--LN--PKISDFGLA--------------------------------------KLDEEEN------THI---

**AtNIK1_LRRII**  ------NIL------L---D---D---Y--CE--AVVGDFGLA--------------------------------------KLLDHQD------SHV---

**AtNAK**  ------NIL------L---D---S---N--YN--AKLSDFGLA--------------------------------------RDGPMGDN-----SHV---

**AT2G24230_LRRVII**  ------SVY------L---D---Q---N--WE--PRLSDFGLA--------------------------------------KVFGNGLD-----------

**AtBRI1_LRRXb**  ------NVL------L---D---E---N--LE--ARVSDFGMA--------------------------------------RLMSAMDT-----HLS---

**AtCLV1_LRRXI**  ------NIL------L---D---S---D--FE--AHVADFGLA--------------------------------------KFLVDGAA-----SEC---

**AtFEI1_LRRXIIIa**  ------NIL------L---D---G---N--LE--ARVSDFGLA--------------------------------------KLLEDEE------SHI---

**AtER_LRRXIIIb**  ------NIL------L---D---K---D--LE--ARLTDFGIA--------------------------------------KSLCVSK------SHT---

**AtRPK1_LRRXV**  ------NIL------L---D---N---N--YN--AYLSDFGLS--------------------------------------KLLGTSQ------SHV---

**AtTMK1_LRRIX**  ------NIL------L---G---D---D--MR--AKVADFGLV--------------------------------------RLAPEGK------GSI---

**AT3G28450_LRRXa**  ------VIL------I---D---E---D--FD--ARIIDSGLA--------------------------------------RLMVPSDNNE---SSF---

**AtIMK3_LRRIII**  ------NVL------L---D---E---N--IT--AKISDYGLS--------------------------------------RLMTAAAG-----------

**AT2G45340_LRRIV**  ------KIL------I---D---H---W--YN--PSLADSGLH--------------------------------------KLFTDDIV-----------

**AtSCM_SUB_LRRV**  ------KVL------L---D---G---K--LS--VRVADSGLA--------------------------------------YMLPPRPT-----------

**AtFLS2_LRRXII**  ------NIL------L---D---S---D--RV--AHVSDFGTA--------------------------------------RILGFRED-----GST---

**dmPELLE**  ------NIL------L---D---Q---C--LQ--PKIGDFGLV--------------------------------------REGPKSLD-----------

**drIRAK1**  ------NIL------L---G---D---H--LE--PKLGDFGLA--------------------------------------RLCRNPNK-----TPG---

**hIRAK1**  ------NVL------L---D---E---R--LT--PKLGDFGLA--------------------------------------RFSRFAGS-----SPS---

**xtPELLE**  ------NIL------L---D---Q---A--FM--PKLGDFGLA--------------------------------------RFSRYTSN-----AGN---

**AtCTR1_Raf**  ------NLL------V---D---K---K--YT--VKVCDFGLS--------------------------------------RLKASTF------------

**Mb27170**  ------NLL------I---S---Q---G--WT--LKVADFGTA--------------------------------------KLASLVSN-----QEG---

**Mb28586**  ------NLL------V---S---S---S--LT--VKVADFGTS--------------------------------------TLFDLTAA-----SGF---

**hRaf1**  ------NIF------L---H---E---G--LT--VKIGDFGLA--------------------------------------TVKSRWSG-----SQQ---

**Mb37485**  ------NLL------V---T---S---N--YV--CKVADLGTA--------------------------------------RLIEEMGI-----ERT---

**Esi0009_0077**  ------NVL------F---D---A---T--GR--AKIADFGTS--------------------------------------LWTQHTTR-----L-----

**Esi0009_0083**  ------NVL------F---D---A---T--GR--AKIADFGTS--------------------------------------LWTQHSTR-----L-----

**Esi0020_0071**  ------NVL------L---D---G---S--GR--AKIGDFGTS--------------------------------------RWSQHTHS-----TGL---

**Esi0173_0029**  ------NIL------L---D---A---R--GR--AKIGDFGTS--------------------------------------RWTQNTER-----STG---

**Ch136568**  ------NVL------L---T---R---D--GT--AKVGDVGMA--------------------------------------KIMAGDY------------

**Ch50123**  ------NVL------L---Q---R---D--GT--AKLGDVGLA--------------------------------------KIMAGGY------------

**Ch137597**  ------NVL------L---S---E---D--LR--AFLGDMSVA--------------------------------------QVVGSKA------------

**Ch137605**  ------NVL------L---D---G---G--LR--GHLGDLGLA--------------------------------------FSVASAAP-----------

**Ch56654**  ------NVL------L---D---G---G--LR--GHLGDLGLA--------------------------------------FSVASAAP-----------

**Ch141610**  ------NVL------L---D---A---G--LT--ASISDLGVA--------------------------------------RFVGSSA------------

**Ch143410**  ------NVL------L---D---G---S--LA--GHIGDLGMA--------------------------------------RFVAGSV------------

**Ch141452**  ------NVL------L---S---S---D--LR--ASLADLGVS--------------------------------------QVLLSSA------------

**Mb10450**  ------NCL------I---T---S---D--YR--CKINDFGTL--------------------------------------KRPSEDREREHLRGVE---

**Mb25375**  ------NVL------V---T---E---Q--YR--AKVTDFGSV--------------------------------------KLRERLVGVNASLTKP---

**Mb28923**  ------NVL------I---T---K---Q--FR--AKVTDFGSV--------------------------------------KLRQRALLSRTQLQRG---

**Mb37923**  ------NVL------I---T---A---D--FR--AKVSDFGSI--------------------------------------KPRAHASTHRAPGATG---

**Mb34096**  ------NVL------I---S---S---D--FR--AKVSDFGSV--------------------------------------KRFYRKAEGNDDMSDV---

**Mb36839**  VMDDVGNIL------I---D---RIRGD--LI--AKVTDFGTL--------------------------------------RTLMDTSM-----PMA---

**Mb36426**  ------NVL------V---T---R---D--WR--GKVNDFGSM--------------------------------------KYCFQRYQQKFASTSG---

**hAXL**  ------NCM------L---N---E---N--MS--VCVADFGLS--------------------------------------KKIYNGDY-----YRQ---

**hHGFR**  ------NCM------L---D---E---K--FT--VKVADFGLA--------------------------------------RDMYDKEY-----YSV---

**hRYK**  ------NCV------I---D---D---T--LQ--VKITDNALS--------------------------------------RDLFPMDY-----HCL---

**hDDR**  ------NCL------V---G---E---N--FT--IKIADFGMS--------------------------------------RNLYAGDY-----YRV---

**hIR**  ------NCM------V---A---H---D--FT--VKIGDFGMT--------------------------------------RDIYETDY-----YRK---

**hLTK**  ------NCL------L---SCAGP---S--RV--AKIGDFGMA--------------------------------------RDIYRASY-----YRR---

**hMuSK**  ------NCL------V---G---E---N--MV--VKIADFGLS--------------------------------------RNIYSADY-----YKA---

**hTRKalpha**  ------NCL------V---G---Q---G--LV--VKIGDFGMS--------------------------------------RDIYSTDY-----YRV---

**hKLGlikePTK7**  ------NCL------V---S---A---Q--RQ--VKVSALGLS--------------------------------------KDVYNSEY-----YHF---

**hFGFR2**  ------NVL------V---T---E---N--NV--MKIADFGLA--------------------------------------RDINNIDY-----YKK---

**hRET**  ------NIL------V---A---E---G--RK--MKISDFGLS--------------------------------------RDVYEEDS-----YVK---

**hVGFR1**  ------NIL------L---S---E---N--NV--VKICDFGLA--------------------------------------RDIYKNPD-----YVR---

**hPDGFRbeta**  ------NVL------I---C---E---G--KL--VKICDFGLA--------------------------------------RDIMRDSN-----YIS---

**hTIE1**  ------NVL------V---G---E---N--LA--SKIADFGLS--------------------------------------RG---EEV-----YVK---

**hEGFR**  ------NVL------V---K---T---P--QH--VKITDFGLA--------------------------------------KLLGAEEK-----EYH---

**hEPH**  ------NIL------V---N---Q---N--LC--CKVSDFGLT--------------------------------------RLLDDFDG-----TYE---

**Pi00640T0**  ------NVL------L---N---N---A--YE--AKLSDFGVS--------------------------------------RER--QV------ANVADV

**Pi19256T0**  ------NVL------L---N---N---A--YE--AKLSDFGVS--------------------------------------RER--QV------ANVADV

**Pr72884**  ------NVL------L---N---N---N--YD--AKLSDFGVS--------------------------------------REH--RV------AEG-DG

**Ps140986**  ------NVL------L---N---N---D--YE--AKLSDFGVS--------------------------------------RKR--LV------ADVNGG

**Pi00643T0**  ------NVL------L---N---A---D--ME--AKLSDFGIA--------------------------------------RERTFYDG-----SEH---

**Pr72888**  ------NVL------L---N---A---D--ME--AKLSDFGIA--------------------------------------RERSLYDG-----SEH---

**Ps140988**  ------NVL------L---N---A---D--ME--AKLSDFGIA--------------------------------------RERSFYDG-----SEH---

**Pi00646T0**  ------NVL------L---N---T---E--ME--AKLSDFGVS--------------------------------------RER--HA------MET---

**Ps140991**  ------NVL------L---N---T---E--MD--AKLSDFGVS--------------------------------------RER--HD------MET---

**Pr72890**  ------NVL------L---N---T---E--ME--AKLSDFGVS--------------------------------------RER--HA------MET---

**Pi17831T0**  ------NVL------L---N---L---Y--LE--AKLSDFGIS--------------------------------------RVR--YA------IET---

**Ps132644**  ------NVL------L---N---L---Y--YE--AKLSDFGIS--------------------------------------RMR--YD------MET---

**Pi17840T0**  ------NVL------L---N---M---Y--LE--AKLSDFGIS--------------------------------------RMH--YL------VET---

**Pr76005**  ------NVL------L---N---M---Y--LE--AKLSDFGIS--------------------------------------RMR--YL------VET---

**Ps132640**  ------NVL------L---N---M---Y--LE--AKLSDFGIA--------------------------------------RMR--YL------VET---

**Pi17832T0**  ------NVL------L---N---M---H--LE--AKLTDFGVS--------------------------------------RTQ--YT------VQT---

**Pr76010**  ------NVL------L---N---M---H--LE--AKLTDFGVS--------------------------------------RTQ--YT------VQT---

**Ps132643**  ------NVL------L---N---R---H--LE--AKLTDFGVS--------------------------------------RAQ--YS------VQT---

**Pi05112T0**  ------NVL------I---D---A---Q--TG--AKLSDFGIS--------------------------------------RNR--SF------DET---

**Pr82847**  ------NVL------I---D---A---Q--SG--AKLSDFGIS--------------------------------------RNR--SF------DET---

**Ps132270**  ------NVL------I---D---A---Q--TG--AKLSDFGIS--------------------------------------RNR--SF------DET---

**Pu14970**  ------NVL------I---D---A---Q--TG--AKLSDFGIS--------------------------------------RDR--SV------EET---

**S05981T0**  ------NIL------I---D---A---E--NG--AKLSDFGIS--------------------------------------RNR--NL------EET---

**Pr81631**  ------NIL------L---D---S---K--KG--AKLGDFGVS--------------------------------------ATK--RP------TD----

**Pi02124T0**  ------NIL------V---D---S---Q--KG--AKLSDFGES--------------------------------------RER--TV------ART---

**Pr73421**  ------NIL------V---D---S---Q--KG--AKLSDFGES--------------------------------------RER--TV------ART---

**Ps144068**  ------NIL------V---D---S---Q--KG--AKLSDFGES--------------------------------------RER--TV------ART---

**S04928T0**  ------NIL------V---D---S---Q--KG--AKICDFGES--------------------------------------RLR--SY------QET---

**Pi07724T0**  ------NVL------L---S---S---D--FK--AKLSDFGAS--------------------------------------RFR--SV------ENT---

**Ps158080**  ------NVL------L---S---S---D--FK--AKLSDFGAS--------------------------------------RFR--SV------ENT---

**Pr77475A**  ------NVL------L---S---S---D--FQ--AKLSDFGAS--------------------------------------RFR--SV------ENT---

**Pi07725T0**  ------NVL------L---S---A---D--FQ--AKLSDFGTS--------------------------------------RFR--SV------DNT---

**Pi21299T0**  ------NVL------L---S---A---D--FQ--AKLSDFGTS--------------------------------------RFR--SV------DNT---

**Pr77476**  ------NVM------L---S---A---D--FR--AKLSDFGTS--------------------------------------RFR--SV------ENT---

**Ps140003**  ------NVL------L---S---S---D--FK--AKLSDFGTS--------------------------------------RFR--SV------ENT---

**Pi07731T0**  ------NVL------L---S---S---D--ME--AKLSDFGTS--------------------------------------RFR--SS------DKT---

**Ps139996**  ------NVL------L---S---S---D--ME--AKLSDFGTS--------------------------------------RFR--SS------DKT---

**Ps139997**  ------NVL------L---S---S---D--ME--AKLSDFGTS--------------------------------------RFR--SS------DKT---

**Pr77470**  ------NVL------L---S---S---N--FK--AKLSDFGTS--------------------------------------RFR--SS------DKT---

**Pr77475B**  ------NVL------L---S---S---T--LQ--AKLSDFGTS--------------------------------------RFR--SE------ENT---

**Pi09664T0**  ------NVL------L---T---G---E--LK--AKVTDFGAS--------------------------------------RYK--SV------DET---

**Pr75814**  ------NVL------L---S---G---E--MQ--AKVTDFGAS--------------------------------------RYK--SM------DET---

**Ps135332**  ------NVL------L---S---G---E--MQ--AKVTDFGAS--------------------------------------RYK--SM------DET---

**Ps135334**  ------NVL------L---S---G---E--MQ--AKVTDFGAS--------------------------------------RYK--SM------DET---

**Pi23090T0**  ------NVL------I---S---A---E--MH--AKLTDFGTT--------------------------------------RYR--SI------DGT---

**Pi23143T0**  ------NVL------I---S---A---E--MH--AKLTDFGTT--------------------------------------RYR--SI------DGT---

**Pr81229**  ------NVL------I---S---A---D--IH--AKLTDFGTT--------------------------------------RYR--SK------DGT---

**Pr75791**  ------NVL------I---S---A---D--MR--AKLTDVGVS--------------------------------------GFR--SR------NGT---

**Pi09665T0**  ------NIL------L---S---Q---D--TR--GHLSDFGVA--------------------------------------RVR--SA------NDT---

**Ps135333**  ------NVL------L---S---E---D--MR--GHLSDFGVA--------------------------------------RVR--SA------NNT---

**Ps127796**  ------NVL------L---T---S---E--MK--AKVSKVGVS--------------------------------------HLR--ALA-----SGD---

**Pi13397T0**  ------NVL------L---S---A---S--YE--AKLSDFGVS--------------------------------------REW--QV------DTT---

**Ps140146**  ------NVL------L---S---A---S--YE--AKLSDFGVS--------------------------------------REW--QV------DTT---

**Pr81472**  ------NVL------L---S---S---S--YE--AKLSDFGVS--------------------------------------REW--QV------DTT---

**Pi14970T0**  ------NVL------L---N---H---E--WE--AKLSDFGIS--------------------------------------RET--SN------QTT---

**Ps138087B**  ------NVL------L---N---H---A--WE--AKLSDFGIS--------------------------------------RET--SD------EST---

**Pr75692**  ------NVL------L---N---H---E--WE--AKLSDFGIS--------------------------------------RER--SD------EST---

**Pi14971T0**  ------NVL------M---S---E---K--WE--AKLTDFGVS--------------------------------------REL--TE------DRT---

**Pi14972T0**  ------NVL------L---S---D---T--WE--AKLTDFGIS--------------------------------------REL--DE------DQT---

**Pr75691A**  ------NVL------L---S---D---T--WE--AKLTDFGIS--------------------------------------REL--DE------DQT---

**Ps138088**  ------NVL------L---S---E---K--WE--AKLTDFGIS--------------------------------------REL--DE------DQT---

**S06057T0**  ------NVL------L---G---A---Q--FE--AKLSDFGTS--------------------------------------RQV--VM------DAT---

**S02178T0**  ------NVL------L---D---A---N--WV--AKVADFGAS--------------------------------------RSYG-DS------DLV---

**S02311T0**  ------NVM------L---N---E---T--FV--AKVTDFGTS--------------------------------------RET----S-----EET---

**S02316T0**  ------NVL------L---S---C---D--FV--AKLTDFGVA--------------------------------------RE---TC------DYT---

**S13911T0**  ------NVL------I---G---T---N--FV--AKLGDFGSS--------------------------------------RLYD-ED------DNT---

**Pi05340T0**  ------NIL------L---S---N---K--WV--ATISDFGVS--------------------------------------KRHKQRNE-----ERG---

**Ps133283**  ------NVL------L---S---N---K--WV--AKISDFGVS--------------------------------------KRRKQRDD-----KRG---

**Ps133286**  ------NVL------L---S---N---K--WV--AKISDFGVS--------------------------------------KRRKQRDD-----KRG---

**Pr80778**  ------NIL------L---S---N---K--WV--AKISDFGVS--------------------------------------KGRKQRDG-----QKG---

**S15848T0**  ------NIL------L---D---G---Y--YA--AKLTDFGIS--------------------------------------RS---MI------DET---

**S15849T0**  ------NIL------L---D---A---T--YV--AKLT-------------------------------------------RL---VV------DET---

**S15852T0**  ------NIL------L---D---A---T--YV--AKLTDFGIS--------------------------------------RL---VV------DET---

**S01258T0**  ------NIL------L---D---S---V--KG--TKLTDFGIS--------------------------------------REV----------DDL---

**S09257T0**  ------NVL------L---D---S---V--KG--TKLTDFGES--------------------------------------RVAEE--------DDL---

**S15543T0**  ------NVL------L---D---S---V--KG--TKLTDFGES--------------------------------------RVAEE--------DDL---

**S09298T0**  ------NVL------L---D---S---K--KG--TKLTDFGAS--------------------------------------RQEA---------DAG---

**S15453T0**  ------NVL------L---D---S---A--KG--TKLTDFGAS--------------------------------------RQEA---------DAG---

**S08727T0**  ------NVL------L---D---S---K--KG--TKLTDFGES--------------------------------------REVD---------ENT---

**S09308T0**  ------NVL------L---D---S---E--KG--TKINDFGSS--------------------------------------RETS---------EQS---

**S01416T0**  ------NVL------L---D---D---A--KG--AKLTDFGVS--------------------------------------KEDT---------QAT---

**S06128T0**  ------N-----------------------------------------------------------------------------------------T---

**S12836T0**  ------NVL------L---D---A---K--KG--AKLSDFGVS--------------------------------------KAES---------ENT---

**S17043T0**  ------NVL------L---D---S---SDGET--VKLTDFGVS--------------------------------------KEDL---------QET---

**S08987T0**  ------NIL------L---D---A---I--KG--TKLTDFGVS--------------------------------------REMT---------TET---

**S13082T0**  ------NVL------L---D---T---V--KG--AKLTDFGVS--------------------------------------KEMT---------TET---

**S05673T0**  ------NVM------L---T---A---A--LD--AKLGDFGIA--------------------------------------RDAT---------DES---

**S09620T0**  ------NVM------L---T---A---A--LD--AKLGDFGIA--------------------------------------RDAT---------DES---

**S12826T0**  ------NVL------L---D---E---K--KG--TKLGGFSIS--------------------------------------RET----------SAT---

**S03267T0**  ------NVL------V---DP--S---E--NG--AKVCDFGVA--------------------------------------LHLHPTV------EDTV--

**S13910T0**  ------HVF------V---S---E---S--FE--AKLGAFGST--------------------------------------RATHHAE------DVT---

**Pi05338T0**  ------SLL------L---G---S---A--WE--VKLNRLGYR--------------------------------------LDA----------------

**Pr80780**  ------SIL------L---G---P---A--WE--IKLNRLGYK--------------------------------------LDA----------------

**Ps133281**  ------SVL------L---G---S---S--WE--IKLNRLGYM--------------------------------------LNT----------------

**Pi22892T0**  ------SVL------L---S---S---SPDCR--ALLDNFIIA--------------------------------------IKG--YPS-----TST---

**Pr94275**  -------VL------L---S---S---PPELR--ARLDDFVAE--------------------------------------QQD--SAG-----MST---

**Ps135323**  ------SVL------L---S---S---PPELR--ARLDEFVIE--------------------------------------QQG--FPS-----MPT---

**S01930T0**  ------SVL------L---D---S---R--YR--CQLSHFGSA--------------------------------------RVR--SI------LDT---

**Ps133943**  ------NVL------L---D---H---A--LR--AKLSDYGVD--------------------------------------GRADGLTE-----QEL---

**S03896T0**  ------HVL------L---T---N---D--YV--AKLSGFRPQ--------------------------------------DAEHEPPR-----------

**S09249T0**  ------NVL------L---D---G---V--KG--AKLMMLDAQ---------------------------------------------------------

**Pi03775T0**  ------NVL------L---D---K---D--YS--AKLSLFSIF--------------------------------------HYVKL--------------

**Pr75312**  ------NIL------L---D---K---E--YS--AKLSLFSIF--------------------------------------HYVKL--------------

**Ps136506**  ------NIL------L---D---K---D--YS--AKLSLFSIF--------------------------------------HYVKL--------------

**AT3G14840_LRRXIV**  ------SIL------L---D---D---K--FE--VRLGSFSKA--------------------------------------CHQENNGR-----PR----

**Ps141628**  ------KVF------L---D---S---D--WN--VKLGDLTCC--------------------------------------SALRRWSS-----SHN---

**Pu08017**  ------KVL------L---D---E---S--WN--VKLNDLSCC--------------------------------------SALWRWSE-----RQQ---

**S14923T0**  ------HVL------V---N---T---Q--LV--CKLNVFDFM--------------------------------------ANYEHVDA-----VVG---

**S14924T0**  ------SVL------V---N---T---Q--LT--CKLNIFEYM--------------------------------------RHFQQHET-----------

**Mb37167**  ------NCL------V---S---S---S--WT--LKLTDFATT--------------------------------------TAVAQSAE-----EAS---

**AtAME2**  ------NIL------L---V---S---S--EY--IKIPDYKFL--------------------------------------SRPTKDGS-----YFK---

**hCLK1**  ------NILFVQSDYTEAYNPKIKRDERTLINPDIKVVDFGSA---------------------------------------------------------

**AtCDC2a**  ------NLL------I---D---R--RT--NS--LKLADFGLA--------------------------------------RAFGIPVR-----------

**hCDK3**  ------NLL------I---N---E---L--GA--IKLADFGLA--------------------------------------RAFGVPLR-----------

**AtMPK1**  ------NLL------V---N---A---N--CD--LKICDFGLA--------------------------------------RASNTKGQ-----------

**hMAPK1**  ------NLL------L---N---T---T--CD--LKICDFGLA--------------------------------------RVADPDHD-----------

**AtGSK3b**  ------NLL------V---D---P--HT--HQ--CKLCDFGSA--------------------------------------KVLVKGEP-----------

**AtGSK3i**  ------NLL------V---D---P--LT--HQ--VKLCDFGSA--------------------------------------KVLVKGEP-----------

**AtCKA1**  ------NVM------I---D---H---EL-RK--LRLIDWGLA--------------------------------------EFYHPGKE-----------

**hCK2a**  ------NVM------I---D---H---EH-RK--LRLIDWGLA--------------------------------------EFYHPGQE-----------

**AtMEKK1**  ------NIL------V---D---A---N--GA--VKLADFGLA--------------------------------------KVSKFND------------

**hMAPKKK1**  ------NLL------I---D---S---TG-QR--LRIADFGAA--------------------------------------ARLASKGT-----GAG---

**AtMKK3**  ------NLL------I---N---L---K--GE--PKITDFGIS--------------------------------------AGLENSMA-----M-----

**hMAPKK1**  ------NIL------V---N---S---R--GE--IKLCDFGVS--------------------------------------GQLIDSM------------

**AtCPK7**  ------NFL------FANKK---E---T--SA--LKAIDFGLS--------------------------------------VFFKPGEQ-----------

**hCaMK1**  ------NLL------YYSLD---E---D--SK--IMISDFGLS--------------------------------------KMEDPGSV-----------

**AtNPH1**  ------NVL------I---Q---G---N--GD--ISLSDFDLS--------------------------------------CLTSCKPQ-----------

**AtPVPKlikePK5**  ------NVL------V---R---E---D--GH--IMLSDFDLSLRCLVSPTLVK---------------------------SAAIESDPLRKNVYCVQPA

**AtS6KlikePK1**  ------NIL------M---D---T---D--GH--VMLTDFGLA--------------------------------------KEFEENTR-----------

**hGRK6**  ------NIL------L---D---D---H--GH--IRISDLGLA--------------------------------------VHVPEGQT-----------

**AtSnRK2**  ------NTL------L---D---G---S--PAPRLKICDFGYS--------------------------------------KSSVLHSQ-----------

**OUTGROUP1**  ------NVF------L---T---KGARG--EQ--ARLLDFGIA--------------------------------------RLVEPDAD-----------

**OUTGROUP2**  ------NIM------I---T---D---D--QI--IKITDFGIA--------------------------------------LSSNEADI-----------

**OUTGROUP3**  ------NIL------M---N---E---N--LT--CKITDFGIA--------------------------------------RAYGDTTL-----------

**OUTGROUP4**  ------NIM------I---S---A---T--NA--VKVMDFGIA--------------------------------------RAIADSGN-----------

**AtTousled**  ------NVL------F---D---E---F--GV--AKVTDFGLS--------------------------------------KIVEDNVG-----------

**hTousledLK1**  ------NIL------LVDGT---A---C--GE--IKITDFGLS--------------------------------------KIMDDDSY-----------

**S08428T0**  ------HIL------L---A---A---N--WD--VKLSGFAFG--------------------------------------HRAGASPV-----------

**S08432T0**  ------HVF------V---S---S---E--WR--AHLGGFMYA--------------------------------------TYVDEP-------------

**Pi03773T0**  ------SVF------V---D---P---S--KG--CKLNPIQAA--------------------------------------LPTDGSSP-----------

**Pr75310**  ------SVF------V---D---P---K--KG--CKLNPVQAA--------------------------------------LPTDGSSP-----------

**Ps136501**  ------TVL------V---D---P---D--KG--CKLNPIQAA--------------------------------------LPTNGSSP-----------

**Pu07161**  ------SVF------V---D---S---E--KG--CKLNTLLAS--------------------------------------VAEDVVPS-----------

**S09505T0**  ------TVF------V---D---S---A--QG--CKLHTLALV--------------------------------------DDGAIDD------------

**AT1G14390_LRRVI-1**  ------NVL------L---D---E---T--LT--VKLSGYSIPLPS-----------------------------------KLLIFSL------------

**AT5G41180_LRRVI-2**  ------AVY------L---T---E---D--FT--PKLVDFECW--------------------------------------KIIQVRSE-----KNL---

**hTGFbRI**  ------NIL------V---K---K---N--GT--CCIADLGLA--------------------------------------VRHDSATD-----TID---

**hTGFbRII**  ------NIL------V---K---N---D--LT--CCLCDFGLS--------------------------------------LRLDPTLS-----VDD---

**Pi14992T0**  ------NVM------V---T---E---L--FH--CKLGDV-----------------------------------------RHHCRDDT-----HHA---

**Ps157753**  ------NVM------V---T---E---L--LR--CKIGDA-----------------------------------------RHHSRDDK-----HHA---

**Pi04971T0**  ------KVL------V---N---E---Q--GE--AKLSAMDILLPSDLSSRKDENNH-TTEDFRGSLRESALWTMQKITGLRPTRSSKA-----MKQQTS

**Ps136072**  ------KVL------V---N---E---Q--GE--AKLSAMDVLLPSDLVPTKKDDNHRMTDDFRGSLRESAMWTMQKITGLRGTRTSKA-----KRNGSG

**Pr93648**  ------KVL------V---N---E---Q--GE--AKLSAMDIMLPTEFVPSKDDDAHRMTDDFRGSLRDSALWTMQKIAGLRPTR---------------

**Pu03658**  ------KVL------V---N---D---H--GE--AKLCALDVKLPNDISNAVERE---------GSIRQSAKIRMKRMMGIKPSSFTQS-----QLG---

**AtCKI1**  ------NFL------M---GLGRR---A--NQ--VYIIDYGLA--------------------------------------KKYKDLQT-----QKH---

**hCKIalpha2**  ------NFL------M---GIGRH---C--NK--LFLIDFGLA--------------------------------------KKYRDNRT-----RQH---

**mCKIalpha**  ------NFL------M---GIGRH---C--NK--LFLIDFGLA--------------------------------------KKYRDNRT-----RQH---

**VIII IX**

**------------- --------**

**610 620 630 640 650 660 670 680 690 700**

**....|....|....|....|....|....|....|....|....|....|....|....|....|....|....|....|....|....|....|....|**

**AT1G06840_LRRVIII-1** -------------------------------------------------------STVVKGTPGYLDPEYF---LTHQ----L-T-DKSDVYSL------

**AT4G29180_LRRI**  -------------------------------------------------------VTAVMGTPGYVDPEYY---NTFK----L-N-EKSDVYSF------

**AT3G14840_LRRVIII-2** -------------------------------------------------------STRVAGTYGYMAPEYA---MRGH----L-T-DKADVYSF------

**AtNIK1_LRRII**  -------------------------------------------------------TTAVRGTVGHIAPEYL---STGQ----S-S-EKTDVFGF------

**AtNAK**  -------------------------------------------------------STRVMGTQGYAAPEYL---ATGH----L-S-VKSDVYSF------

**AT2G24230_LRRVII**  -------------------------------------------------------DEIIHGSPGYLPPEFL---QPEH---ELPT-PKSDVYCF------

**AtBRI1_LRRXb**  -------------------------------------------------------VSTLAGTPGYVPPEYY---QSFR----C-S-TKGDVYSY------

**AtCLV1_LRRXI**  -------------------------------------------------------MSSIAGSYGYIAPEYA---YTLK----V-D-EKSDVYSF------

**AtFEI1_LRRXIIIa**  -------------------------------------------------------TTIVAGTFGYLAPEYM---QSGR----A-T-EKTDVYSF------

**AtER_LRRXIIIb**  -------------------------------------------------------STYVMGTIGYIDPEYA---RTSR----L-T-EKSDVYSY------

**AtRPK1_LRRXV**  -------------------------------------------------------TTGVAGTFGYVAPEYA---MTCR----V-S-EKADVYSY------

**AtTMK1_LRRIX**  -------------------------------------------------------ETRIAGTFGYLAPEYA---VTGR----V-T-TKVDVYSF------

**AT3G28450_LRRXa**  -------------------------------------------------------MTGDLGEFGYVAPEYS---TTML----A-S-LKGDVYGL------

**AtIMK3_LRRIII**  -----------------------------------------------------SSVIATAGALGYRAPELS---KLKK----A-N-TKTDVYSL------

**AT2G45340_LRRIV**  -----------------------------------------------------FSKLKASAAMGYLAPEYI---TTGR----F-T-DKSDVYAF------

**AtSCM_SUB_LRRV**  -----------------------------------------------------------SQMAGYAAPEVE---YGSY------T-CQSDVFSL------

**AtFLS2_LRRXII**  ----------------------------------------------------TASTSAFEGTIGYLAPEFA---YMRK----V-T-TKADVFSF------

**dmPELLE**  ---------------------------------------------------AVVEVNKVFGTKIYLPPEFR---NFRQ----L-S-TGVDVYSF------

**drIRAK1**  ------------------------------------------------KTSTVAQTATVRGTLAYLPEEYL---KDGQ----L-G-VEIDAYSF------

**hIRAK1**  ------------------------------------------------QSSMVARTQTVRGTLAYLPEEYI---KTGR----L-A-VDTDTFSF------

**xtPELLE**  -------------------------------------------------SRTLARTSTVKGTLAYLPEEYV---KMGK----L-T-FELDTYSF------

**AtCTR1_Raf**  -----------------------------------------------------LSSKSAAGTPEWMAPEVL---RDEP----S-N-EKSDVYSF------

**Mb27170**  -----------------------------------------NFLESNSDAISDMTMTKGVGTLLWTSPETL---SGGH----Y-S-LPADVYSF------

**Mb28586**  --QNLSPRQRMLSVAEDSDNEDNDESDNYDESATLLRHVDARRETRRLQSTSVPPVTIRVGTTQFLAPETF---DGRN----Y-S-VHSDVFAY------

**hRaf1**  -------------------------------------------------------VEQPTGSVLWMAPEVIRMQDNNP----F-S-FQSDVYSY------

**Mb37485**  ---------------------------KPSGEDAKATDSVHVRHQSIQNEDTEVTMTTQVGTILWMAPEVW----GGS----Y-N-ASCDVYSFGLSCPI

**Esi0009_0077**  ------------------------------------------------ATYTTKPREMIGMSLPWAAPEVL---NRRG----S-S-FKGDVYSF------

**Esi0009_0083**  ------------------------------------------------ATYTTKPREMVGMSLPWAAPEVL---NRRG----S-S-FEGDVYSF------

**Esi0020_0071**  ------------------------------------------------ATYTTT-KPSTQMSLAWSAPEVL---ESGG----S-T-YASDVYSF------

**Esi0173_0029**  -----------------------------------------------LATYTTNPGPSTHISFAWTAPEVL---EKQT----T-S-KASDVYSF------

**Ch136568**  -------------------------------------------------------VSGVVGTLAWSAPELL---MGQR----C-G-AKADVYSF------

**Ch50123**  -------------------------------------------------------VSGTVGTLAWSAPELL---MGRR----C-G-AKADIFSF------

**Ch137597**  -------------------------------------------------------RSAAGFCCTHAAPEQL---MGYR----C-T-LAADMYGF------

**Ch137605**  ------------------------------------------------------GGGARGFCLTHAAPEQV---LGER----C-T-SAADMYSY------

**Ch56654**  ------------------------------------------------------GGGARGFCLTHAAPEQM---LGQP----C-T-SAADMYSY------

**Ch141610**  -------------------------------------------------------LSAGAFCLTHAAPEQV---LGQR----C-T-LTADIYSL------

**Ch143410**  -------------------------------------------------------LTAAGFCL---------------------T-HAADIYSL------

**Ch141452**  -------------------------------------------------------RSAAGLSLTYAAPEQL---MGLR----C-T-LAADVYSL------

**Mb10450**  ----------------------------DDEGWIALGSLASTVASTGSSQAALMTMSTTAGTPMYMAPECL---LQGK----V-S-PAADVFAF------

**Mb25375**  ----------------------------GSDVASDASLGSVGRDLLVENMEASLTQSGMAGTPLYMAVEVL---ATAC----NGG-LPADVFSF------

**Mb28923**  -------------------------------------PVVLDGEAAMIEIGDRREQSFAVGTPLYMAPEVL---TTGR----NGG-YPADVFAF------

**Mb37923**  ------------------------------------VDAAALAGAISEEQQLETSLSLLAGTPLYLAPEVL---RTGR----NGG-PEADVFAF------

**Mb34096**  -------------------------------------------DASAAVHSANLQYTMSLGTPLYLSPEVL---RGEA----HDG-FKVDVFAF------

**Mb36839**  ----ERRAELKRQRQAARGDHSNTSSGIDAIDLSRSGGALSQASAAPSDPASSRTLTAAVGTPLYCAPEVL---RGDE----Y-H-LPADMYSF------

**Mb36426**  -------------------------------------RDFSTSSSPISTPNASLDKTVMVGTPLYAAPEVL---EGSK----TYD-RFADVWSF------

**hAXL**  -------------------------------------------------------GRIAKMPVKWIAIESL---ADRV----Y-T-SKSDVWSF------

**hHGFR**  -----------------------------------------------------HNKTGAKLPVKWMALESL---QTQK----F-T-TKSDVWSF------

**hRYK**  -------------------------------------------------------GDNENRPVRWMALESL---VNNE----F-S-SASDVWAF------

**hDDR**  -------------------------------------------------------QGRAVLPIRWMAWECI---LMGK----F-T-TASDVWAF------

**hIR**  -------------------------------------------------------GGKGLLPVRWMAPESL---KDGV----F-T-TSSDMWSF------

**hLTK**  -------------------------------------------------------GDRALLPVKWMPPEAF---LEGI----F-T-SKTDSWSF------

**hMuSK**  -------------------------------------------------------NENDAIPIRWMPPESI---FYNR----Y-T-TESDVWAY------

**hTRKalpha**  -------------------------------------------------------GGRTMLPIRWMPPESI---LYRK----F-T-TESDVWSF------

**hKLGlikePTK7**  --------------------------------------------------------RQAWVPLRWMSPEAI---LEGD----F-S-TKSDVWAF------

**hFGFR2**  -------------------------------------------------------TTNGRLPVKWMAPEAL---FDRV----Y-T-HQSDVWSF------

**hRET**  -------------------------------------------------------RSQGRIPVKWMAIESL---FDHI----Y-T-TQSDVWSF------

**hVGFR1**  -------------------------------------------------------KGDTRLPLKWMAPESI---FDKI----Y-S-TKSDVWSY------

**hPDGFRbeta**  -------------------------------------------------------KGSTFLPLKWMAPESI---FNSL----Y-T-TLSDVWSF------

**hTIE1**  -------------------------------------------------------KTMGRLPVRWMAIESL---NYSV----Y-T-TKSDVWSF------

**hEGFR**  -------------------------------------------------------AEGGKVPIKWMALESI---LHRI----Y-T-HQSDVWSY------

**hEPH**  -------------------------------------------------------TQGGKIPIRWTAPEAI---AHRI----F-T-TASDVWSF------

**Pi00640T0**  PGRF---------------------------------------------------MTPGVGTSFWIAPEVL---LGRD----Y-D-ERADIFSF------

**Pi19256T0**  PGRF---------------------------------------------------MTPGVGTSFWIAPEVL---LGRD----Y-D-ERADIFSF------

**Pr72884**  PGRF---------------------------------------------------MTPGVGTSFWIAPEVL---LGRD----Y-D-EHADIFSF------

**Ps140986**  PGRF---------------------------------------------------MTPGVGTSFWIAPEVL---LGRD----Y-D-EHADVFSF------

**Pi00643T0**  -------------------------------------------------------MTVGIGTSFWIAPEVL---LGRD----Y-D-ERADIYSF------

**Pr72888**  -------------------------------------------------------MTVGIGTSFWIAPEVL---LGHD----Y-D-ERADIYSF------

**Ps140988**  -------------------------------------------------------MTVGIGTSFWIAPEVL---LGRD----Y-D-ERADIYSF------

**Pi00646T0**  ---H---------------------------------------------------MTAGIGTSFWIAPEVL---LGKD----Y-D-ERADIFSL------

**Ps140991**  ---H---------------------------------------------------MTAGIGTSFWIAPEVL---LGKD----Y-D-ERADIFSL------

**Pr72890**  ---H---------------------------------------------------MTAGIGTSFWIAPEVL---LGKD----Y-D-ERADIFSL------

**Pi17831T0**  ---H---------------------------------------------------MTAGVGTSFWIAPEVL---CGQD----Y-D-ERADIFSF------

**Ps132644**  ---H---------------------------------------------------MTAGVGTSFWIAPEIL---LGRD----Y-D-ERADIYSF------

**Pi17840T0**  ---H---------------------------------------------------MTAGVGTSFWIAPEVL---LGRD----Y-D-EAADIFSF------

**Pr76005**  ---H---------------------------------------------------MTAGVGTSFWIAPEVL---LGRD----Y-D-EAADIYSF------

**Ps132640**  ---H---------------------------------------------------MTAGVGTSFWIAPEVL---LGRD----Y-D-EAADIYSF------

**Pi17832T0**  ---HP--------------------------------------------------MTAGIGTSFWIAPEVL---LGRD----Y-N-EQADIYSF------

**Pr76010**  ---HA--------------------------------------------------MTAGIGTSFWIAPEVL---LGRD----Y-D-EQADIYSF------

**Ps132643**  ---HA--------------------------------------------------MTAGIGTSFWIAPEVL---LGRD----Y-N-EQADIYSF------

**Pi05112T0**  -------------------------------------------------------MTAGVGTARWIAPEVI---LGGH----Y-T-EFADIYSF------

**Pr82847**  -------------------------------------------------------MTAGVGTARWIAPEVI---LGGH----Y-T-EFADIYSF------

**Ps132270**  -------------------------------------------------------MTAGVGTARWIAPEVI---LGGH----Y-T-EFADIYSF------

**Pu14970**  -------------------------------------------------------MTAGVGTARWIAPEVV---LGGH----Y-T-EAADIYSF------

**S05981T0**  -------------------------------------------------------MTAGVGTCRWMAPEVI---QGAH----Y-D-EAVDIYSF------

**Pr81631**  -------------------------------------------------------MTAGVGTTRWLAPEIA---RGEE---KY-T-EAVDVYSF------

**Pi02124T0**  -------------------------------------------------------MTAGVGTARWVAPEII---LGED----Y-S-ELADIYSL------

**Pr73421**  -------------------------------------------------------MTAGVGTARWVAPEII---LGED----Y-S-ELADIYSL------

**Ps144068**  -------------------------------------------------------MTAGVGTARWVAPEII---LGED----Y-S-ELADIYSL------

**S04928T0**  -------------------------------------------------------MTSNVGTARWIAPEVL---LNDD----Y-S-EKADIYSF------

**Pi07724T0**  -------------------------------------------------------MTGGVGTGRWLAPEVI---RGDT---DY-G-SAADIYSF------

**Ps158080**  -------------------------------------------------------MTGGVGTGRWLAPEVI---RGDT---DY-G-SAADIYSF------

**Pr77475A**  -------------------------------------------------------MTGGVGTGRWLAPEVI---RGDT---DY-G-SAADIYSF------

**Pi07725T0**  -------------------------------------------------------MTAGVGTGRWLAPEVI---RGDT---DY-G-CCADIYSF------

**Pi21299T0**  -------------------------------------------------------MTAGVGTGRWLAPEVI---RGDT---DY-G-CCADIYSF------

**Pr77476**  -------------------------------------------------------MTAGVGTGRWLAPEVI---RGDT---DY-G-CSADIYSF------

**Ps140003**  -------------------------------------------------------MTAGVGTGRWLAPEVI---RGDT---DY-G-CSADIYSF------

**Pi07731T0**  -------------------------------------------------------MTGGVGTGRWLAPEVI---RGDS---DY-G-PAADIYSF------

**Ps139996**  -------------------------------------------------------MTGGVGTGRWLAPEVI---RGDS---DY-G-PAVDIYSF------

**Ps139997**  -------------------------------------------------------MTGGVGTGRWLAPEVI---RGDS---DY-G-PAVDIYSF------

**Pr77470**  -------------------------------------------------------MTGGVGTGRWLAPEVI---RGDA---DY-G-PAADIYSF------

**Pr77475B**  -------------------------------------------------------MTVGVGTGRWLAPEVI---TGDT---NY-G-PPADIYSY------

**Pi09664T0**  -------------------------------------------------------MTAAVGTGRWLAPEVI---AGSG---KY-D-QSVDVFSF------

**Pr75814**  -------------------------------------------------------MTAAVGTGRWLAPEVI---SGSS---KY-D-QSVDVFSF------

**Ps135332**  -------------------------------------------------------MTAAVGTGRWLAPEVI---SGSS---NY-D-QSVDVFSF------

**Ps135334**  -------------------------------------------------------MTAAVGTGRWLAPEVI---SGSS---NY-D-QSVDVFSF------

**Pi23090T0**  -------------------------------------------------------MTIGVGTGRWLAPEII---SGST---EY-D-QRADIFSF------

**Pi23143T0**  -------------------------------------------------------MTIGVGTGRWLAPEII---SGST---EY-D-QRADIFSF------

**Pr81229**  -------------------------------------------------------MTVGVGTGRWVAPEII---SGST---EY-D-QSADIFSF------

**Pr75791**  -------------------------------------------------------MTAGIGTRRWLAPEII---SGSS---DY-D-QSADIFAF------

**Pi09665T0**  -------------------------------------------------------MTRGVGTGRWLAPEVI---TGNR---NY-D-QTSDIFAL------

**Ps135333**  -------------------------------------------------------MTSGVGTGRWLAPEVI---AGNR---DY-D-QTSDIFAL------

**Ps127796**  -------------------------------------------------------KDQQSVMGPWQAPEVL---MGSD---DR-D-PAVDIFSF------

**Pi13397T0**  -------------------------------------------------------MTAGIGTMAWIAPEVL---RGER----Y-T-EMADIYSF------

**Ps140146**  -------------------------------------------------------MTAGIGTMAWIAPEVL---RGER----Y-T-EMADIYSF------

**Pr81472**  -------------------------------------------------------MTAGIGTMAWIAPEVL---RGER----Y-T-EMADIYSF------

**Pi14970T0**  -------------------------------------------------------MTGGMGTIAWIAPEVL---QGDR----Y-S-ESADIFSF------

**Ps138087B**  -------------------------------------------------------MTGGMGTTAWIAPEVL---QGER----Y-S-ERADIFSF------

**Pr75692**  -------------------------------------------------------MTGGMGTTAWIAPEVL---QGER----Y-S-EKADIFSF------

**Pi14971T0**  -------------------------------------------------------MTAEIGTISWIAPEVL---RGER----Y-S-EKADMYSF------

**Pi14972T0**  -------------------------------------------------------MTAEIGTVSWIAPEVL---RGEH----Y-S-EKADVYSF------

**Pr75691A**  -------------------------------------------------------MTAEIGTVSWIAPEVL---RGEH----Y-S-EKADVYSF------

**Ps138088**  -------------------------------------------------------MTAEIGTVSWIAPEVL---KGEH----Y-S-EKADVYSF------

**S06057T0**  -------------------------------------------------------MTAEIGTIAWIAPEIL---KGDR----Y-S-ESADMYSF------

**S02178T0**  -------------------------------------------------------MTAEVGTVPWIAPEVL---KGVR----Y-S-EKADIYSF------

**S02311T0**  -------------------------------------------------------MTSEIGTVAWIAPEVL---KGVR----Y-T-EKADMYSL------

**S02316T0**  -------------------------------------------------------MTAEIGTVAWIAPEVL---KGVY----Y-T-EKADVYSL------

**S13911T0**  -------------------------------------------------------MTSEIGTIPWIAPEVL---KGVR----Y-S-EKADMYSL------

**Pi05340T0**  -------------------------------------------------VSSGGPQTAEVGTAAWIAPEVI---KGAR----Y-D-QKADIYSF------

**Ps133283**  -------------------------------------------------VSSGGPQTAEVGTAAWIAPEVI---KGAR----Y-D-QKADIYSF------

**Ps133286**  -------------------------------------------------VSSGGPQTAEVGTAAWIAPEVI---KGAR----Y-D-QKADIYSF------

**Pr80778**  -------------------------------------------------VSTGGPQTAEVGTAAWIAPEVI---KGGR----Y-D-QKADIYSF------

**S15848T0**  -------------------------------------------------------MTMGTGTTAWAAPEVL---LHDG---HY-N-EKADVYSF------

**S15849T0**  -------------------------------------------------------MTREAGTTAWTAPEVL---LSDG---HY-N-EKADVYSF------

**S15852T0**  -------------------------------------------------------MTREAGTTAWTAPEVL---LSDG---HY-N-EKADVYSF------

**S01258T0**  -------------------------------------------------------MTNGIGTYQWMAPEII---SGTS----Y-S-EAADIYSF------

**S09257T0**  -------------------------------------------------------MTHGIGTYQWMAPEVF---SATN----Y-G-APADIYSF------

**S15543T0**  -------------------------------------------------------MTNGIGTYQWMAPEVI---SATN----Y-G-APADIYSF------

**S09298T0**  -------------------------------------------------------MTNGIGTYQWMAPEVI---MGTE----Y-T-VAADIYSF------

**S15453T0**  -------------------------------------------------------MTNGIGTYQWMAPEII---MGTE----Y-T-IAADVYSF------

**S08727T0**  -------------------------------------------------------LTCGIGTYQWMAPEVI---GGSE----Y-T-VAADVYSL------

**S09308T0**  -------------------------------------------------------MTNGVGSYQWAAPELL---LGSL----Y-N-TAVDIYSF------

**S01416T0**  -------------------------------------------------------MTVGVGTYRWMAPEIL---QFNH----Y-T-TAADIYSF------

**S06128T0**  -------------------------------------------------------MTVGVGTYRWMAPEIL---QFNH----Y-T-VAADIFSF------

**S12836T0**  -------------------------------------------------------MTCGVGTYRWMAPEVL---QDSH----Y-S-VAADVYSF------

**S17043T0**  -------------------------------------------------------MTVGVGTYRWMAPEIL---KEGH----Y-S-VAADIYSF------

**S08987T0**  -------------------------------------------------------MTIGVGTYRWMAPEVL---QECY----Y-S-TSADIYSF------

**S13082T0**  -------------------------------------------------------MTVGVGTYRWMAPEVL---QENH----Y-T-TSADVYSF------

**S05673T0**  -------------------------------------------------------MTNAVGTYRWTAPEVL---KGKH----Y-D-TKADIYSF------

**S09620T0**  -------------------------------------------------------MTNAVGTYRWTAPEVL---KGKH----Y-D-TKADIYSF------

**S12826T0**  -------------------------------------------------------MTLGAGGHRWMAPEVL---HAAD----Y-S-TAVDVFSF------

**S03267T0**  ------------------------------------------------DV-----TSHGFGTSRWMAPEVL---TGDA----Y-T-KAADIYAF------

**S13910T0**  -------------------------------------------------------STDDEAVVAWSAPEVL---KGLR----Y-T-EKADIYAL------

**Pi05338T0**  -------------------------------------------------------SSLSIEDRAWTAPEVL---TSGA----F-D-EKSDVYSF------

**Pr80780**  -------------------------------------------------------SSLSIEDRAWIAPEVL---ASGE----F-D-EKSDIYSF------

**Ps133281**  -------------------------------------------------------SSLSMEDRAWVAPEVL---ASGD----F-D-EKSDVYSL------

**Pi22892T0**  -------------------------------------------------------IDISHRDERWLSPEVI---TGTA---DY-S-PAADIYAF------

**Pr94275**  -------------------------------------------------------VRLSLREERWLPPEVI---TGTA---DY-S-PAADMYAF------

**Ps135323**  -------------------------------------------------------IGISLREERWLPPEVI---TGAA---DY-S-PAADMYAF------

**S01930T0**  -------------------------------------------------------MTQNVGTMQWIAPEML---RGED----Y-C-EAIDVYSF------

**Ps133943**  ------------------------------------------------------MSYSAVGSGRWISPEAL---LGRETSSSY-P-DASDVYSF------

**S03896T0**  ----------------------------------------------------------FVPDRLLTPPEVL---KGEE----W-T-EKADIYAF------

**S09249T0**  ---------------------------------------------------------APSSSFRYMAPELD-----------------------------

**Pi03775T0**  -------------------------------------------------------SPLDNECKIFVAPEIL---RGEQ----P-S-ERSDVYAF------

**Pr75312**  -------------------------------------------------------SPLDNECKIFAAPEVL---RGEQ----P-T-ERSDVYAF------

**Ps136506**  -------------------------------------------------------SPLDNECKVFVAPEVL---RGEQ----P-T-ERSDVYAF------

**AT3G14840_LRRXIV**  -------------------------------------------------------KIARLLRLSQSSQESV---PGSA---ATAT-CAYDVYCF------

**Ps141628**  --------------------------RNDVGSLPSTTGSSARFSGRSNTSSQNAGEEVHMDMTVWTAPEVL---DGRQ----Y-T-PKADIYSF------

**Pu08017**  --------------------------EQ---------GKQASLNKSTSTVSEAAEEEVRLDMTVWTAPEVI---DGQQ----Y-T-TKADMYSF------

**S14923T0**  -------------------------------------------------------TSFGSRTLAFEAPEVL---SKGA----PRG-HATDVFAL------

**S14924T0**  -----------------------------------------------------VHRVYGDGTIAWEAPEVL---MHDC----PRG-GTADVYSL------

**Mb37167**  -------------------------------------------------------TDRTITSLCWCAPELF---AYSD----AAS-PACDVYSY------

**AtAME2**  ---------------------------------NLPKSSAIKLIDFGSTTFEHQDHNYIVSTRHYRAPEVI---LGVG----W-N-YPCDLWSI------

**hCLK1**  -------------------------------------------------TYDDEHHSTLVSTRHYRAPEVI---LALG----W-S-QPCDVWSI------

**AtCDC2a**  ------------------------------------------------------TFTHEVVTLWYRAPEIL---LGSH----HYS-TPVDIWSV------

**hCDK3**  ------------------------------------------------------TYTHEVVTLWYRAPEIL---LGSK----FYT-TAVDIWSI------

**AtMPK1**  ------------------------------------------------------FMTEYVVTRWYRAPELL---LCCD----NYG-TSIDVWSV------

**hMAPK1**  ---------------------------------------------------HTGFLTEYVATRWYRAPEIM---LNSK----GYT-KSIDIWSV------

**AtGSK3b**  -------------------------------------------------------NISYICSRYYRAPELI---FGAT----EYT-SSIDIWSA------

**AtGSK3i**  -------------------------------------------------------NISYICSRYYRAPELI---FGAT----EYT-ASIDIWSA------

**AtCKA1**  -------------------------------------------------------YNVRVVSRYFKGPELL---VDLQ----DYD-YSLDMWSL------

**hCK2a**  -------------------------------------------------------YNVRVASRYFKGPELL---VDYQ----MYD-YSLDMWSL------

**AtMEKK1**  -------------------------------------------------------IKSCKGTPFWMAPEVINRKDSDG----Y-G-SPADIWSL------

**hMAPKKK1**  -----------------------------------------------------EFQGQLLGTIAFMAPEVL---RGQQ----Y-G-RSCDVWSV------

**AtMKK3**  -------------------------------------------------------CATFVGTVTYMSPERI---RNDS----Y-S-YPADIWSL------

**hMAPKK1**  -------------------------------------------------------ANSFVGTRSYMSPERL---QGTH----Y-S-VQSDIWSM------

**AtCPK7**  -------------------------------------------------------FNEIVGSPYYMAPEVL---RRNY------G-PEIDVWSA------

**hCaMK1**  -------------------------------------------------------LSTACGTPGYVAPEVL---AQKP----Y-S-KAVDCWSI------

**AtNPH1**  -------------------------LLIPSIDEKKKKKQQKSQQTPIFMAEPMRASNSFVGTEEYIAPEII---SGAG----H-T-SAVDWWAL------

**AtPVPKlikePK5**  CIEPSCIQPSCTVPTTCFSPRLFSSKSKKDRKPKNDTANQVRPLPELVAEPTDARSMSFVGTHEYLAPEII---KGEG----H-G-SAVDWWTF------

**AtS6KlikePK1**  -------------------------------------------------------SNSMCGTTEYMAPEIV---RGKG----H-D-KAADWWSV------

**hGRK6**  -------------------------------------------------------IKGRVGTVGYMAPEVV---KNER----Y-T-FSPDWWAL------

**AtSnRK2**  -------------------------------------------------------PKSTVGTPAYIAPEIL---LRQE----YDG-KLADVWSC------

**OUTGROUP1**  --------------------------------------------------SAVSQVGMVLGTPEYLSPEQA---VGAR----A-D-ERSDLYCL------

**OUTGROUP2**  -----------------------------------------------------TQTNTIMGSVHYLAPELA---RGNL----A-T-ERSDIYAL------

**OUTGROUP3**  -----------------------------------------------------TQTNQMLGTVYYLSPEQA---RGNV----A-T-AQSDIYSL------

**OUTGROUP4**  ---------------------------------------------------SVTQTAAVIGTAQYLSPEQA---RGDS----V-D-ARSDVYSL------

**AtTousled**  -------------------------------------------------SQGMELTSQGAGTYWYLPPECF---ELNKTP--MIS-SKVDVWSV------

**hTousledLK1**  ------------------------------------------------GVDGMDLTSQGAGTYWYLPPECF---VVGKEPPKI-S-NKVDVWSV------

**S08428T0**  ----------------------------------------------------------LQSVHEDAAPEVH---AGAP----A-T-PASDVYQL------

**S08432T0**  ----------------------------------------------------------KTLLAPNAAPEVL---AGGL----PST-THSDVYLL------

**Pi03773T0**  ------------------------------------------------YSCQSYYRCCDSTSKAFFAPEIL---IGEP----S-R-SSSDMYAF------

**Pr75310**  ------------------------------------------------YSCQSFYQSCDSTMKAFFAPEIL---IGEP----S-R-SSSDMYAF------

**Ps136501**  ------------------------------------------------YSCQSYYRRCDSTSKAFFAPEIL---IGEP----S-R-SSSDMYAF------

**Pu07161**  -------------------------------------------------------RRYGESSRGYFAPEIL---AGEP----S-R-SSSDMFAF------

**S09505T0**  -------------------------------------------------------DQCRTEDRIFLAPEAL---TGCA----I-S-SATDMYAF------

**AT1G14390_LRRVI-1**  ----------------------------------------------------------TSHEIYNLLGEFQ---VGAESPSNEDG-EKEDVYQF------

**AT5G41180_LRRVI-2**  -------------------------------------------------------KNICNEGAICVLPNAM---EHRD----M-D-LQGNIYSF------

**hTGFbRI**  -----------------------------------------------------IAPNHRVGTKRYMAPEVL---DDSINMKHFESFKRADIYAM------

**hTGFbRII**  -----------------------------------------------------LANSGQVGTARYMAPEVL---ESRMNLENVESFKQTDVYSM------

**Pi14992T0**  ----------------------TFVLLVESTAQQSSDSEEEGEDVRFLQPESRNSQDK-GPTLPLVAPEVL---HHNS----R-H-LHADIYSV------

**Ps157753**  ----------------------THVLLVDAPAVQSSDSEEEGEGVRFLQPERRNSQEQAGPMIPLVAPEVL---HHNS----R-H-LHVDIYSV------

**Pi04971T0**  GNLGRSRYANNSGESDVSGVSGVSSVTLDDNHSQSGDNPAFDEASDEFDENSL--KSSGIGSSVVAA-------------------QRDDVYAF------

**Ps136072**  GNLGRSRYANNSGQSDVSGVSAASSVTLD------GDNPAFDEECDEFDENSL--NSSGIGSATVAA-------------------QRDDVYAF------

**Pr93648**  -HLGRSRFENNSGESDVSG---------------TGDNPAFDEESDEFDENSL--KSSGIGSATVAA-------------------QRDDVYAF------

**Pu03658**  --------------------------------------------------SSLLDGGAAFTPTMPAAPLDS---SNPK----YKD-TKSDIYAF------

**AtCKI1**  --------------------------------------------------IPYRENKNLTGTARYASVNTH---LGIE----Q-S-RRDDLESL------

**hCKIalpha2**  --------------------------------------------------IPYREDKNLTGTARYASINAH---LGIE----Q-S-RRDDMESL------

**mCKIalpha**  --------------------------------------------------IPYREDKNLTGTARYASINAH---LGIE----Q-S-RRDDMESL------

**710 720 730 740 750 760 770 780 790 800**

**....|....|....|....|....|....|....|....|....|....|....|....|....|....|....|....|....|....|....|....|**

**AT1G06840_LRRVIII-1** --------------GVVLLELFTG----------M--QP-ITHGKN-------------IVREINIAYE-------------------------------

**AT4G29180_LRRI**  --------------GIVLLELITG----------K--RS-IMKTDD--------GEKMNVVHYVEPFLK-------------------------------

**AT3G14840_LRRVIII-2** --------------GVVALEIVHG----------K--SN-TSSRSK--------ADTFYLLDWVHVLRE-------------------------------

**AtNIK1_LRRII**  --------------GILLLELVTG----------Q--RA-FEFGKA-------ANQKGVMLDWVKKIHQ-------------------------------

**AtNAK**  --------------GVVLLELLSG----------R--RA-IDKNQP--------VVEHNLVDWARPYLTN------------------------------

**AT2G24230_LRRVII**  --------------GVVLFELMTG----------K--KP-IEDDYL-------DEKDTNLVSWVRSLVR-------------------------------

**AtBRI1_LRRXb**  --------------GVVLLELLTG----------K--RP-TDSPDF---------GDNNLVGWVKQHA--------------------------------

**AtCLV1_LRRXI**  --------------GVVLLELIAG----------K--KP-VGEFGE----------GVDIVRWVRNTEEEITQPSD------------------------

**AtFEI1_LRRXIIIa**  --------------GVLVLEVLSG----------K--RP-TDASFI--------EKGLNVVGWLKFLIS-------------------------------

**AtER_LRRXIIIb**  --------------GIVLLELLTR----------R--KA-VDDESN-------------LHHLIMSKTG-------------------------------

**AtRPK1_LRRXV**  --------------GIVLLELISD----------K--RA-LDPSFS------SHENGFNIVSWAHMMLS-------------------------------

**AtTMK1_LRRIX**  --------------GVILMELITG----------R--KS-LDESQP--------EESIHLVSWFKRMYINK-----------------------------

**AT3G28450_LRRXa**  --------------GVVLLELATG----------L--KA-VGGEGF----------KGSLVDWVKQLES-------------------------------

**AtIMK3_LRRIII**  --------------GVIILELLTG----------K--SP-SEALNG-----------VDLPQWVATAVK-------------------------------

**AT2G45340_LRRIV**  --------------GMILLQILSG----------K--SK-ISHLMI------------------------------------------------LQAVES

**AtSCM_SUB_LRRV**  --------------GVVMLELLTG----------R--RP-FDRTRP--------RGHQTLAQWAIPRLHD------------------------------

**AtFLS2_LRRXII**  --------------GIIMMELMTK----------Q--RP-TSLNDE-------DSQDMTLRQLVEKSIGN------------------------------

**dmPELLE**  --------------GIVLLEVFTG----------R--QV-TDRVPE-------NETKKNLLDYVKQQWRQNRMELLEKHLAAPMGKELDMCM--------

**drIRAK1**  --------------GVVMLEVLTG----------R--QA-LEVDSQ--------SKTVFLKDLVTEEEDDG-------RSFSKAKHSRDLS----YGQTA

**hIRAK1**  --------------GVVVLETLAG----------Q--RA-VKTHGA---------RTKYLKDLVEEEAEEAGVALRSTQSTLQAGLAADAW----AAPIA

**xtPELLE**  --------------GVVLLENLTG----------R--KA-IESDSK--------SHTKYLKDLVKEEECKDEEEEEKGASMASGAEAKLAR----VA---

**AtCTR1_Raf**  --------------GVILWELATL----------Q--QP-WGNLNP-----------------------------------------AQVV----AAV--

**Mb27170**  --------------AIVMWEIATR----------K--LP-WSELTR----------------------------------------SWDVA----AAVEE

**Mb28586**  --------------GMVLFHIMTR----------H--AP-LYPMELCIGPSLILR----------------------------------------KAIAS

**hRaf1**  --------------GIVLYELITG----------E--LP-YSHINN----------------------------------------RDQII----FMV--

**Mb37485**  GPAPGCIYRMLAEHGVVLWELITR----------Q--LP-FEYEGF------------------------------------LNLHPTNFF----AEVVR

**Esi0009_0077**  --------------GVVVWECLSR----------E--VP-WKGVAG----------------------------------------VDKLV----LAVTA

**Esi0009_0083**  --------------GVVVWECLSR----------E--VP-WKGVAG----------------------------------------VDKLV----LAVTA

**Esi0020_0071**  --------------GIVVWEVVSG----------E--LP-WANKTR----------------------------------------PREIL----SAVLM

**Esi0173_0029**  --------------GIVVWEVLSR----------Q--LP-WADQAQ---------------------------------------PRDIYL----RVVIH

**Ch136568**  --------------GVVLWEICTG----------K--LP-VRGQLR------------------------------------------------------

**Ch50123**  --------------GVVLWEIATG----------E--MP-QRGQMR------------------------------------------------------

**Ch137597**  --------------GILLVELTTQ----------C--VV-DR----------------------------------------------------------

**Ch137605**  --------------GVLLVELTTR----------Q--AV-HR----------------------------------------------------------

**Ch56654**  --------------GVLLVELTTR----------Q--AV-HR----------------------------------------------------------

**Ch141610**  --------------GILLVELTTQ----------Q--AV-TR----------------------------------------------------------

**Ch143410**  --------------GILLVELTTQ----------Q--AV-LK----------------------------------------------------------

**Ch141452**  --------------GILLVEITTQ----------H--MG-TK----------------------------------------------------------

**Mb10450**  --------------SLILWELHFE----------R--SPDILRELE---------------------------------VPLRGPLFPTLC----RVYRE

**Mb25375**  --------------GVLMWEALTS----------Q--TP-DLLAQE-----------------------------YGGPDKYRGPLLTGLW----RLLDA

**Mb28923**  --------------GVVMWETLTG----------Q--AP-DLCLQE---------------------------------GKRRGPLMTVLS----QLLEA

**Mb37923**  --------------GVLAWETIMA----------A--SP-DLVEQE-------------------------------YGAGSRGPFLTRLL----TLLEA

**Mb34096**  --------------GVLCYETAHQ----------K--VP-DLVGQF---------------------------------GDFRGPFLYRLG----QLLDA

**Mb36839**  --------------GVTMWETATR----------A--LPDLLALTY-------------------------------GDQTPSGPFLSTLL----RTIEE

**Mb36426**  --------------AVLMWEMSAH----------R--IPDLLAHRE-----------------------------AAEGVTFRGPYLSNLA----ACLAD

**hAXL**  --------------GVTMWEIATR----------GQ-TP-YPGVEN-----------------------------------------SEIY----DYLRQ

**hHGFR**  --------------GVLLWELMTR----------GA-PP-YPDVNT-----------------------------------------FDIT----VYLLQ

**hRYK**  --------------GVTLWELMTL----------GQ-TP-YVDIDP-----------------------------------------FEMA----AYLKD

**hDDR**  --------------GVTLWEVLML----------CRAQP-FGQLTD-----------------------------------------EQVIENAGEFFRD

**hIR**  --------------GVVLWEITSL----------AE-QP-YQGLSN-----------------------------------------EQVL----KFVMD

**hLTK**  --------------GVLLWEIFSL----------GY-MP-YPGRTN-----------------------------------------QEVL----DFVVG

**hMuSK**  --------------GVVLWEIFSY----------GL-QP-YYGMAH-----------------------------------------EEVI----YYVRD

**hTRKalpha**  --------------GVVLWEIFTY----------GK-QP-WYQLSN-----------------------------------------TEAI----DCITQ

**hKLGlikePTK7**  --------------GVLMWEVFTH----------GE-MP-HGGQAD-----------------------------------------DEVL----ADLQA

**hFGFR2**  --------------GVLMWEIFTL----------GG-SP-YPGIPV-----------------------------------------EELF----KLLKE

**hRET**  --------------GVLLWEIVTL----------GG-NP-YPGIPP-----------------------------------------ERLF----NLLKT

**hVGFR1**  --------------GVLLWEIFSL----------GG-SP-YPGVQM----------------------------------------DEDFC----SRLRE

**hPDGFRbeta**  --------------GILLWEIFTL----------GG-TP-YPELPM-----------------------------------------NEQFY---NAIKR

**hTIE1**  --------------GVLLWEIVSL----------GG-TP-YCGMTC-----------------------------------------AELY----EKLPQ

**hEGFR**  --------------GVTVWELMTF----------GS-KP-YDGIPA-----------------------------------------SEIS----SILEK

**hEPH**  --------------GIVMWEVLSF----------GD-KP-YGEMSN-----------------------------------------QEVM----KSIED

**Pi00640T0**  --------------GVVLSEIDTD----------D--YP-YWNSGT----------------------------PAQDNPDERRSQEKKIL----EKVAL

**Pi19256T0**  --------------GVVLSEIDTD----------D--YP-YWNSGT----------------------------PAQDNPDERRSQEKKIL----EKVAL

**Pr72884**  --------------GVVLSEIDTD----------D--YP-YWNSGS---------------------------IPGQDNPDERRSQEQKIL----EKVAL

**Ps140986**  --------------GVVLSEIDTD----------D--YP-YWNSGS---------------------------IPAQDDHDERRSQEQKIL----EKVAL

**Pi00643T0**  --------------GVVLSEIDTD----------D--YP-YWNAQH---------------------------------PPQGKAQENEIL----RLVAR

**Pr72888**  --------------GVVLSEIDTD----------D--YP-YWNAQH---------------------------------PPQGKVQENEIL----RLVAR

**Ps140988**  --------------GVVLSEIDTD----------D--YP-YWNAQH---------------------------------PPQGKVQENEIL----RLVAR

**Pi00646T0**  --------------GIVISEIDTE----------D--YP-YWNAKN--------------------------------PPQGGKVEETAIL----RMVAA

**Ps140991**  --------------GIVLSEIDTE----------D--YP-YWNAKN--------------------------------PPQGGKVEETAIL----RMVAA

**Pr72890**  --------------GVVISEIDTE----------D--YP-YWNAKN--------------------------------PPQGGKVEETAIL----RMVAA

**Pi17831T0**  --------------GVVLSEIDTD----------D--YP-YWNDNY-------------------------------PNEPRGKIQEAEIV----ARVAA

**Ps132644**  --------------GVVLSEIDTD----------D--YP-YWNDNN-------------------------------PHAARGKIQEAEIL----SQVAV

**Pi17840T0**  --------------GVVLSEIDTD----------D--YP-YWNDEN--------------------------------PSNRGKIQEAEIL----SLVAE

**Pr76005**  --------------GVVLSEIDTD----------D--YP-YWNDEN--------------------------------KSNRGKIQEAEIL----SLVAA

**Ps132640**  --------------GVVLSEIDTD----------D--YP-FWNDEN--------------------------------KSNRGKIQEAEIL----SLVAE

**Pi17832T0**  --------------GVVLSEIDTD----------D--YP-FWNDAN-------------------------------RDGPRGKLQEADIL----RQVAT

**Pr76010**  --------------GVVLSEIDTD----------D--YP-YWNDAN-------------------------------RDAARGKLQEADIL----RQVAS

**Ps132643**  --------------GVVLSEIDTD----------D--YP-YWNDAN-------------------------------RDGARGKLEEADIL----RQVAK

**Pi05112T0**  --------------GVVLSELDTC----------K--AP-FYDATN---------------------------------TNGGKMQDVTIL----QLVSA

**Pr82847**  --------------GVVLSELDTC----------K--AP-FYDATN---------------------------------TNGGKMQDVTIL----QLVSA

**Ps132270**  --------------GVVLSELDTC----------K--AP-FYDATN---------------------------------TNGGKMQDVTIL----QLVSA

**Pu14970**  --------------GVVLSELDTC----------K--AP-FHDATN---------------------------------TNGAKMQDVTIL----QLVSA

**S05981T0**  --------------GCVLSEMDTC----------N--VP-YFDATH---------------------------------TNGSKLQDHAIM----AGVVQ

**Pr81631**  --------------GIILSELDTH----------Q--LP-FADAKA--------------------------------RGSGAPLNDMAIL----QGVSS

**Pi02124T0**  --------------GVLLTEMDTH----------Q--IP-YQTL---------------------------------------GLEESMIV----QQVAV

**Pr73421**  --------------GVLLTEMDTH----------Q--IP-YQTL---------------------------------------GLEESMIV----QQVAV

**Ps144068**  --------------GVLLTEMDTH----------Q--IP-YQTL---------------------------------------GLEESMIV----QQVAV

**S04928T0**  --------------GVLLSELDTH----------D--VP-YSDV---------------------------------------NLDERVIV----QRVAV

**Pi07724T0**  --------------GALLTELDTN----------K--IP-YSNARG---------------------------------SNGKILSDMTIL----HRVAT

**Ps158080**  --------------GALLTELDTN----------K--IP-YSNARG---------------------------------SNGKILSDMTIL----HRVAT

**Pr77475A**  --------------GALLTELDTN----------K--IP-YSNAKG---------------------------------SNGKILSDMTIL----HRVAT

**Pi07725T0**  --------------GALLTELDTN----------Q--IP-FSNARG---------------------------------SNGKVLTDMTIL----HRVAT

**Pi21299T0**  --------------GALLTELDTN----------Q--IP-FSNARG---------------------------------SNGKVLTDMTIL----HRAAT

**Pr77476**  --------------GALLTELDTN----------Q--IP-YSNARG---------------------------------SNGKILSDMTIL----HRVAT

**Ps140003**  --------------GALLTELDTN----------Q--IP-YSNTRG---------------------------------SNGKILSDMTIL----HRVAT

**Pi07731T0**  --------------GTLLTELDTN----------Q--IP-YDNVRA---------------------------------SNGKIMSDLTIL----HRVAT

**Ps139996**  --------------GTLLTELDTN----------Q--IP-YDNVRA---------------------------------SNGKLMSDLTIL----HRVAT

**Ps139997**  --------------GTLLTELDTN----------Q--IP-YDNVRA---------------------------------SNGKLMSDLTIL----HRVAT

**Pr77470**  --------------GVLLTELDTN----------Q--IP-YDQVRA---------------------------------SNGKIMSDLTIL----HRVAT

**Pr77475B**  --------------GVLLTELDTN----------E--IP-YSSARG---------------------------------ANDQALSDMAIL----HQVAT

**Pi09664T0**  --------------GVVLSEMDTH----------A--IP-YEDVRG---------------------------------ANGNRLNDIAIL----QLVAT

**Pr75814**  --------------GVVLSEMDTH----------T--IP-YDD-----------------------------------------------------LVAT

**Ps135332**  --------------GVVLSEMDTH----------T--IP-YDDVRG---------------------------------ANGNRLNDIAIL----QLVAT

**Ps135334**  --------------GVVLSEMDTH----------T--IP-YDDVRG---------------------------------ANGNRLNDIAIL----QLVAT

**Pi23090T0**  --------------GVLLTEIDTH----------N--LP-YYDAVG---------------------------------PSGNKLEEVAVL----QKVAN

**Pi23143T0**  --------------GVLLTEIDTH----------N--LR-YYDAV-------------------------------------------------------

**Pr81229**  --------------GVLLTEIDTH----------D--LP-YNNAVG---------------------------------PSGNKLEEVAVL----QMVAN

**Pr75791**  --------------GVLLSEFDTH----------A--LP-YDDAVG---------------------------------NNGNALEDVTIL----QMIAS

**Pi09665T0**  --------------GVVMSELDTH----------M--LP-YEDARG---------------------------------AGGNPLADVAIL----QLVAS

**Ps135333**  --------------GVVLSELDTH----------M--LP-YEDARG---------------------------------AGGNPLADVAIL----QLVAS

**Ps127796**  --------------GVLLSELDTH----------A--PP-YNDARS---------------------------------LSGTQLAEAVVL----QMIAA

**Pi13397T0**  --------------GVILSELATC----------I--KP-FDGVTN------------------------------------------ALI----VLKVT

**Ps140146**  --------------GVILSELATC----------I--KP-FEGVTN------------------------------------------ALI----VLKVT

**Pr81472**  --------------GVILSELATC----------I--KP-FDGVTN------------------------------------------ALI----VLKVT

**Pi14970T0**  --------------GITLSEMDTC----------G--HP-YNSHKS----------------------------------EVDALTDAKIA----LLVST

**Ps138087B**  --------------GILLSEMDTC----------G--HP-YNSNRS----------------------------------EENALTDAKIA----LLVST

**Pr75692**  --------------GIVLSEMDTC----------G--HP-YNSNLP----------------------------------DADALTDAKIA----ILVSS

**Pi14971T0**  --------------GVIMTELDTC----------R--RP-YSEGVP----------------------------TEDNRGGNIKHTNARIA----VLVSA

**Pi14972T0**  --------------GVILTELDTC----------R--RP-YSQGIP----------------------------GESNRGGNNKTSNTRIA----VLVSA

**Pr75691A**  --------------GVILTELDTC----------R--RP-YSQGVP----------------------------GESNRGGNNKTSNTRIA----VLVSA

**Ps138088**  --------------GVILTELDTC----------R--RP-YSLGIP----------------------------GESNRGGNNKTSNTRIA----VLVSA

**S06057T0**  --------------GVLLSEVDTG----------E--SP-YGNAVS---------------------------------TNGTALPKPVIA----MMVIE

**S02178T0**  --------------GVAMSEVDLC----------I--VP-YSNVHM----C-S----------------PD-------SQVSLTMAKSRVA----VLVTT

**S02311T0**  --------------GVLISEMDMM----------E--IP-YSNIHR----------------------------LLPESCLGVELAKTRIA----MLVVA

**S02316T0**  --------------GVLLSEMDTL----------E--IP-YADLDA----IQT----------------AN-------GHWNVSAIKARIA----MLVVS

**S13911T0**  --------------GVLLSELDTH----------R--VP-YADFSL----L-V----------------PE-------AGGNVQMTKARIA----MLVVA

**Pi05340T0**  --------------GVVMCELDTC----------T--RP-YALGVA-----------SSHSASGMTSSTSASNASDDEQVKSLLSSNATLA----LAVSE

**Ps133283**  --------------GIVMCELDTC----------T--KP-YTLGVA-----------SSLSASGMTSFVSSSSAT-DEELKSLLSSNAALA----LAVSE

**Ps133286**  --------------GIVMCELDTC----------T--KP-YTLGVA-----------SSLSASGMTSFVSSSSAT-DEELKSLLSSNAALA----LAVSE

**Pr80778**  --------------GVVMCELDTC----------T--RP-YALGVA-----------SSNSASGMTSFVSSSSAKDDEEVKSLLSSNATLA----LAVSE

**S15848T0**  --------------GVVLSELDTW----------H--VP-YAAASS-----------------------------------SQPMSSVQMA----LLVST

**S15849T0**  --------------GVVLSELDTW----------Q--VP-YATTSS-----------------------------------SQPMSSVQMA----LLVST

**S15852T0**  --------------GVVLSELDTW----------Q--VP-YATSSS-----------------------------------SQPMSSVQMA----LLVST

**S01258T0**  --------------GVVLSELSTH----------A--IP-YLGLTN--------------------------------SATGRSATPQFVM----TKVLA

**S09257T0**  --------------GIILSEFATH----------Q--VP-YADKRH--------------------------------PETGKALAQHYVL----QEVRE

**S15543T0**  --------------GIILSEFATH----------Q--VP-YADKRH--------------------------------PETGKALAQHYVL----QEVRE

**S09298T0**  --------------GVILSEFSTH----------A--VP-YGDVKN--------------------------------AITGRAYTQQAIM----SKVTA

**S15453T0**  --------------GVILSEFSTH----------A--VP-YGDVKN--------------------------------AITGRAYTQQAIM----SKVTA

**S08727T0**  --------------GVLLTEFSTH----------L--TP-YSDMIN--------------------------------PHTQKQYNQQYLL----TQVAS

**S09308T0**  --------------GVLLTEFSTH----------Q--VP-YVRTLD--------------------------------ASTGRVYTQDKVM----QLVTK

**S01416T0**  --------------GMLLSEFDTH----------D--IP-YANMKN--------------------------------EKSGKPLIDTAIM----GMVIA

**S06128T0**  --------------GMLLSEFDSH----------Q--IP-YAGVIN--------------------------------EKNGKPLVDTAIM----SMVIA

**S12836T0**  --------------GMIIAEMGTH----------M--TP-YADLKN---------------------------------AKGQPVVDTAIM----AMLIQ

**S17043T0**  --------------GILLSELDTH----------K--IP-YAESRH--------------------------------PITHKPLVDTAIM----SMVIN

**S08987T0**  --------------GMVLSELSTH----------D--IP-YADVRS---------------------------------ENGHPLVDTAIM----SRVIQ

**S13082T0**  --------------GILISELCTH----------H--IP-YTDKQT---------------------------------ERGNPLVDTAII----NRVIQ

**S05673T0**  --------------GMILTELDTH----------A--VP-YADMVN---------------------------------DRGQALGNFTIM----YKVMQ

**S09620T0**  --------------GMILTELDTH----------A--VP-YADMVN---------------------------------DRGQALGNFTIM----YKVMQ

**S12826T0**  --------------GMVLTECDSH----------R--AP-YDREMD--------------------------------PDTGKLLAEVAIA----RRVSM

**S03267T0**  --------------GIVLSELDSH----------K--IP-FANERT---------------------------------ASGNDLTNLAIL----QRVVN

**S13910T0**  --------------GVLIATMDTG----------K--AP-FANETN-----------------------------------QLRASRTRIA----VHVMA

**Pi05338T0**  --------------GVVMSELDRC----------K--PP-FSRKDS------------------------------------------RRQ----SVDGG

**Pr80780**  --------------GVVLSELDRC----------K--LP-FSRKAS------------------------------------------RRL----SVEDG

**Ps133281**  --------------GVVMSELDRC----------K--LP-YSRKAS------------------------------------------RRQ----SVDDA

**Pi22892T0**  --------------GVILSEIDTH----------S--AP-YENTG----------------------------------SGRQNLSDVEIL----DLVAS

**Pr94275**  --------------GVILSEIDTH----------S--LP-YENIQG-------------------------------VVSGRQSMSDVEIL----DLVAS

**Ps135323**  --------------GVILSEIDTH----------S--LP-YENIQG-------------------------------VVSGRQNMSEVEIL----DLVAS

**S01930T0**  --------------GVVLTELATH----------A--IP-FRDYAH------------------------------------QEDPKRVLV----QELMT

**Ps133943**  --------------GILIAEMDSH----------E--LP-FSDLMQ---------------------------------ANRSAVPETDIL----QLIAK

**S03896T0**  --------------GILICEVDLG----------H--HP-YAHTIK---------------------------------SGDAEDASQQIA----TLVMA

**S09249T0**  ----------------------TH----------D--VP-YGHWHD-----------------DVNDEDEDDDEDKATTTHDQAEEDKRLM----QRIMD

**Pi03775T0**  --------------GVVLVEIDTG----------E--TP-VMNARR---------------------------------LSMERAGREDGL----SPTAK

**Pr75312**  --------------GVVLVEIDTG----------E--TP-VMNARR---------------------------------LSMERSGREDAL----SPTAK

**Ps136506**  --------------GVVLVEIDTG----------E--TP-VMNARK---------------------------------LSMERSGREDVL----SPTAK

**AT3G14840_LRRXIV**  --------------GKILLELITG----------K--LG-ISSCKE----------------------------------TQFKKILTEIM----PYISS

**Ps141628**  --------------GVLLAQLATY----------E---C-TSAEHS--------------------------------------VMDDTEV----PMLNN

**Pu08017**  --------------GILLSQLDTY----------E--FP-VDALHR--------------------------------------V--DSEV----AILGS

**S14923T0**  --------------GVILSSIATR----------R--HP-YQSAID---------------------------------RLGHVGSDVEIL----CR--L

**S14924T0**  --------------GVVMGEIVTR----------T--RP-FQRYVD---------------------------------ALGFVRADVAIL---------

**Mb37167**  --------------GILCWEILQA----------ST-PP-YMDLDK----------------------------------------PWKIR----DFVQG

**AtAME2**  --------------GCILVELCSG----------E--AL-FQTHEN----LEHLAMMERVLGPLPPHMVLRADRRSEKYFRRGAKLDWPEG---------

**hCLK1**  --------------GCILIEYYLG----------F--TV-FPTHDS----KEHLAMMERILGPLPKHMIQKTRKRKYFHHDRLDWDEHSSA---------

**AtCDC2a**  --------------GCIFAEMISQ-------------KPLFPGDSE----IDQLFKIFRIMGTPYEDTWRGVTSLPDY----------------------

**hCDK3**  --------------GCIFAEMVTR----------K--AL-FPGDSE----IDQLFRIFRMLGTPSEDTWPGVTQLPDY----------------------

**AtMPK1**  --------------GCIFAELLGR-------------KPIFQGTEC----LNQLKLIVNILGSQREEDLEFIDNPKAKRYI-------------------

**hMAPK1**  --------------GCILAEMLSN-------------RPIFPGKHY----LDQLNHILGILGSPSQEDLNCIINLKARNYL-------------------

**AtGSK3b**  --------------GCVLAELLLG-------------QPLFPGENS----VDQLVEIIKVLGTPTREEIRCMNPNYT-----------------------

**AtGSK3i**  --------------GCVLAELLLG-------------QPLFPGENS----VDQLVEIIKVLGTPTREEIRCMNPNYT-----------------------

**AtCKA1**  --------------GCMFAGLLFR----------K--EPFFYGHDN----QDQLVKIAKGVGTDELNAYLNKYQLELDPQL-------------------

**hCK2a**  --------------GCMLASMIFR----------K--EPFFHGHDN----YDQLVRIAKVLGTEDLYDYIDKYNIELDPRF-------------------

**AtMEKK1**  --------------GCTVLEMCTG----------Q--IP-YSDLEP----VQALFRIGRGT---------------------------------------

**hMAPKKK1**  --------------GCAIIEMACA----------K--PP-WNAEKH----SNHLALIFKI----------------------------------------

**AtMKK3**  --------------GLALFECGTG----------E--FP-YIANEG---PVNLMLQILDDP---------------------------------------

**hMAPKK1**  --------------GLSLVEMAVG----------R--YP-IPPPDAKELELMFGCQVEGDAAETPPRPRTPGRPLSSYGMDSRPPMAIFELLDYIVNEPP

**AtCPK7**  --------------GVILYILLCG----------V--PP-FWAETE----QGVAQAIIRSVIDFKRDPWPR-----------------------------

**hCaMK1**  --------------GVIAYILLCG----------Y--PP-FYDENG----AKLFEQILKAEYEFDSPYWDD-----------------------------

**AtNPH1**  --------------GILMYEMLYG----------Y--TP-FRGKTR----QKTFTNVLQKDLKFPASIP-------------------------------

**AtPVPKlikePK5**  --------------GIFLYELLFG----------R--TP-FKGSGN----RQTLFNVVGQPLRFPETPV-------------------------------

**AtS6KlikePK1**  --------------GILLYEMLTG----------K--PP-FLGSKG-----KIQQKIVKDKIKLPQF---------------------------------

**hGRK6**  --------------GCLLYEMIAG----------Q--SP-FQQRKK----KIKREEVERLVKEVPEEYSER-----------------------------

**AtSnRK2**  --------------GVTLYVMLVG----------A--YP-FEDPQEPRDYRKTIQRILSVTYSIPEDLH-------------------------------

**OUTGROUP1**  --------------GVLAYRVLSG----------Q--LP-FAGPSP----RQFIAQHASAAPLPLDRAAPG-----------------------------

**OUTGROUP2**  --------------GIILFELLTG----------D--VP-FKGEGA----VNIALQHLETEMPSLKDVIPE-----------------------------

**OUTGROUP3**  --------------GILIFEMITG----------Q--IP-FKGESA----VAIALKHLQEELPDIDKYREN-----------------------------

**OUTGROUP4**  --------------GCVLYEVLTG----------E--PP-FTGDSP----VSVAYQHVREDPIPPSARHEG-----------------------------

**AtTousled**  --------------GVLFYQMLFG----------K--RP-FGHDQS----QERILREDTIIKAK------------------------------------

**hTousledLK1**  --------------GVIFFQCLYG----------R--KP-FGHNQS----QQDILQENTILKAT------------------------------------

**S08428T0**  --------------GRLMNRLGLD-------------AP-------------------------------------------------------------

**S08432T0**  --------------GQLLVDLEVA----------------------------------------------------------------------------

**Pi03773T0**  --------------GVLLAHLDTC----------Q--TA-DEMIRSSWRMRTHIGDFDADNGTLLSTDGTVVSTNVS--DSDSSRRRTTHL----DSLPS

**Pr75310**  --------------GVLLAHLDTC----------Q--TA-DEMIRSSWRMRTHIGDFDTDNGTLLSTDGTVVSTNIGS-DSDSSRRRTTQL----ESLPS

**Ps136501**  --------------GVLLAHIDTC----------Q--TA-DEMIRSSWRMRTQLGDSDSDNGTLLSTDGTVLSTNAGGIDFDSSRRRTTQL----ESLPS

**Pu07161**  --------------GVLLAQLDTC----------Q--TA-DEMIRNSWRMRSMIEPNPISTTIVLGMEGSHRATNSTVDESNS-----------------

**S09505T0**  --------------GVLLMLLDAG----------V--TP-WQLSRRTW----------------------------------------------------

**AT1G14390_LRRVI-1**  --------------GVILIQIITG----------K--VI-AAASSELGSLKLQLENSLR-----------------------------------------

**AT5G41180_LRRVI-2**  --------------GILLLEIVSG-------------RPSYCQDRG------------------------------------------------------

**hTGFbRI**  --------------GLVFWEIARRCSIGGIHEDYQ--LP-YYDLVP------------------------------------SDPSVEEMR----KVVCE

**hTGFbRII**  --------------ALVLWEMTSRCNAVGEVKDYE--PP-FGSKVR------------------------------------EHPCVESMK----DNVLR

**Pi14992T0**  --------------GILLLELWFH----------A--HI-FFQNDRPNNEFEVRNQTKNLRLSIDDRDGAKKRF----QVATLPRTASALQ----KNKEH

**Ps157753**  --------------GILLLELWFH----------S--YI-FFQNDRPNDEFEVRSRPKKLTLSMDNHEDESKSAGASKEDSSLPRTASAVQ----KGKLH

**Pi04971T0**  --------------GTFLWELDTM------------------------------------------------------IAVEEDLASSRIP----AGA--

**Ps136072**  --------------GTFLWELDTM------------------------------------------------------IAVEEDLASSRVG----AGA--

**Pr93648**  --------------GTFLWELDTM------------------------------------------------------IAVEEDLASSRIP----ASA--

**Pu03658**  --------------GLLLWELDTM-------------LS---------------------------------------VDIMKDLATAAEI---------

**AtCKI1**  --------------GYVLMYFLRG----------S--LP-WQGLKA-------------------------GTKKQKYDKISEKKMLTSVE----TLCKS

**hCKIalpha2**  --------------GYVLMYFNRT----------S--LP-WQGLKA-------------------------ATKKQKYEKISEKKMSTPVE----VLCKG

**mCKIalpha**  --------------GYVLMYFNRT----------S--LP-WQGLKA-------------------------ATKKQKYEKISEKKMSTPVE----VLCKG

**X XI**

**-------- -----------**

**810 820 830 840 850 860 870 880 890 900**

**....|....|....|....|....|....|....|....|....|....|....|....|....|....|....|....|....|....|....|....|**

**AT1G06840_LRRVIII-1** ---------------------------------SGSILSTVDKRM------SSVPDE----CLE--KFATLALRCCREE-TDARP-----SMAE------

**AT4G29180_LRRI**  ---------------------------------MGDIDGVVDPRLHGDFSSN---------SAW--KFVEVAMSCVRDR-GTNRP-----NTNQ------

**AT3G14840_LRRVIII-2** ---------------------------------QNTLLEVVDPRLGTDYNKQ---------EAL--MMIQIGMLCTSPA-PGDRP-----SMST------

**AtNIK1_LRRII**  ---------------------------------EKKLELLVDKELLKKKSYDEI-------ELD--EMVRVALLCTQYL-PGHRP-----KMSE------

**AtNAK**  ---------------------------------KRRLLRVMDPRLQGQYSLT---------RAL--KIAVLALDCISID-AKSRP-----TMNE------

**AT2G24230_LRRVII**  ---------------------------------KNQASKAIDPKIQETGSEE---------QME--EALKIGYLCTADL-PSKRP-----SMQQ------

**AtBRI1_LRRXb**  ---------------------------------KLRISDVFDPELMKEDPALEIELL---------QHLKVAVACLDDR-AWRRP-----TMVQ------

**AtCLV1_LRRXI**  ---------------------------------AAIVVAIVDPRL------TGYPLT----SVI--HVFKIAMMCVEEE-AAARP-----TMRE------

**AtFEI1_LRRXIIIa**  ---------------------------------EKRPRDIVDPNC------EGMQME----SLD--ALLSIATQCVSPS-PEERP-----TMHR------

**AtER_LRRXIIIb**  ---------------------------------NNEVMEMADPDITSTCKDLG--------VVK--KVFQLALLCTKRQ-PNDRP-----TMHQ------

**AtRPK1_LRRXV**  ---------------------------------QGKAKEVFTTGLWETGPPD---------DLV--EVLHLALKCTVDS-LSIRP-----TMKQ------

**AtTMK1_LRRIX**  ---------------------------------EASFKKAIDTTIDLDEETLA--------SVH--TVAELAGHCCARE-PYQRP-----DMGH------

**AT3G28450_LRRXa**  ---------------------------------SGRIAETFDENIRGKGHDE---------EIS--KFVEIALNCVSSR-PKERW-----SMFQ------

**AtIMK3_LRRIII**  ---------------------------------EEWTNEVFDLELLNDVNTMGDEIL---------NTLKLALHCVDAT-PSTRP-----EAQQ------

**AT2G45340_LRRIV**  ---------------------------------GRLNEDFMDPNLRKNFPEV---------EAA--QLARLGLLCTHES-SNQRP-----SMED------

**AtSCM_SUB_LRRV**  ---------------------------------IDALTRMVDPSLHGAYPMK---------SLS--RFADIISRSLQME-PGFRP-----PISE------

**AtFLS2_LRRXII**  ---------------------------------GRKGMVRVLDMELGDSIVSLKQEE----AIE--DFLKLCLFCTSSR-PEDRP-----DMNE------

**dmPELLE**  ------------------------------------------------------------------CAIEAGLHCTALD-PQDRP-----SMNA------

**drIRAK1**  ---------------------------------EHICKKHLDRRLTAKDAPATHGSA---------DIARLACQCLERR-RKKRP-----RMTD------

**hIRAK1**  ---------------------------------MQIYKK---------------------------HLGQLACCCLHRR-AKRRP-----PMTQ------

**xtPELLE**  ---------------------------------ARICQYHLDFRVWQHAKE----------VTQ--ELSQLACRCLGRQ--KKRP-----NMQE------

**AtCTR1_Raf**  ---------------------------------GFKCKRLEIP------------RN----LNP--QVAAIIEGCWTNE-PWKRP-----SFAT------

**Mb27170**  ---------------------------------GR---RPTVP------------AV----PVK--AFTDLMQACWDQE-PSARP-----TFAQ------

**Mb28586**  ---------------------------------GL---RPELP------------EAYRHVFPT--SIKCLMEACWVAC-REDRP-----SFPE------

**hRaf1**  ---------------------------------GRGYASPDLSKLY---------KN----CPK--AMKRLVADCVKKV-KEERP-----LFPQ------

**Mb37485**  ---------------------------------PQR--RPTIPSEVGLRSAG---------FPA--DYHKLMRRCWEED-PRLRP-----NMTA------

**Esi0009_0077**  ---------------------------------GE---RPPIP------------EN----APH--DIATLAEACWVHD-PAARP-----TFKK------

**Esi0009_0083**  ---------------------------------GE---RPPIP------------EN----APH--DIAALAEACWVHD-PAARP-----TFKK------

**Esi0020_0071**  ---------------------------------GA---RPSFR------------HD----APA--DIVEIAKACWVGE-PRART-----TFDA------

**Esi0173_0029**  ---------------------------------GD---RPALP------------AD----APV--DIADMLLGCWAQE-PTERP-----TFQA------

**Ch136568**  --------------------------------------DPKVP------------EE----CPA--EVVQLMYRCLSPD-PALRP-----TSTE------

**Ch50123**  --------------------------------------DP------------------------------------------------------------

**Ch137597**  ---------------------------------RGEWRLPRVP------------DE----CPQ--AVLELIEECVVAD-PLRRC-----TAAE------

**Ch137605**  ---------------------------------RGTWHLPHAP------------EE----CPQ--GVLALIEACLSHA-PRARP-----TAAE------

**Ch56654**  ---------------------------------RRAWRLPHAP------------EE----CPQ--GVLALIEACLSHD-PGARP-----TAAE------

**Ch141610**  ---------------------------------RGGWRLPLAP------------QE----CSP--AVLALIERCTAAD-PLQRP-----PATQ------

**Ch143410**  ---------------------------------RGAWRLPLAP------------QE----CSP--AVLALIERCTAAD-PLQRP-----STAQ------

**Ch141452**  ---------------------------------RGEWRPPHVP------------QE----CSQ--EVLELIQACIAPS-PQRRP-----TAAQ------

**Mb10450**  ---------------------------------GR---HLPLEG-----------------MVD--WYQRLCAACMSLE-PADRP-----SFDS------

**Mb25375**  ---------------------------------GK---RLDPAAV-----------------------------------TSARP-----DAPD------

**Mb28923**  ---------------------------------GK---RLDTDSVLTRCPD----------VPK--TVVTLMHACWTLD-PTTRP-----TLKL------

**Mb37923**  ---------------------------------GK---RLEFGPL----------------APT--PLVALTRQCWHLD-PASRP-----TLDH------

**Mb34096**  ---------------------------------GH---RLQLDTDACD-------------VPV--GLANLSLSCMAGP-MHERP-----TFDV------

**Mb36839**  ---------------------------------GH---QLILDPE----------MD----VPD--GYADLMERCLQRD-PALRP-----TCEE------

**Mb36426**  ---------------------------------GH---RLPMD------------ES----WPA--SWRSTMTLCWAQE-PELRP-----SFAE------

**hAXL**  ---------------------------------GN---RLKQP------------AD----CLD--GLYALMSRCWELN-PQDRP-----SFTE------

**hHGFR**  ---------------------------------GR---RLLQP------------EY----CPD--PLYEVMLKCWHPK-AEMRP-----SFSE------

**hRYK**  ---------------------------------GY---RIAQP------------IN----CPD--ELFAVMACCWALD-PEERP-----KFQQ------

**hDDR**  ---------------------------------QGRQVYLSRP------------PA----CPQ--GLYELMLRCWSRE-SEQRP-----PFSQ------

**hIR**  ---------------------------------GG---YLDQP------------DN----CPE--RVTDLMRMCWQFN-PKMRP-----TFLE------

**hLTK**  ---------------------------------GG---RMDPP------------RG----CPG--PVYRIMTQCWQHE-PELRP-----SFAS------

**hMuSK**  ---------------------------------GN---ILSCP------------EN----CPV--ELYNLMRLCWSKL-PADRP-----SFTS------

**hTRKalpha**  ---------------------------------GR---ELERP------------RA----CPP--EVYAIMRGCWQRE-PQQRH-----SIKD------

**hKLGlikePTK7**  ---------------------------------GK--ARLPQP------------EG----CPS--KLYRLMQRCWALS-PKDRP-----SFSE------

**hFGFR2**  ---------------------------------GH---RMDKP------------AN----CTN--ELYMMMRDCWHAV-PSQRP-----TFKQ------

**hRET**  ---------------------------------GH---RMERP------------DN----CSE--EMYCLMLQCWKQE-PDKRP-----VFAD------

**hVGFR1**  ---------------------------------GM---RMRAP------------EY----STP--EIYQIMLDCWHRD-PKERP-----RFAE------

**hPDGFRbeta**  ---------------------------------GY---RMAQP------------AH----ASD--EIYEIMQKCWEEK-FEIRP-----PFSQ------

**hTIE1**  ---------------------------------GY---RMEQP------------RN----CDD--EVYELMRQCWRDR-PYERP-----PFAQ------

**hEGFR**  ---------------------------------GE---RLPQP------------PI----CTI--DVYMIMVKCWMID-ADSRP-----KFRE------

**hEPH**  ---------------------------------GY---RLPPP------------VD----CPA--PLYELMKNCWAYD-RARRP-----HFQK------

**Pi00640T0**  ---------------------------------GSL--RPTFY------------ND----CPP--GVLALASRCLEGR-PTNRP-----SASE------

**Pi19256T0**  ---------------------------------GSL--RPTFY------------ND----CPP--GVLALASRCLEGR-PTNRP-----SASE------

**Pr72884**  ---------------------------------GSL--RPTFY------------ND----CPP--GILALAASCLEGR-PENRP-----SASE------

**Ps140986**  ---------------------------------GSL--RPTFY------------ND----CPP--GVLALAASCLEGR-PENRP-----SAAE------

**Pi00643T0**  ---------------------------------GAK--RPVFS------------DD----CPP--AILELAARCLRAD-PNERP-----SASD------

**Pr72888**  ---------------------------------GAK--RPAFS------------DD----CPP--AILELAARCLRAD-PKERP-----SASE------

**Ps140988**  ---------------------------------GAK--RPAFS------------DD----CPP--AILELAARCLRAD-PEERP-----SASE------

**Pi00646T0**  ---------------------------------GEL--IPDFT------------EN----CPT--AVLELAKACLSLN-PDDRP-----SATE------

**Ps140991**  ---------------------------------GEL--IPDFT------------ES----CPK--AVLELAKACLSLN-PDDRP-----SATE------

**Pr72890**  ---------------------------------GEL--IPDFT------------ED----CPK--AILQLAKACLAVN-PDDRP-----SATE------

**Pi17831T0**  ---------------------------------GHI--RPEFS------------AN----CPE--EILAIADACLQRD-PRDRP-----TAAD------

**Ps132644**  ---------------------------------GHM--RPEFS------------VD----CPE--EILALADACLQKD-PRNRP-----TAAE------

**Pi17840T0**  ---------------------------------GKL--RPSFS------------KD----CPK--AILDLADCCLQKK-PEARP-----TAAE------

**Pr76005**  ---------------------------------GEL--RPDFS------------GD----CPK--AILDLADCCLQKN-PRDRP-----TASQ------

**Ps132640**  ---------------------------------GKL--RPSFS------------SD----CPK--AILDLADCCLQKD-PGDRP-----TAAE------

**Pi17832T0**  ---------------------------------GNK--RPHFS------------TN----CPA--GIMLLAESCLQEN-PDDRP-----TATA------

**Pr76010**  ---------------------------------GTK--RPQFS------------ST----CPA--GILLLAEKCLQKK-PDDRP-----TASE------

**Ps132643**  ---------------------------------GNK--RPQFS------------PN----CPA--AIVLLAESCLQKD-PKDRP-----TAAN------

**Pi05112T0**  ---------------------------------GKL--QPSFS------------DS----CPP--SIVKLARACLSFD-PAQRP-----SAIH------

**Pr82847**  ---------------------------------GKL--QPSFD------------ES----SPP--SIVKLARACLSFD-PAQRP-----SAIH------

**Ps132270**  ---------------------------------GKL--QPSFE------------ES----CPP--SIVKLARACLSFD-PAQRP-----SAIH------

**Pu14970**  ---------------------------------GKL--QPSFT------------DL----CPP--AILKLARACLEFD-PARRP-----NAIH------

**S05981T0**  ---------------------------------GTL--KPSFT------------KD----CPK--EIVAIAVACLETD-PKKRP-----TSTQ------

**Pr81631**  ---------------------------------GEL--QVTLS------------AE----CPR--NLQALARSCMNLD-SNQRP-----TAAQ------

**Pi02124T0**  ---------------------------------GKL--RPKVS------------DT----CPE--IIRRLTHECLQYD-PSLRP-----SAAR------

**Pr73421**  ---------------------------------GKL--RPKVS------------DT----CPE--IIRRLTHECLQYD-PSLRP-----SAAR------

**Ps144068**  ---------------------------------GKL--RPKVS------------DT----CPE--IIRRLTHECLQYD-PSLRP-----STAR------

**S04928T0**  ---------------------------------GQL--RPKFS------------AN----CPE--KLRRLANHCMQAD-PALRP-----DANK------

**Pi07724T0**  ---------------------------------GKL--HPEVG------------SD----CLP--ALKDLVERCLVAD-PTKRP-----AASI------

**Ps158080**  ---------------------------------GKL--HPQVG------------SD----CSP--ALKDLVERCLVED-QAQRP-----AAAV------

**Pr77475A**  ---------------------------------GKL--HPQLE------------SD----CSP--ALRDLVERCFTED-QSQRP-----AAPI------

**Pi07725T0**  ---------------------------------GKL--HPQVR------------PT----CNA--SMKELVERCLVEN-PSDRP-----AATV------

**Pi21299T0**  ---------------------------------GKL--HPQVW------------PT----CNA--SMKELVERCLVEN-PSDRP-----AATV------

**Pr77476**  ---------------------------------GKL--HPQVR------------SS----CGA--SMRDLVNRCLIED-PTKRP-----AATV------

**Ps140003**  ---------------------------------GKL--HPKVR------------ST----CNA--SMKALVERCLVED-PTKRP-----AATV------

**Pi07731T0**  ---------------------------------GKL--HPKVR------------AT----CGK--ALKDLVERCLLED-PAERP-----TAPA------

**Ps139996**  ---------------------------------GRL--HPKLR------------ST----CGK--ALRDLVERCLLED-PAKRP-----TAPA------

**Ps139997**  ---------------------------------GRL--HPKLR------------ST----CGK--ALRDLVERCLLED-PAKRP-----TAPA------

**Pr77470**  ---------------------------------GKL--HPKVR------------AT----CGV--SLRDLIERCLDED-PSRRP-----SAPG------

**Pr77475B**  ---------------------------------AKL--RPVVG------------NR----CPP--PLRDLVDRCLVHD-PSKRP-----TGTV------

**Pi09664T0**  ---------------------------------GQL--RPRFG------------AS----CPP--ELRMLAERCLDQD-PEKRP-----PAHV------

**Pr75814**  ---------------------------------GQL--RPRFG------------AS----CPP--ELRMLAERCLAQD-PTDRP-----PAHV------

**Ps135332**  ---------------------------------GQL--RPRFG------------AS----CPP--ELRMLAERCLAQD-PEERP-----PAHV------

**Ps135334**  ---------------------------------GQL--RPRFG------------AS----CPP--ELRMLAERCLAQD-PEERP-----PAHV------

**Pi23090T0**  ---------------------------------DQL--RPTIS------------AS----CPP--TVRDLAILCMSHS-PQSRP-----LAAE------

**Pi23143T0**  -----------------------------------C--NGVRS------------TK----C--------------------------------------

**Pr81229**  ---------------------------------GQL--RPTIS------------AS----CPS--TVRDLANLCMSQD-SRDRP-----VAAE------

**Pr75791**  ---------------------------------GNL--RPTFS------------DT----CPP--VIFELAKQCMSQE-PRDRP-----PALK------

**Pi09665T0**  ---------------------------------GKL--LPTFS------------SM----CPA--ELHDLARRCMAFN-PHERP-----TAVE------

**Ps135333**  ---------------------------------GRL--LPTFG------------PQ----CPP--ELQELARRCMAYD-PQARP-----TAVE------

**Ps127796**  ---------------------------------GSI--RLRFG------------ED----CPS--DLRSLADSCLAKN-PRVRP-----AASE------

**Pi13397T0**  ---------------------------------SEE--KPDLG------------QN----CPE--DIRELADRCLSFN-ANDRP-----SASV------

**Ps140146**  ---------------------------------SEE--KPDLG------------TN----CPE--DIRELADRCLSFN-PNDRP-----SASV------

**Pr81472**  ---------------------------------SEE--KPDLG------------MN----CPE--DIRELADRCLSFN-ANDRP-----SASV------

**Pi14970T0**  ---------------------------------NAI--KPTIE------------DD----CPP--EIRDVILKCVSFN-ASDRP-----TAVA------

**Ps138087B**  ---------------------------------DAI--KPTIE------------DD----CPP--EIRDLILQCVSFN-ADERP-----TAVA------

**Pr75692**  ---------------------------------DAI--KPTIE------------DD----CPP--EIREVILRCVAFD-PASRP-----SAVA------

**Pi14971T0**  ---------------------------------GKL--RPSLS------------PD----CPN--SVKDLVAKCLDAD-PMNRP-----SALQ------

**Pi14972T0**  ---------------------------------GTL--RPEVH------------VD----CPR--SVRSLVDKCLAFD-PEDRP-----SALQ------

**Pr75691A**  ---------------------------------GTL--RPEVH------------AD----CPR--SVRNLVDMCLAFD-PEDRP-----SALQ------

**Ps138088**  ---------------------------------GSL--RPEVH------------TD----CPR--SVRNLVDKCLAFD-PEDRP-----SALQ------

**S06057T0**  ---------------------------------GEL--RPSFT------------DE----CPP--EVLSIAQRCLDND-PRQRP-----SALD------

**S02178T0**  ---------------------------------GQL--RPSFS------------HT----CPP--AILDIARRCLAYY-PEDRP-----SAAE------

**S02311T0**  ---------------------------------GEL--RPVFS------------TD----CPA--PILNVARRCLAYL-PEDRP-----TARE------

**S02316T0**  ---------------------------------GEI--RPSFS------------AD----VPR--CIADVADRCLAYE-PTARP-----TIRD------

**S13911T0**  ---------------------------------GDL--RPTFS------------PS----CPP--SLLAIATKCLAYD-QTARP-----TANQ------

**Pi05340T0**  ---------------------------------NGV--MPAFH------------SD----CPR--AILDLARMCLSYD-PGDRP-----TAKE------

**Ps133283**  ---------------------------------KGV--TPAFH------------SD----CPR--AILDLARMCLSYD-PEDRP-----TAKE------

**Ps133286**  ---------------------------------KGV--TPAFH------------SD----CPR--AILDLARMCLSYD-PEDRP-----TAKE------

**Pr80778**  ---------------------------------KRV--MPAFH------------SD----CPR--AILDLARTCLSYD-PDDRP-----SAKE------

**S15848T0**  ---------------------------------GKL--TPTFR------------DD----CPP--PVRALAEQCLAMD-PTGRP-----TASQ------

**S15849T0**  ---------------------------------GKL--TPTFR------------DD----CPS--DILALAKECLAMD-PDDRP-----TAAH------

**S15852T0**  ---------------------------------GKL--TPTFR------------DD----CPS--DILALAKDCLAMD-PDDRP-----TAAH------

**S01258T0**  ---------------------------------GEM--TPAFD------------AT----SPS--WVQEIGLRCMALD-PDARP-----TALE------

**S09257T0**  ---------------------------------GRL--QPTLNG-----------PN----VPS--WVPEMAKKCLQLH-EEDRP-----TSLM------

**S15543T0**  ---------------------------------GRL--QPTLNG-----------PN----VPS--WVPEMAKKCLQLH-VEDRP-----TSLE------

**S09298T0**  ---------------------------------GEL--RPTFEQ-----------YE----TPV--WVRDLGMQCMALN-PDDRP-----TSLA------

**S15453T0**  ---------------------------------GEL--RPTFEQ-----------YE----TPV--WVRDLGMQCMALD-PDDRP-----TSLA------

**S08727T0**  ---------------------------------GKI--RPTMDL-----------ST----TPT--WVTDLALQCMATN-PDDRP-----SIMV------

**S09308T0**  ---------------------------------GEL--VPDFDW-----------AR----SPI--WLIEIAQLCLATE-PEKRP-----TALA------

**S01416T0**  ---------------------------------GSV--KPTFS------------DE----MPE--WLRTLALQCIETE-PENRP-----TAMY------

**S06128T0**  ---------------------------------GSL--KPTFS------------AT----MPS--WLRDVAAQCVASE-PNDRP-----TAYK------

**S12836T0**  ---------------------------------GTI--EPTFS------------DA----CPA--PLRALATRCTLRI-ATDRP-----AAED------

**S17043T0**  ---------------------------------GTL--SPSFS------------TD----CPQ--WVHAIALRCLEHD-PQLRP-----SARQ------

**S08987T0**  ---------------------------------GTI--SPSFV------------AS----TPM--WVITLAQSCLAHA-PEHRP-----TAIE------

**S13082T0**  ---------------------------------GTL--QPTFG------------AS----SPT--WVVELANDCIALT-ADDRP-----TAIQ------

**S05673T0**  ---------------------------------GKI--KPTLS------------ET----CPD--WLREMALECMAFS-PDDRP-----TAVD------

**S09620T0**  ---------------------------------GKI--KPTLS------------ET----CPD--WLREMALECMAFS-PDDRP-----TAVD------

**S12826T0**  ---------------------------------GEL--TPTFT------------ST----CPA--WLEALATRCIDLT-PQNRP-----TAAD------

**S03267T0**  ---------------------------------GQL--KPKFG------------AS----CPP--SLVALADLCLHPN-PKARP-----AAHV------

**S13910T0**  ---------------------------------GNA--TLEFR------------DD----CPP--AVRSMADRCLAYD-PRDRP-----SAKD------

**Pi05338T0**  ---------------------------------APL--QPKFR------------DD----CPP--EILDIAHSCLKDD-PADRP-----TAME------

**Pr80780**  ---------------------------------TAP--KPKFR------------ED----CPP--DILEIAQKCLQDA-PADRP-----TAME------

**Ps133281**  ---------------------------------TAF--KPKFR------------DD----CPP--EILEIAQSCLRDD-PAERP-----TAME------

**Pi22892T0**  ---------------------------------GKL--HPVFT------------LG----CPT--GVRELAERCLSLE-TADRP-----TALQ------

**Pr94275**  ---------------------------------GKL--HPVFT------------LG----CPT--GVRELAERCLSFE-ASDRP-----TALQ------

**Ps135323**  ---------------------------------GKL--HPVFT------------LG----CPT--GVRELAERCLSYD-ASERP-----TALQ------

**S01930T0**  ---------------------------------GRA--VPSFG------------DD----CPD--VVSRIGRQCLQLD-PSKRP-----PIAS------

**Ps133943**  ---------------------------------GAL--TPTLS------------PA----CPK--SIVKLVNACTSYK-PKYRP-----SSTQ------

**S03896T0**  ---------------------------------DLL--QPTFS------------VE----CPI--DVQDVAKKCLAHC-AEDRP-----SAVQ------

**S09249T0**  ---------------------------------GDV--APVFT------------PA----CPS--WVQSLATRCLSPN-PLDRP-----SAVT------

**Pi03775T0**  ----------------------------------KT--GFRLS------------RQ----CSG--VLKEVITACVEKD-PARRP-----SMAE------

**Pr75312**  ----------------------------------KT--GFRLS------------RL----CSG--VMKEIIAACLEKD-PARRP-----SMAD------

**Ps136506**  ----------------------------------KT--GFRLS------------YL----CSG--VMKETISACLEKD-PARRP-----SMAD------

**AT3G14840_LRRXIV**  ---------------------------------QEK--EPVMNILDQSLLVDEDLLE----EVW--AMAIVARSCLNPK-PTRRP-----LMRH------

**Ps141628**  ---------------------------------HKGSA--SGD------------AE----APT--PVRLLMFRCQAFQ-PEGRP-----TADE------

**Pu08017**  ---------------------------------TRGEVIPVVG------------GR----SPV--PLRILTMNCISFQ-PEDRP-----SAQE------

**S14923T0**  ---------------------------------PTA--MPHEDHETF--------LR----APD--AFTQLVSACLDWD-PCMRP-----TAMS------

**S14924T0**  ---------------------------------DRDAKSTEYPFSDDVEFES---------CPK--PFRLLMLSCLQRD-VLKRP-----LAVS------

**Mb37167**  ---------------------------------GG---RPSLD------------EG----LLA--ALRDKTTGTTSSNGPVPRP-----HSAHDGKRDH

**AtAME2**  -----------------------------------ATSRDSLKAVWKLPRLPNLIMQHVDHSAG--DLIDLLQGLLRYD-PTERF-----KARE------

**hCLK1**  ---------------------------------GRYVSRRCKPLKEFMLSQDVEHE----------RLFDLIQKMLEYD-PAKRI-----TLRE------

**AtCDC2a**  --------------------------------------KSAFPKWKPTDLETFVPN-----LDP--DGVDLLSKMLLMD-PTKRI-----NARA------

**hCDK3**  --------------------------------------KGSFPKWTRKGLEEIVPN-----LEP--EGRDLLMQLLQYD-PSQRI-----TAKT------

**AtMPK1**  ---------------------------------------RSLPYSPGMSLSRLYPG-----AHV--LAIDLLQKMLVFD-PSKRI-----SVSE------

**hMAPK1**  ---------------------------------------LSLPHKNKVPWNRLFPN-----ADS--KALDLLDKMLTFN-PHKRI-----EVEQ------

**AtGSK3b**  --------------------------------------DFRFPQIKAHPWHKVFHKR----MPP--EAIDLASRLLQYS-PSLRC-----TALE------

**AtGSK3i**  --------------------------------------DFRFPQIKAHPWHKVFHKR----MPP--EAIDLASRLLQYS-PSLRC-----TALE------

**AtCKA1**  --------------------------------------EALVGRHSRKPWSKFINADNQHLVSP--EAIDFLDKLLRYD-HQDRL-----TAKE------

**hCK2a**  --------------------------------------NDILGRHSRKRWERFVHSENQHLVSP--EALDFLDKLLRYD-HQSRL-----TARE------

**AtMEKK1**  --------------------------------------LPEVPDT----------------LSL--DARLFILKCLKVN-PEERP-----TAAE------

**hMAPKKK1**  ---------------------------------ASATTAPSIPSH----------------LSP--GLRDVALRCLELQ-PQDRP-----PSRE------

**AtMKK3**  --------------------------------------SPTPPKQE---------------FSP--EFCSFIDACLQKD-PDARP-----TADQ------

**hMAPKK1**  ---------------------------------------PKLPSGV---------------FSL--EFQDFVNKCLIKN-PAERA-----DLKQ------

**AtCPK7**  -------------------------------------------------------------VSD--SAKDLVRKMLEPD-PKKRL-----TAAQ------

**hCaMK1**  -------------------------------------------------------------ISD--SAKDFIRHLMEKD-PEKRF-----TCEQ------

**AtNPH1**  -------------------------------------------------------------ASL--QVKQLIFRLLQRD-PKKRLGCFE-GANE------

**AtPVPKlikePK5**  -------------------------------------------------------------VSF--AARDLIRGLLMKE-PQQRLGFKR-GATE------

**AtS6KlikePK1**  -------------------------------------------------------------LSN--EAHAILKGLLQKE-PERRLGSGLSGAEE------

**hGRK6**  -------------------------------------------------------------FSP--QARSLCSQLLCKD-PAERLGCRGGSARE------

**AtSnRK2**  -------------------------------------------------------------LSP--ECRHLISRIFVAD-PATRI-----TIPE------

**OUTGROUP1**  -------------------------------------------------------------LATHPTLVPLVMRLLEKE-PSRRL-----QSAH------

**OUTGROUP2**  -------------------------------------------------------------LPN--SLDNIIHRATAKL-PSERY-----QSVE------

**OUTGROUP3**  -------------------------------------------------------------VPQ--SVKNIVLQATMKN-PNERY-----ISSK------

**OUTGROUP4**  -------------------------------------------------------------LSA--DLDAVVLKALAKN-PENRY-----QTAA------

**AtTousled**  --------------------------------------KVEFPVTRPA-------------ISN--EAKDLIRRCLTYN-QEDRP-----DVLT------

**hTousledLK1**  --------------------------------------EVQFPVKPV--------------VSS--EAKAFIRRCLAYR-KEDRF-----DVHQ------

**S08428T0**  ---------------------------------------------------------------------ALLASCTASD-PTHRI-----DASA------

**S08432T0**  -------------------------------------------------------ND----MPE--TICAIVASCMALE-PTTRP-----SALE------

**Pi03773T0**  VNNGRMLQSSRSLALEENRRRTTPVLGTHNNDETSFMNMFTFT------------PE----CPT--MVKELAGACLQYD-PSLRP-----SASY------

**Pr75310**  SNSSRTLQSSRSVGLEENRRRTTPLLGAHNNDDTSFMGMFTFT------------PE----CPT--MVRELAVACLQYD-PSLRP-----SASY------

**Ps136501**  SNSSRTLQSSRSLALEENRRRTTPVLGTHNNDETSLMSMFTFT------------PE----CPT--MVRELAGACLQYD-PSLRP-----SASY------

**Pu07161**  ---------------------------------TSLMSMFPFT------------DE----CPP--MVKELASACLQYD-PSLRP-----SASY------

**S09505T0**  ---------------------------------LQTASDPTAAFPDEVDITSLLGLYAFDTCPA--IIKAVAKSCVHPD-PMQRP-----SASF------

**AT1G14390_LRRVI-1**  ----------------------------------------DEPSVLRSLADPCVRGTYAYESLR--TTVEFAINCLCED-QRKRP-----SIED------

**AT5G41180_LRRVI-2**  --------------------------CLVEWVREKNLGAPDVMASLVDPELKHFKQK----ELE--AVCEVASQCLNLD-QNEKDKDKLSCSIQ------

**hTGFbRI**  ---------------------------------QKL--RPNIPNRWQS-------------CEALRVMAKIMRECWYAN-GAARL-----TALR------

**hTGFbRII**  ---------------------------------DRG--RPEIPSFWLN-------------HQGIQMVCETLTECWDHD-PEARL-----TAQC------

**Pi14992T0**  NAAMHHFMSKLRMLANIGGTDVEGDKSSFGTTSTTSAASLTVS------------LSSSSAIVD--KLSKAIEDCLQSD-PKKRP-----TAKK------

**Ps157753**  NAAMHHFMSKLRMLANIGGTDAEADESSFGTTPTSSAASLTVS------------LSSSSAIVD--KLSGAIEDCLQSD-PKKRP-----TAKK------

**Pi04971T0**  ---------------------------------GGNAHLLKFS------------AD----CPE--ELQELARQCWHEV-PSERP-----DAID------

**Ps136072**  ---------------------------------GGDPHLLKFS------------AD----CPL--DLQELARRCWNEV-PSRRP-----DAID------

**Pr93648**  ---------------------------------GGNPHLLKFS------------TD----CPL--ELQELARQCWSEV-PSQRP-----DAID------

**Pu03658**  ---------------------------------GEEHHLLKFT------------PE----CPN--EIKYLARRCWNPN-IYERP-----DALD------

**AtCKI1**  -------------------------------------------------------------YPS--EFTSYFHYCRSLR-FEDKP-----DYSY------

**hCKIalpha2**  -------------------------------------------------------------FPA--EFAMYLNYCRGLR-FEEAP-----DYMY------

**mCKIalpha**  -------------------------------------------------------------FPA--EFAMYLNYCRGLR-FEEAP-----DYMY------

**910 920 930 940 950**

**....|....|....|....|....|....|....|....|....|....|....|..**

**AT1G06840_LRRVIII-1** -----------------------------------VVRE-----L------------

**AT4G29180_LRRI**  -----------------------------------IVSD-----L------------

**AT3G14840_LRRVIII-2** -----------------------------------VVSM-----L------------

**AtNIK1_LRRII**  -----------------------------------VVRM-----L------------

**AtNAK**  -----------------------------------IVKT-----M------------

**AT2G24230_LRRVII**  -----------------------------------VVGL-----L------------

**AtBRI1_LRRXb**  -----------------------------------VMAM-----F------------

**AtCLV1_LRRXI**  -----------------------------------VVHM-----L------------

**AtFEI1_LRRXIIIa**  -----------------------------------VVQL-----L------------

**AtER_LRRXIIIb**  -----------------------------------VTRV-----LGSF---------

**AtRPK1_LRRXV**  -----------------------------------AVRL-----L------------

**AtTMK1_LRRIX**  -----------------------------------AVNI-----LSSL---------

**AT3G28450_LRRXa**  -----------------------------------AYQS-----L------------

**AtIMK3_LRRIII**  -----------------------------------VMTQ-----L------------

**AT2G45340_LRRIV**  -----------------------------------VIQE-----L------------

**AtSCM_SUB_LRRV**  -----------------------------------IVQD-----L------------

**AtFLS2_LRRXII**  -----------------------------------ILTH-----L------------

**dmPELLE**  -----------------------------------VLKR-----F------------

**drIRAK1**  -----------------------------------VFKA-----L------------

**hIRAK1**  -----------------------------------ENSY-----V------------

**xtPELLE**  -----------------------------------VFLT-----L------------

**AtCTR1_Raf**  -----------------------------------IMDL-----L------------

**Mb27170**  -----------------------------------AEAM-----LKQI---------

**Mb28586**  -----------------------------------IVQT-----L------------

**hRaf1**  -----------------------------------ILSS-----I------------

**Mb37485**  -----------------------------------VTNT-----L------------

**Esi0009_0077**  -----------------------------------VLAD-----F------------

**Esi0009_0083**  -----------------------------------NVRK-----I------------

**Esi0020_0071**  -----------------------------------VLEG-----M------------

**Esi0173_0029**  -----------------------------------LSGGEK---V------------

**Ch136568**  -----------------------------------LVER-----L------------

**Ch50123**  --------------------------------------------M------------

**Ch137597**  -----------------------------------ALRR-----L------------

**Ch137605**  -----------------------------------ALQR-----L------------

**Ch56654**  -----------------------------------VLQR-----L------------

**Ch141610**  -----------------------------------VLQQ-----L------------

**Ch143410**  -----------------------------------VLQR-----L------------

**Ch141452**  -----------------------------------VLQR-----L------------

**Mb10450**  -----------------------------------VLHE-----L------------

**Mb25375**  -----------------------------------LLIT-----L------------

**Mb28923**  -----------------------------------VLMQ-----L------------

**Mb37923**  -----------------------------------VCAT-----L------------

**Mb34096**  -----------------------------------IVNA-----L------------

**Mb36839**  -----------------------------------TRHT-----L------------

**Mb36426**  -----------------------------------IVLE-----M------------

**hAXL**  -----------------------------------LRED-----L------------

**hHGFR**  -----------------------------------LVSR-----I------------

**hRYK**  -----------------------------------LVQC-----L------------

**hDDR**  -----------------------------------LHRF-----LAEDALNTV----

**hIR**  -----------------------------------IVNL-----L------------

**hLTK**  -----------------------------------ILER-----L------------

**hMuSK**  -----------------------------------IHRI-----LER-----M----

**hTRKalpha**  -----------------------------------VHAR-----L------------

**hKLGlikePTK7**  -----------------------------------IASA-----L------------

**hFGFR2**  -----------------------------------LVED-----L------------

**hRET**  -----------------------------------ISKD-----L------------

**hVGFR1**  -----------------------------------LVEK-----L------------

**hPDGFRbeta**  -----------------------------------LVLL-----L------------

**hTIE1**  -----------------------------------IALQ-----L------------

**hEGFR**  -----------------------------------LIIE-----F------------

**hEPH**  -----------------------------------LQAH-----L------------

**Pi00640T0**  -----------------------------------VVLT-----I------------

**Pi19256T0**  -----------------------------------VVLT-----I------------

**Pr72884**  -----------------------------------IVLT-----I------------

**Ps140986**  -----------------------------------VVLT-----I------------

**Pi00643T0**  -----------------------------------IVVY-----L------------

**Pr72888**  -----------------------------------IVEY-----L------------

**Ps140988**  -----------------------------------IVVY-----L------------

**Pi00646T0**  -----------------------------------VVYA-----V------------

**Ps140991**  -----------------------------------VVYA-----I------------

**Pr72890**  -----------------------------------VVYA-----I------------

**Pi17831T0**  -----------------------------------IAAA-----MQHL---------

**Ps132644**  -----------------------------------IVMI-----L------------

**Pi17840T0**  -----------------------------------IVVI-----LEHM---------

**Pr76005**  -----------------------------------IVVI-----LEHM---------

**Ps132640**  -----------------------------------IVVI-----L------------

**Pi17832T0**  -----------------------------------VVET-----L------------

**Pr76010**  -----------------------------------IVET-----L------------

**Ps132643**  -----------------------------------IVDT-----L------------

**Pi05112T0**  -----------------------------------ISYE-----LRKIMKDELX---

**Pr82847**  -----------------------------------ISYE-----LRKIMKDELX---

**Ps132270**  -----------------------------------ISYE-----LRKIMKDELX---

**Pu14970**  -----------------------------------ISYE-----L------------

**S05981T0**  -----------------------------------LSYL-----L------------

**Pr81631**  -----------------------------------VAYE-----L------------

**Pi02124T0**  -----------------------------------VLQT-----L------------

**Pr73421**  -----------------------------------VLQT-----L------------

**Ps144068**  -----------------------------------VLQT-----L------------

**S04928T0**  -----------------------------------IAAS-----L------------

**Pi07724T0**  -----------------------------------IAYE-----LR-----------

**Ps158080**  -----------------------------------IAYE-----LRLI---------

**Pr77475A**  -----------------------------------IAHF------------------

**Pi07725T0**  -----------------------------------IAYE-----L------------

**Pi21299T0**  -----------------------------------IAYE-----LRRRWL-------

**Pr77476**  -----------------------------------IAYE-----L------------

**Ps140003**  -----------------------------------IAYE-----L------------

**Pi07731T0**  -----------------------------------IAYE-----L------------

**Ps139996**  -----------------------------------IAYE-----L------------

**Ps139997**  -----------------------------------IAYE-----L------------

**Pr77470**  -----------------------------------IAYE-----LRLI---------

**Pr77475B**  -----------------------------------IAYE-----LRRM---------

**Pi09664T0**  -----------------------------------VAYE-----LR-----------

**Pr75814**  -----------------------------------VAYE-----LR-----------

**Ps135332**  -----------------------------------VAYE-----LR-----------

**Ps135334**  -----------------------------------VAYE-----LR-----------

**Pi23090T0**  -----------------------------------VAYV-----L------------

**Pi23143T0**  --------------------------------------T-----L------------

**Pr81229**  -----------------------------------IAYV-----LRTV---------

**Pr75791**  -----------------------------------IAYA-----LRTL---------

**Pi09665T0**  -----------------------------------VAFA-----L------------

**Ps135333**  -----------------------------------VAFA-----L------------

**Ps127796**  -----------------------------------LVYA-----L------------

**Pi13397T0**  -----------------------------------AHYE-----LRTL---------

**Ps140146**  -----------------------------------AHYE-----LRTL---------

**Pr81472**  -----------------------------------AHYE-----LRTL---------

**Pi14970T0**  -----------------------------------LHYQ-----L------------

**Ps138087B**  -----------------------------------LHYQ-----LRSIANACQDVVM

**Pr75692**  -----------------------------------LHYQ-----L------------

**Pi14971T0**  -----------------------------------LHF-------------------

**Pi14972T0**  -----------------------------------IHYE-----L------------

**Pr75691A**  -----------------------------------IHYE-----L------------

**Ps138088**  -----------------------------------IHYE-----L------------

**S06057T0**  -----------------------------------VGRL-----L------------

**S02178T0**  -----------------------------------LRAW-----F------------

**S02311T0**  -----------------------------------VLSW-----L------------

**S02316T0**  -----------------------------------VWAY------------------

**S13911T0**  -----------------------------------VYTW-----LHHL---------

**Pi05340T0**  -----------------------------------LWRL-----L------------

**Ps133283**  -----------------------------------LWRL-----L------------

**Ps133286**  -----------------------------------LWRL-----L------------

**Pr80778**  -----------------------------------LWRS-----L------------

**S15848T0**  -----------------------------------AAYE-----L------------

**S15849T0**  -----------------------------------VAFA-----L------------

**S15852T0**  -----------------------------------VAYA-----L------------

**S01258T0**  -----------------------------------LYVL-----L------------

**S09257T0**  -----------------------------------LASM-----L------------

**S15543T0**  -----------------------------------LASM-----L------------

**S09298T0**  -----------------------------------LTAI-----LQRV---------

**S15453T0**  -----------------------------------LTAI-----LQRV---------

**S08727T0**  -----------------------------------VAAM-----V------------

**S09308T0**  -----------------------------------LAGL-----L------------

**S01416T0**  -----------------------------------LAAV-----L------------

**S06128T0**  -----------------------------------LAHI------------------

**S12836T0**  -----------------------------------LVHE-----L------------

**S17043T0**  -----------------------------------LVLE-----L------------

**S08987T0**  -----------------------------------IAHT-----I------------

**S13082T0**  -----------------------------------VAHR-----I------------

**S05673T0**  -----------------------------------LEAR-----L------------

**S09620T0**  -----------------------------------LEAR-----L------------

**S12826T0**  -----------------------------------LVCE-----L------------

**S03267T0**  -----------------------------------VVVD-----L------------

**S13910T0**  -----------------------------------VYKW-----I------------

**Pi05338T0**  -----------------------------------LHYS-----F------------

**Pr80780**  -----------------------------------LHYS-----L------------

**Ps133281**  -----------------------------------LHYS-----L------------

**Pi22892T0**  -----------------------------------AVVV-----L------------

**Pr94275**  -----------------------------------TV--------------------

**Ps135323**  -----------------------------------AVIV-----L------------

**S01930T0**  -----------------------------------IVEA-----L------------

**Ps133943**  -----------------------------------VQQD-----L------------

**S03896T0**  -----------------------------------MEYW------------------

**S09249T0**  -----------------------------------IQHE-----L------------

**Pi03775T0**  -----------------------------------VVLA-----F------------

**Pr75312**  -----------------------------------VALA-----L------------

**Ps136506**  -----------------------------------VALA-----F------------

**AT3G14840_LRRXIV**  -----------------------------------IVQA-----L------------

**Ps141628**  -----------------------------------LLEE-----L------------

**Pu08017**  -----------------------------------ALDE-----L------------

**S14923T0**  -----------------------------------VVAT-----L------------

**S14924T0**  -----------------------------------VAEE-----LRRELLL------

**Mb37167**  QTDEDEDEDLSLLLDALDEDDECEELGRGSLPARPAPAEPQ---A------------

**AtAME2**  -----------------------------------ALNHPF---F------------

**hCLK1**  -----------------------------------ALKHPF---F------------

**AtCDC2a**  -----------------------------------ALEHEY---F------------

**hCDK3**  -----------------------------------ALAHPY---F------------

**AtMPK1**  -----------------------------------ALQHPY---M------------

**hMAPK1**  -----------------------------------ALAHPY---L------------

**AtGSK3b**  -----------------------------------ACAHPF---F------------

**AtGSK3i**  -----------------------------------ACAHPF---F------------

**AtCKA1**  -----------------------------------AMAHAY---F------------

**hCK2a**  -----------------------------------AMEHPY---F------------

**AtMEKK1**  -----------------------------------LLNHPF---V------------

**hMAPKKK1**  -----------------------------------LLKHPV---F------------

**AtMKK3**  -----------------------------------LLSHPF---I------------

**hMAPKK1**  -----------------------------------LMVHAF---I------------

**AtCPK7**  -----------------------------------VLEHTW---I------------

**hCaMK1**  -----------------------------------ALQHPW---I------------

**AtNPH1**  -----------------------------------VKQHSF---F------------

**AtPVPKlikePK5**  -----------------------------------VKQHPF---F------------

**AtS6KlikePK1**  -----------------------------------IKQHKW---F------------

**hGRK6**  -----------------------------------VKEHPL---F------------

**AtSnRK2**  -----------------------------------ITSDKW---F------------

**OUTGROUP1**  -----------------------------------ELADAL----------------

**OUTGROUP2**  -----------------------------------ELLREL----------------

**OUTGROUP3**  -----------------------------------ELFEDLSTVL------------

**OUTGROUP4**  -----------------------------------EMRADL---V------------

**AtTousled**  -----------------------------------MAQDPY---L------------

**hTousledLK1**  -----------------------------------LANDPY---L------------

**S08428T0**  -----------------------------------VASW-----L------------

**S08432T0**  -----------------------------------VATR-----L------------

**Pi03773T0**  -----------------------------------IAAM-----L------------

**Pr75310**  -----------------------------------IAAM-----L------------

**Ps136501**  -----------------------------------IAAM-----L------------

**Pu07161**  -----------------------------------VVAM-----L------------

**S09505T0**  -----------------------------------AYAM-----M------------

**AT1G14390_LRRVI-1**  -----------------------------------VVWN-----L------------

**AT5G41180_LRRVI-2**  -----------------------------------ALCET----L------------

**hTGFbRI**  -----------------------------------IKKT-----L------------

**hTGFbRII**  -----------------------------------VAER-----F------------

**Pi14992T0**  -----------------------------------LVDL-----F------------

**Ps157753**  -----------------------------------LSPE-----M------------

**Pi04971T0**  -----------------------------------VQEE-----L------------

**Ps136072**  -----------------------------------VQEE-----L------------

**Pr93648**  -----------------------------------VQEE-----L------------

**Pu03658**  -----------------------------------LQEE-----L------------

**AtCKI1**  -----------------------------------LRRLFRDLFI------------

**hCKIalpha2**  -----------------------------------LRQL-----F------------

**mCKIalpha**  -----------------------------------LRQL-----F------------
