# Supplementary material for: Leucine-Rich repeat receptor kinases are sporadically distributed in eukaryotic genomes
Source: BMC Evol Biol. 2011 Dec 20;11:367. doi: 10.1186/1471-2148-11-367 (PMC3268121; doi:10.1186/1471-2148-11-367)

**Additional file 5: Details of the maximum likelihood tree representing phylogenetic relationships among the LRR-RK KDs and other eukaryotic kinases**.


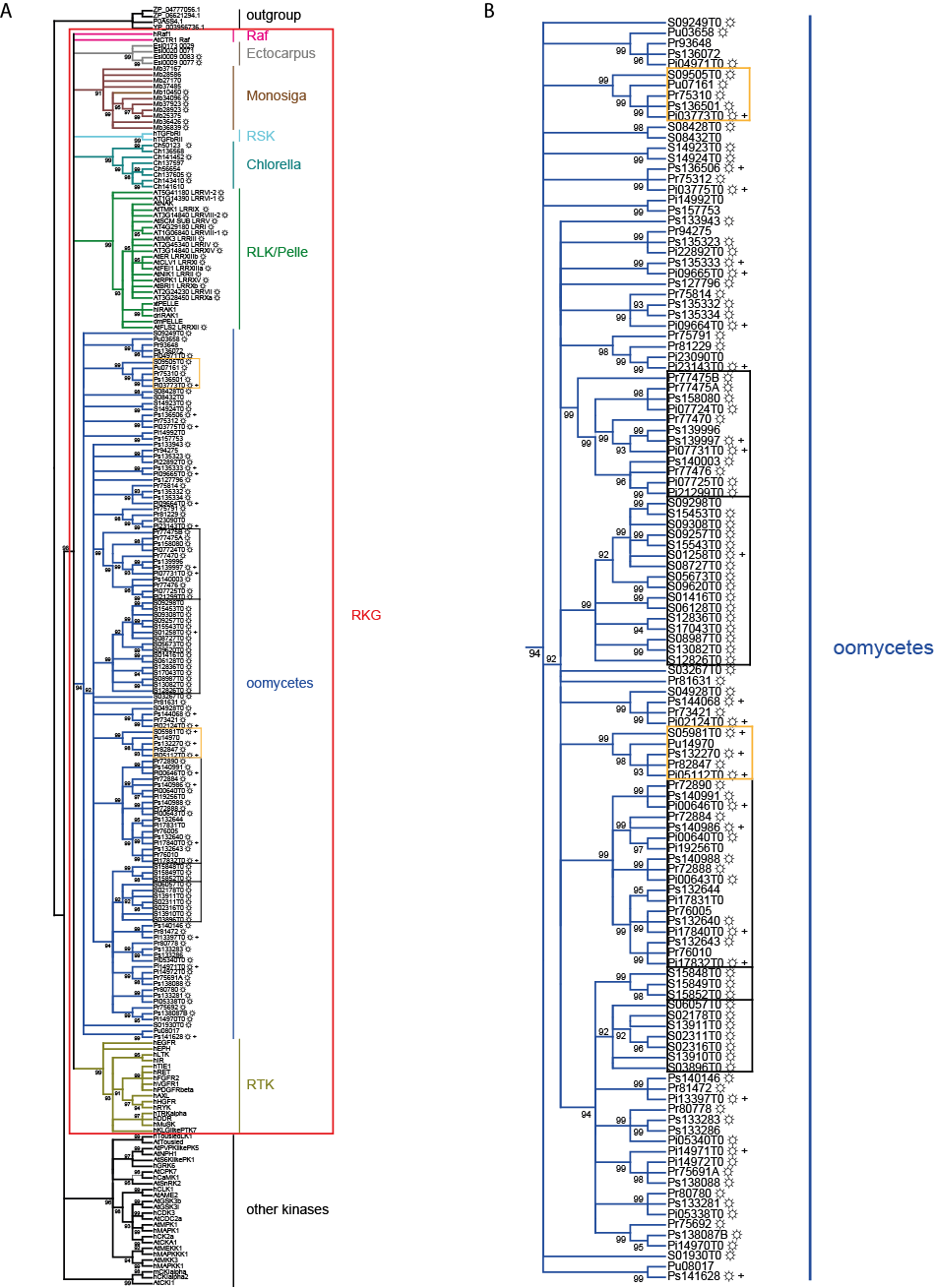


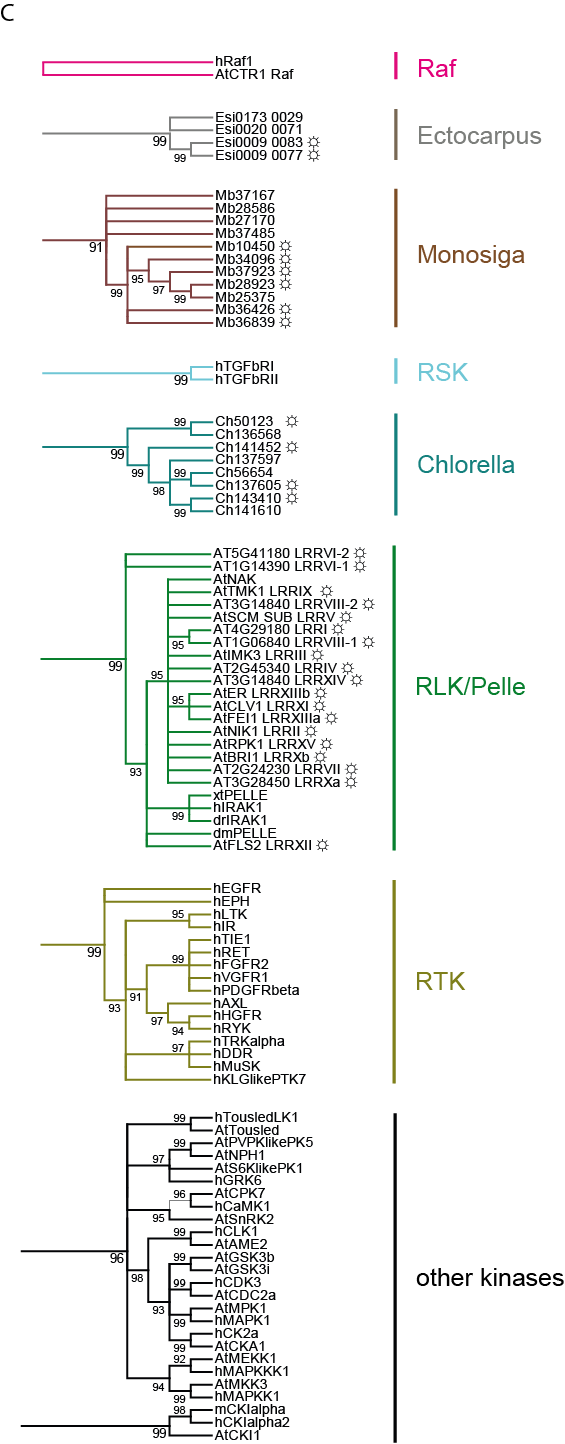

Supplement: Additional file 5 — Details of the maximum likelihood tree representing the phylogenetic relationships among the LRR-RK KDs and other eukaryotic kinases. A. General view of the tree presented in Figure 2. The phylogenetic tree was generated from an alignment of the KDs (Additional file 4) of representative Arabidopsis and animal protein kinases, LRR-containing RKs (☼) and closely related receptors lacking TM domains and/or LRRs. These sequences, devoid of LRRs and/or TM domains, have been included to highlight the versatility of these domains with respect to their associations with KDs. The tree branches are colored as follows: Monosiga brevicollis (Monosiga, brown); Chlorella variabilis NC64A (Chlorella, greenish blue); Ectocarpus siliculosus (Ectocarpus, gray) and oomycetes (oomycetes, blue). Oomycete LRR-RKs, Monosiga LRR-RKs, Chlorella LRR-RKs and Ectocarpus LRR-RKs are included in the monophyletic receptor kinase group (RKG, red box) consisting of the plant receptor-like kinase (RLK) and animal cytoplasmic Pelle (forming the RLK/Pelle subfamily, green), receptor serine/threonine kinase (RSK, light blue), receptor tyrosine kinase (RTK, khaki) and Raf (Raf, pink) proteins. The RKG members are distinct from other the eukaryotic kinases. Note that the tree is not fully resolved. The addition of more RKs lacking LRRs to this phylogenetic analysis did not improve the resolution of the tree (data not shown); similarly, neither did the addition of non-RKs from the oomycete species (data not shown). Branch support values are shown at the nodes. B. A detailed view of the oomycete clade. In the oomycete LRR-RK subfamily, some subdivisions are Saprolegnia or Phytophthora specific (black boxes), suggesting the lineage-specific amplification by duplications in the Saprolegnia and Phytophthora genomes. Three subgroups contain both of these lineages with or without Pythium (orange boxes), suggesting that at least three genes were present in the last common ancestor of these species. Oomycete gene [file 1471-2148-11-367-S5.DOC]
