# Supplementary material for: Leucine-Rich repeat receptor kinases are sporadically distributed in eukaryotic genomes
Source: BMC Evol Biol. 2011 Dec 20;11:367. doi: 10.1186/1471-2148-11-367 (PMC3268121; doi:10.1186/1471-2148-11-367)
Supplement: Additional file 6 — Number of gains and losses of oomycete LRR-RK genes in orthologous groups. We counted the number of genes gained and lost for each orthologous group defined in the oomycete clades of the phylogenetic tree (Additional file 5B). A. Orthologous groups containing Saprolegnia, Pythium and Phytophthora species, B. orthologous groups containing only Pythium and Phytophthora species and C. orthologous groups containing only Phytophthora species. The number on the first branch represents the number of genes present in the last common ancestor. Numbers preceded by a plus sign are the number of genes gained; numbers preceded by a minus sign are the number of genes lost. [file 1471-2148-11-367-S6.DOC]

**Additional file 6: Number of gains and losses of oomycete LRR-RK genes in orthologous groups**.
