# Supplementary material for: Leucine-Rich repeat receptor kinases are sporadically distributed in eukaryotic genomes
Source: BMC Evol Biol. 2011 Dec 20;11:367. doi: 10.1186/1471-2148-11-367 (PMC3268121; doi:10.1186/1471-2148-11-367)
Supplement: Additional file 7 — Oomycete LRR-RK subgroups with evidence of expression. Abbreviations: S, Saprolegnia parasitica; Pi, Phytophthora infestans; Ps, Phytophthora sojae; Pp, Phytophthora parasitica; Pp, Phytophthora parasitica; EST, expressed sequence tags. [file 1471-2148-11-367-S7.DOC]

**Additional file 7: Oomycete LRR-RK subgroups with evidence of expression**
