# Supplementary material for: Leucine-Rich repeat receptor kinases are sporadically distributed in eukaryotic genomes
Source: BMC Evol Biol. 2011 Dec 20;11:367. doi: 10.1186/1471-2148-11-367 (PMC3268121; doi:10.1186/1471-2148-11-367)
Supplement: Additional file 8 — Phytophthora parasitica LRR-RKs with evidence of expression. Abbreviations: Pi, Phytophthora infestans; EST, expressed sequence tags. [file 1471-2148-11-367-S8.DOC]

**Additional file 8: *Phytophthora parasitica* LRR-RKs with evidence of expression**
